# From predefined indicators to a management dashboard for heart failure telemonitoring: a modified nominal group technique approach in Portuguese hospitals

**DOI:** 10.1186/s12913-026-14031-1

**Published:** 2026-01-23

**Authors:** Rafael Miranda, David Franco Rodrigues, Filipa Matos Baptista, Mónica Duarte Oliveira

**Affiliations:** 1 CEGIST, Instituto Superior Técnico, Universidade de Lisboa, Lisbon, Portugal; 2 Enterprise Services, Siemens Healthineers Portugal, Lisbon, Portugal; 3 iBB- Institute for Bioengineering and Biosciences and i4HB- Associate Laboratory Institute for Health and Bioeconomy, Instituto Superior Técnico, Universidade de Lisboa, Lisbon, Portugal

**Keywords:** Healthcare dashboards, Collaborative approach, Nominal group technique, Heart failure, Telemonitoring

## Abstract

**Background:**

Clinical, operational, and financial data drive healthcare decisions, but deriving insights from complex datasets is challenging and error-prone. Dashboards simplify analytics, yet adoption is constrained by skepticism about generic solutions designed without user input. While participatory methods have allowed consultation in dashboard-building tasks, collaboration between users is required to ensure consensual and needs-oriented tools.

**Methods:**

We modified the nominal group technique – leveraging *a priori* idea generation and iterative voting through literature review, questionnaire, and workshop processes – to engage prospective users in creating consensual dashboards from predefined key performance indicators (KPIs). First, sets of suitable visuals for each KPI are identified through a literature-informed decision table. Next, users individually state their preferential visuals via an online questionnaire. Finally, a collaborative dashboard-building workshop enables users to (1) discuss and select final KPI visuals and (2) integrate selected visuals into a prototype dashboard. Two real-world cases (UC1 and UC2) in telemonitoring for heart failure (HF) management in Portuguese hospitals are reported.

**Results:**

UC1 reports the collaborative analysis of 23 KPIs on HF telemonitoring program’s *Case-mix*, *Access* and *Clinical aspects* by health professionals and medical technology providers (*n* = 6), co-creating three dashboard pages. In UC2, cardiologists (*n* = 5) validated UC1’s choices, assessed 12 new KPIs on *Acceptability* and *Costs*, and a functional dashboard prototype comprising five pages (average 7.2 visuals per page) was developed. While both cases adhere to a common KPI set, visuals reflect each telemonitoring protocol and are tailored to users’ management needs and program objectives.

**Conclusions:**

The proposed approach revealed well-structured while expedite, aligning health professionals and managers' needs with developers' design perspectives. It allows translating narrative management concepts into interactive and actionable information. Moreover, by fostering user collaboration, dashboard ownership, trust, usability, adoption, and workflow integration are enhanced.

**Supplementary Information:**

The online version contains supplementary material available at 10.1186/s12913-026-14031-1.

## Background

The digitalization of healthcare has led to massive data generation, causing a “data overload” that hinders managers’ ability to extract meaningful information and make quick decisions [1]. As data complexity and volume grow, the demand for advanced tools to provide actionable insights intensifies [2, 3]. Healthcare organizations increasingly rely on dashboards [4, 5] to transform sparse data into interactive visualizations (e.g., graphs and charts) [6], supporting continuous monitoring of key performance indicators (KPIs) [7] and improving communication among diverse stakeholders – including physicians, nurses, technicians, managers and patients [8] – by bridging knowledge gaps in context, syntax and semantics [9].

Dashboards can play a critical role in patient monitoring, aiding the real-time detection of emerging issues and prompting timely interventions [10–12]. Beyond clinical decision support, these data visualization (DataViz) tools can help assess hospital performance, manage operations and implement strategy by integrating fragmented data and aligning clinical, operational and financial analyses [13, 14]. Current uses include operating room optimization [15], emergency department flux management [16], radiology dose tracking [17], chronic care support [18], public health emergency response [19], or population health surveillance [20], among others.

Despite the potential to support clinical decision-making and management, dashboard adoption remains inconsistent due to challenges in understanding and validating information, varying digital literacy among user groups, and unfamiliarity with dashboard interfaces [21, 22]. To create dashboards that meet user needs and avoid obsolescence, it is essential to engage prospective users and stakeholders throughout the development process [23, 24]. Active involvement builds a sense of ownership, which increases the likelihood of long-term adoption [25, 26], while participatory and collaborative approaches foster social cohesion in developing a unified tool that balances multiple perspectives [27, 28].

Some studies have used participatory methods – interviews, surveys, design sessions, and workshops – to gather input and promote debate in dashboard development tasks like setting requirements [29–32], selecting KPIs and performance measures [16, 33–35], designing DataViz [36, 37], testing dashboards [38, 39] and evaluating usability [18, 40, 41]. Few engage prospective users across tasks [42–46]. Notwithstanding progress in participatory dashboard building, a notable gap remains in collaboratively selecting KPI DataViz and co-creating cohesive dashboard pages. User collaboration in these tasks is particularly important, as individual DataViz choices and further layout design greatly impact how effectively the desired information is communicated [47].

## Methods

### Study design

Our study addresses two related challenges: (1) while healthcare data is abundant, insightful and actionable information remain scarce; dashboards are a promising solution, but (2) their impact is limited when users are not engaged in the development process. We developed a novel collaborative dashboard-building (CDB) approach that modifies the traditional nominal group technique (NGT) by combining a literature review, a pre-workshop questionnaire, and a CDB workshop to actively engage users in creating effective, cohesive, and consensual dashboard prototypes from predefined KPIs.

NGT is a structured brainstorming method, facilitating equal participation and generating a prioritized list of ideas or solutions [48–52]. Originating for conducting potentially problematic group sessions, NGT became a popular technique for structuring stakeholder collaboration [50, 51]. NGT traditionally involves five phases [53]: *idea generation* (silent development of ideas to solve a task statement), *round robin* (idea sharing in turns), *clarification* (sequential discussion and refinement of ideas), *voting* (ranking or scoring top ideas), *final discussion* (results analysis and commenting).

While many NGT studies follow these five phases, others employ modified versions. Common modifications include using literature-informed ideas [54], eliciting items before meetings [49, 55], anonymous idea elicitation or ranking via digital tools [54, 55], online NGT [53, 55, 56], roundtable brainstorming [49], voting/rating instead of ranking [49, 57], iterative voting [49], and group review with individual re-ranking [48, 54]. Our proposed CDB approach also adheres to a modified NGT, employing a priori and literature-informed idea generation and (a)synchronous online iterative voting, allowing to adapt flexibly to the unique needs of different dashboard user groups and facilitating voting and discussion processes across in-person, online, and hybrid study settings.

### Proposed approach

**Fig. 1:**
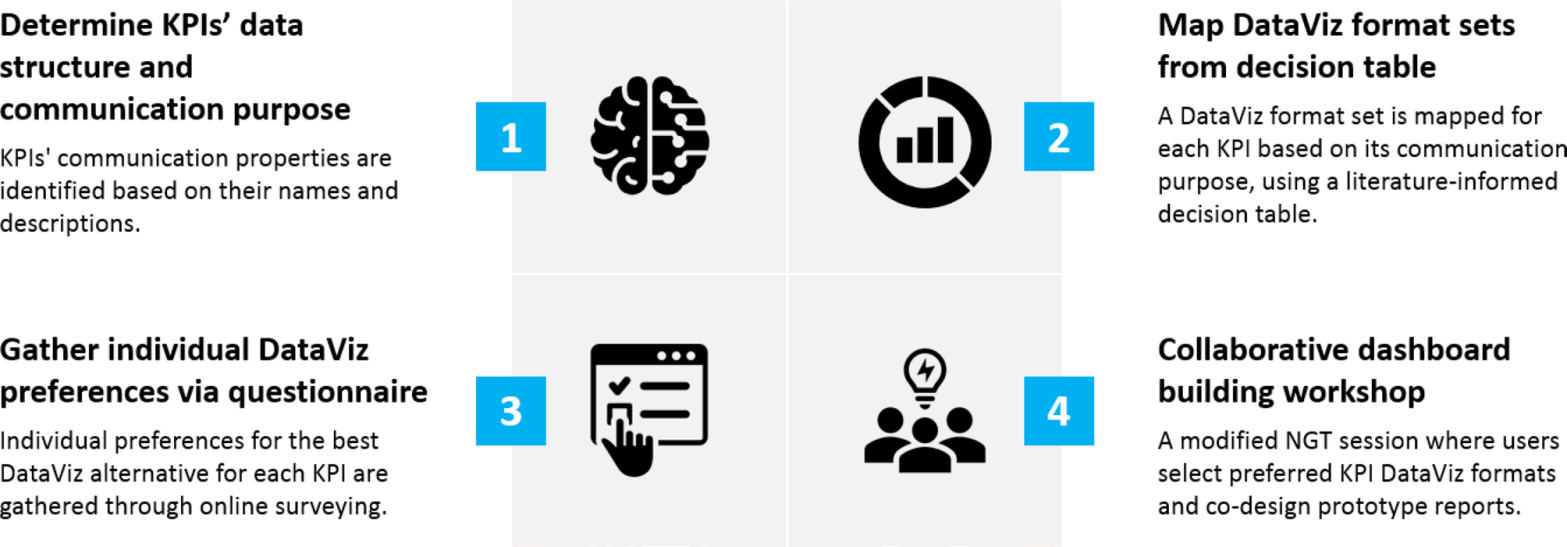
Four-stage collaborative dashboard-building approach

Figure 1 summarizes the proposed four-stage CDB approach. In Stage 1, the predefined KPI’s data structure and communication purpose are determined. In Stage 2, a set of suitable DataViz formats is mapped for each KPI using a decision table. Stage 3 involves collecting individual preferences for the best DataViz alternative for each KPI through an online questionnaire. Stage 4 proposes a CDB workshop where dashboard stakeholders and end-users (*a*) reflect, discuss, and vote on preferred DataViz formats and (*b*) collaborate in designing prototype dashboard pages using the selected formats.

#### Stage 1: KPIs’ data structure and communication purpose

Following Ignatenko et al. [37], KPI’s data structures are represented as strings, where the length indicates the number of variables, and “C” or “Q” denotes categorical (i.e., nominal or ordinal e.g., disease classification, age groups) or quantitative (i.e., numerical) types, respectively. Illustrating, “CQ” implies one categorical and one quantitative variable (e.g., *Number of alerts generated* [Q] *and severity of alerts* [C]). Once the data structure is identified, KPIs are analyzed to determine their intended communication purpose. According to Kirk’s taxonomy [58], six communication purposes are allowed (see the first column in Table 1).

**Table 1:** Decision table for data visualization format identification (Q – 1 categorical variable, QC − 1 quantitative, 1 categorical variable, QQ − 2 quantitative variables, QCC − 1 quantitative, 2 categorical variables, QQC − 2 quantitative, 1 categorical variable, QQQ − 3 quantitative variables)

Data structure	Communication purpose					
	Q	QC	QCC	QQ	QQC	QQQ
Assessing hierarchies and part-to-whole relationships	-	Pie Chart, (100%) Stacked Bar Chart	Circle Packing Diagram, ^a^ (100%) Stacked Bar Chart	-	Waterfall Chart ^b^	-
Comparing categories	-	Area Size Chart, ^a^ Bar Chart, Word Cloud ^a^	Dot Plot, ^a^ Grouped Bar Chart, Two-sided Bar Chart	Histogram ^a^	Floating Bar, ^a^ Gantt Chart, ^a^ Waterfall Chart ^b^	-
Displaying dimensionless measures	(Linear) Gauge, ^b^ KPI ^b^	-	-	-	-	-
Mapping geospatial data	-	-	Dorling Cartogram ^a^	Dot Plot Map ^a^	Network Connection Map ^a^	Bubble map, Cartogram, ^a^ Choropleth map, Dasymetric map, Point map
Plotting connections and relationships	-	-	-	Scatter Plot	-	Bubble Plot
Showing changes over time	-	Bar Chart	Grouped Bar Chart, (100%) Stacked Bar Chart	Area Chart, Line Chart, Sparklines ^a^	(Stacked ^a^) Area Chart, Line Chart, Streamgraph ^a^	-

Notes: (^a^) Additional formats identified in [58]; (^b^) Additional formats identified in [59].

#### Stage 2: KPI’s DataViz sets

Our approach modifies NGT’s *idea generation* by pre-selecting voting alternatives (i.e., DataViz “ideas” to solve the task statement “What is the most appropriate DataViz format to represent [KPI name]?*”*). Instead of asking NGT participants to freely suggest alternatives, we identify sets of suitable and commonly used DataViz formats – based on the communication purpose and data structure of each KPI – using a literature-informed decision table (see Table 1). Table 1 extends the decision table from Ignatenko et al. [37] by adding formats from Kirk’s book [58] and by including newer options enabled by modern dashboard technologies (e.g., formats for dimensionless variables [59]).

#### Stage 3: Online questionnaire

Stakeholders and end-users vote for their preferred alternatives via an online questionnaire that showcases the mock-ups designed to display the DataViz sets identified for each KPI in Stage 2. This procedure represents another modification to traditional NGT, using digital tools to facilitate anonymous voting *ex ante* the CDB workshop. Respondents analyze the DataViz sets one by one, vote for their preferred alternative, provide feedback, and suggests alternative formats if needed. The questionnaire platform supports structured survey design, easy sharing of mock-ups, commenting, and automatic aggregation of results.

#### Stage 4: Collaborative dashboard-building workshop

After completing the questionnaire, dashboard stakeholders and end-users are invited to a facilitated CDB workshop. Facilitators here act as process consultants, constructively and responsively using their acquired expertise to help participants solve their own problems [60]. While in-person workshops are preferred [53], hybrid or online options are allowed based on scheduling and logistics considerations.

In a first phase, participants engage for *clarification* (i.e., sequential discussion) of questionnaire responses, new *voting*, and *final discussion*. The aggregated questionnaire votes and comments are reviewed for the first KPI, followed by a group discussion on the optimal DataViz format. A dashboard-building software presents visual mock-ups (default Stage 2 sets plus new formats suggested in the questionnaire). Participants vote again (individually), and the results are analyzed silently. Anonymous comments are allowed to encourage feedback towards a final (consensual) format selection. This process repeats for each KPI.

After reaching agreement on the optimal DataViz format for all KPIs, the group moves to the second phase: co-creating prototype dashboard pages. The facilitation team builds pages for each aggregated KPI level (e.g., evaluation dimension or thematic area), arranging DataViz formats into prototype layouts. Participants review these prototypes for coherence, ease of use, and alignment with user needs. If necessary, alternative KPI formats (e.g., second most voted) are tested for better visual cohesion. Stakeholders also contribute input on presentation elements (e.g., colors, logos, fonts) and analysis features (e.g., reference levels, drill-down, filters).

### Study setting

Two use cases (UC) in Portuguese public hospitals are reported in the Results section: UC1 at Hospital do Espírito Santo de Évora (HESE), in the Alentejo region, and UC2 at Hospital de Santa Maria (HSM), in Lisbon. Both UC1 and UC2 focus on developing dashboards for managing heart failure (HF) telemonitoring programs. HF is a chronic condition characterized by symptoms of fatigue and breathlessness, and frequently leads to decompensation episodes [61]. HF management requires close surveillance, where telemonitoring facilitates symptom tracking, patient-physician interaction and therapy engagement [62]. Successful remote care implementation demands real-time insights into program performance, emphasizing the need for data aggregation, processing and visualization tools [63].

Both cases build on an expert-agreed list [34] of 43 KPIs across five program performance dimensions and six *Case-mix* parameters for continuous HF telemonitoring assessment. Such KPIs were selected through a participatory approach encompassing interviews and a web-Delphi process with Portuguese HF telemonitoring stakeholders. Notably, the stakeholders for both UC1 and UC2 participated in this participatory process (co-designed by RM, MDO and FMB), ensuring context continuity.

## Results

### UC1 – Dashboard design for *Case-mix*, *Access* and *Clinical* KPIs at HESE

In UC1, we showcase the implementation of the proposed approach in selecting user-preferred DataViz formats for a predetermined list of HF telemonitoring KPIs [34]. This process led to the selection of preferred DataViz formats for monitoring *Case-mix*, *Access* and *Clinical aspects* indicators of the HF telemonitoring program at HESE. In this case we implemented Stages 1, 2, and 4, with the online questionnaire (Stage 3) not being performed due to time constraints, and the option being to carry out an extended CDB workshop.

#### Stage 1 findings

Table 2 outlines the data structure and communication purpose for each KPI related to *Case-mix*, *Access* and *Clinical aspects* (see Table 5 in [34] for KPI descriptions). For KPIs described as occurring “within the program duration”, monthly breakdowns were assumed. Most KPIs serve ongoing tactical and strategic performance assessment, by *Showing changes over time*. However, KPIs tied to assessment scales or population classes allow *Assessing hierarchies and part-to-whole relationships*, e.g., *Case-mix* KPIs.

**Table 2:** Key performance indicators’ data structure and communication purpose

Key performance indicator (within dimension)	Variables	Data structure	Communication purpose
**Access**			
Eligible patients followed by the RPM program (%)	Current value (versus Reference)	Q	Displaying dimensionless measures
Length of stay in the ward	Average number per Month	QC	Showing changes over time
Length of stay in intensive care	Average number per Month	QC	Showing changes over time
Number of days of activity lost	Average number per Month/Year	QC	Showing changes over time
Number of HF-related emergency visits	Total number per Month	QC	Showing changes over time
Number of HF-related hospitalisations	Total number per Month	QC	Showing changes over time
Number of HF-related readmissions	Total number per Month	QC	Showing changes over time
Number of scheduled face-to-face consultations	Average number per Month	QC	Showing changes over time
Number of unscheduled face-to-face consultations	Average number per Month	QC	Showing changes over time
Number of scheduled teleconsultations	Average number per Month	QC	Showing changes over time
Number of unscheduled teleconsultations	Average number per Month	QC	Showing changes over time
Waiting time for a face-to-face consultation	Average time per Month	QC	Showing changes over time
Waiting time for a teleconsultation	Average time per Month	QC	Showing changes over time
Time to medical action	Average time per Severity	QC	Comparing categories
**Clinical aspects**			
Avoidable hospital admissions due to HF compared to the homologous period (%)	Current value (versus Reference)	Q	Displaying dimensionless measures
Biosignals	Average number over threshold per Date	QQ	Showing changes over time
Left ventricular ejection fraction (LVEF)	Total number per Class	QC	Assessing hierarchies and part-to-whole relationships
HF-related/All-cause mortality ratio	Current value (versus Reference)	Q	Displaying dimensionless measures
Number of alerts generated and severity of alerts	Total number per Severity	QC	Assessing hierarchies and part-to-whole relationships
Level of physical activity	Total number per Class	QC	Assessing hierarchies and part-to-whole relationships
NT-ProBNP level (pg/ml)	Total days over threshold per Month	QC	Showing changes over time
Mental health self-perception	Total number per Class	QC	Assessing hierarchies and part-to-whole relationships
Oedema self-perception	Total number per Class	QC	Assessing hierarchies and part-to-whole relationships
Quality of life self-perception	Total number per Class	QC	Assessing hierarchies and part-to-whole relationships
**Acceptability**			
Patient adherence to the program	Compliance ratio per Month	QC	Showing changes over time
Patient satisfaction	Total number per Class	QC	Assessing hierarchies and part-to-whole relationships
Health professional satisfaction	Total number per Class	QC	Assessing hierarchies and part-to-whole relationships
Caregiver overload	Total number per Class	QC	Assessing hierarchies and part-to-whole relationships
Disease management capacity after the program	Total number per Class	QC	Assessing hierarchies and part-to-whole relationships
Level of self-care	Total number per Class	QC	Assessing hierarchies and part-to-whole relationships
Patient’s trust in the program	Total number per Class	QC	Assessing hierarchies and part-to-whole relationships
Medication/therapy adherence	Average number per Month	QC	Showing changes over time
Number of program dropouts	Total number per Month	QC	Showing changes over time
**Costs**			
Program cost per patient	Average cost per Month per Cost component	QCC	Showing changes over time
Costs for the patient	Total cost per Month	QC	Showing changes over time
Emergency service admission costs	Total cost per Month	QC	Showing changes over time
Hospital admission costs	Total cost per Month	QC	Showing changes over time
ICU hospitalization costs	Total cost per Month	QC	Showing changes over time
Surgical intervention costs	Total cost per Month	QC	Showing changes over time
Face-to-face consultations costs	Total cost per Month	QC	Showing changes over time
Teleconsultation costs	Total cost per Month	QC	Showing changes over time
**Technology**			
Number of implantable device events	Total number per Device type per Event type	QCC	Comparing categories
Error rate (%)	Current value (versus Reference)	Q	Displaying dimensionless measures
**Case-mix parameters**			
Age group	Total number per Class	QC	Assessing hierarchies and part-to-whole relationships
Classification of HF according to LVEF	Total number per Class	QC	Assessing hierarchies and part-to-whole relationships
Comorbidities	Total number per Condition	QC	Comparing categories
Distance to the nearest healthcare facility	Total number per Class	QC	Assessing hierarchies and part-to-whole relationships
Literacy level	Total number per Class	QC	Assessing hierarchies and part-to-whole relationships
NYHA classification	Total number per Class	QC	Assessing hierarchies and part-to-whole relationships

Notes: C = categorical; Q = quantitative; HF = heart failure; ICU = intensive care unit; NYHA = New York Heart Association; RPM = remote patient monitoring

#### Stage 2 findings

Table 3 shows the corresponding DataViz format sets conveyed for each KPI (or composite KPI) by the decision table (Table 1). In cases where KPIs are interrelated or a joint analysis might benefit users (e.g., *Length of stay in the ward* and *Length of stay in intensive care*), composite KPIs were allowed. As a result, the data structure and communication purpose were adjusted accordingly, as reflected in Table 3.

**Table 3:** Updated key performance indicators (KPIs) list (considering composite KPIs) and corresponding visualization format sets

Composite indicator	Short name	Key performance indicator	Data structure	Data visualization formats set
**Access**				
Eligible patients followed by the RPM program (%)	% Patients	=	Q	(Linear) Gauge KPI Bar Chart (if per Month)
Length of stay	LoS	Length of stay in the ward Length of stay in intensive care	QCC	Grouped Bar Chart (100%) Stacked Bar Chart Bar Chart (for each KPI)
Number of days of activity lost	DAL	=	QC	Bar Chart (Linear) Gauge (if Q) Area Chart (if QQ) Line Chart (if QQ)
Number of HF-related admissions	Admissions	Number of HF-related emergency visits Number of HF-related hospitalisations Number of HF-related readmissions	QCC	Grouped Bar Chart (100%) Stacked Bar Chart Bar Chart (for each KPI)
Number of consultations	Cons.	Number of scheduled face-to-face consultations Number of unscheduled face-to-face consultations Number of scheduled teleconsultations Number of unscheduled teleconsultations	QCC	Grouped Bar Chart (100%) Stacked Bar Chart Bar Chart (for each KPI) Waterfall Chart (if QQC and *Comp. categories*)
Waiting time	=	Waiting time for a face-to-face consultation Waiting time for a teleconsultation	QCC	Grouped Bar Chart (100%) Stacked Bar Chart Bar Chart (for each KPI)
Time to medical action	Time to MD	=	QC	Area Size Chart Bar Chart Word Cloud Grouped Bar Chart (if QCC)
**Clinical aspects**				
Avoidable hospital admissions due to HF compared to the homologous period (%)	Avoidable	=	Q	(Linear) Gauge KPI Area Chart (if QQ) Bar Chart (if QC)
Biosignals	=	=	QQ	Area Chart Line Chart Sparklines Bar Chart (if QC)
Left ventricular ejection fraction (LVEF)	LVEF	=	QC	Pie Chart (100%) Stacked Bar Chart Bar Chart (if per Month)
HF-related/All-cause mortality ratio	% HF death	=	Q	(Linear) Gauge KPI Area Chart (if QQ) Bar Chart (if QC)
Number of alerts generated and severity of alerts	Alerts	=	QC	Pie Chart (100%) Stacked Bar Chart Grouped Bar Chart (if per Month)
Level of physical activity	Physical	=	QC	Pie Chart (100%) Stacked Bar Chart Bar Chart (if *Comp. categories*)
NT-ProBNP level (pg/ml)	NT-ProBNP	=	QC	Bar Chart (Linear) Gauge (if Q) Area Chart (if QQ) Line Chart (if QQ)
Mental health self-perception	Mental health	=	QC	Pie Chart (100%) Stacked Bar Chart Bar Chart (if *Comp. categories*)
Oedema self-perception	Oedema	=	QC	Pie Chart (100%) Stacked Bar Chart Bar Chart (if *Comp. categories*)
Quality of life self-perception	QoL	=	QC	Pie Chart (100%) Stacked Bar Chart Bar Chart (if *Comp. categories*)
**Acceptability**				
Overall compliance	Compliance	Patient adherence to the program Medication/therapy adherence Number of program dropouts	QQC	(Stacked) Area Chart Line Chart Streamgraph
Stakeholder satisfaction	Satisfaction	Patient satisfaction Caregiver overload Health professional satisfaction	QCC	Dot Plot Grouped Bar Chart Two-sided Bar Chart (100%) Stacked Bar Chart (if *Showing changes over time*)
Disease management capacity after the program	Awareness	=	QC	Pie Chart (100%) Stacked Bar Chart Bar Chart (if *Comp. categories*)
Level of self-care	Self-care	=	QC	Pie Chart (100%) Stacked Bar Chart Bar Chart (if *Comp. categories*)
Patient’s trust in the program	Trust	=	QC	Pie Chart (100%) Stacked Bar Chart Bar Chart (if *Comp. categories*)
**Costs**				
Program cost per patient	€ Avg	=	QCC	Grouped Bar Chart (100%) Stacked Bar Chart
Costs for the patient	€ OoP	=	QC	Bar Chart Area Chart (if QQ) Line Chart (if QQ) Sparklines (if QQ)
Total program cost	€ Total	Emergency service admission costs Hospital admission costs ICU hospitalization costs Surgical intervention costs Face-to-face consultations costs Teleconsultation costs	QCC	Grouped Bar Chart (100%) Stacked Bar Chart Waterfall Chart (if QQC and *Comp. categories*) KPI (for each KPI)
**Technology**				
Number of implantable device events	ID events	=	QCC	Dot Plot Grouped Bar Chart Two-sided Bar Chart
Error rate (%)	% Error	=	Q	(Linear) Gauge KPI Bar Chart (if per Month)
**Case-mix parameters**				
Age group	Age	=	QC	Pie Chart (100%) Stacked Bar Chart
Classification of HF according to LVEF	LVEF class	=	QC	Pie Chart (100%) Stacked Bar Chart
Comorbidities	=	=	QC	Area Size Chart Bar Chart Word Cloud
Distance to the nearest healthcare facility	Distance	=	QC	Pie Chart (100%) Stacked Bar Chart
Literacy level	Literacy	=	QC	Pie Chart (100%) Stacked Bar Chart
NYHA classification	NYHA	=	QC	Pie Chart (100%) Stacked Bar Chart

Notes: C = categorical; Q = quantitative; HF = heart failure; ICU = intensive care unit; KPI = key performance indicator; NYHA = New York Heart Association; RPM = remote patient monitoring

**Fig. 2:**
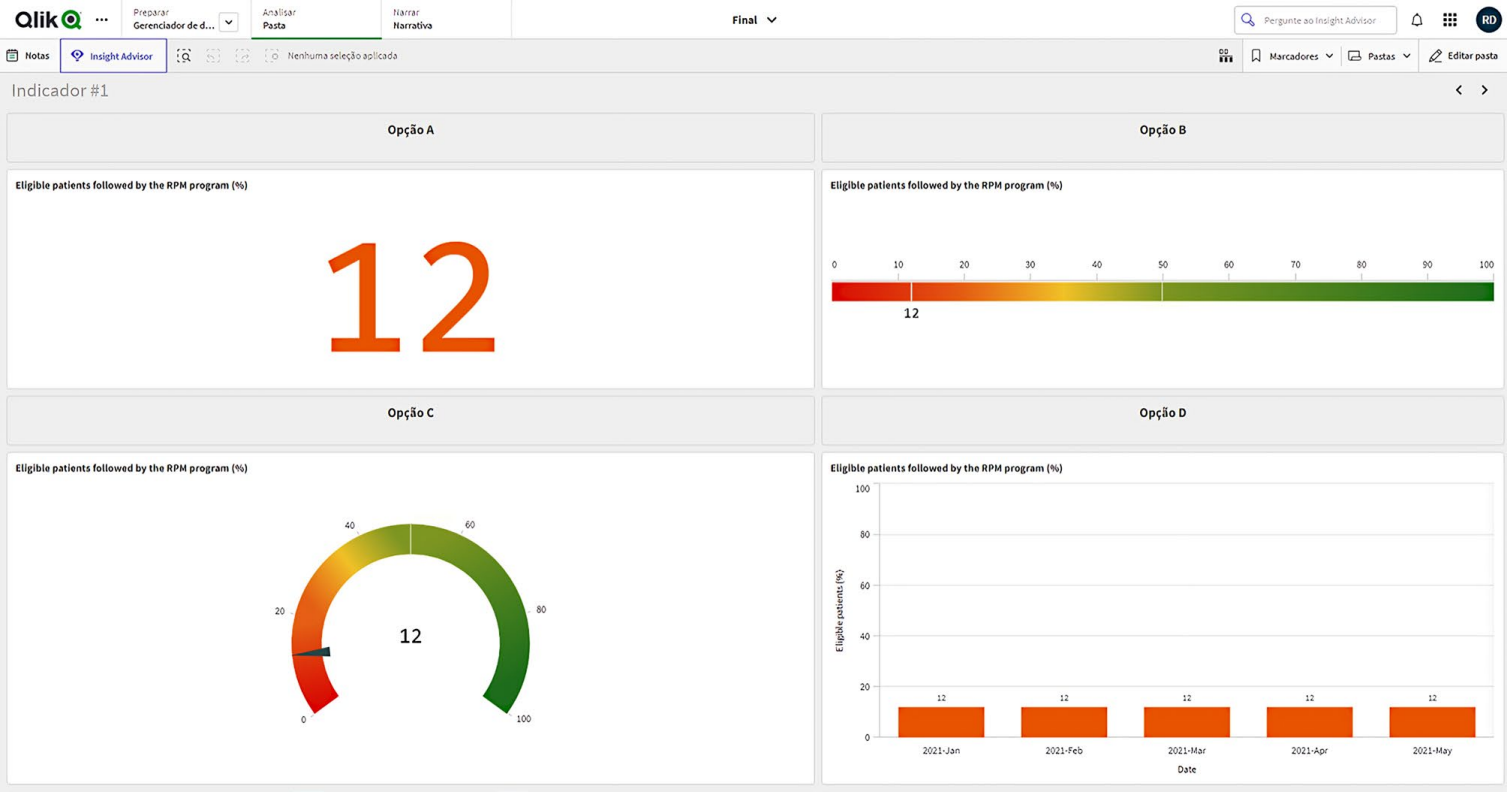
Qlik Sense^®^ page example for *Eligible patients followed by the remote patient monitoring (RPM) program (%)*

DataViz mock-ups were generated in Qlik Sense^®^, for improved visualization and appraisal during the CDB workshop. Dashboard pages displayed up to four alternative mock-ups for one or more (i.e., composite) of the 17 *Access* and *Clinical aspects* KPIs (note: *Acceptability* and *Costs* KPIs, covered in Tables 2 and 3, were excluded from UC1’s scope by project stakeholders). Figure 2 illustrates the mock-ups for the *% Patients* DataViz set. A prototype dashboard page was designed for the *Case-mix*, proposing a single DataViz format per KPI.

#### Stage 4 implementation and findings

The CDB workshop was held at HESE in Évora, Portugal, on September 13, 2023, lasting 2h38. Six HF telemonitoring program stakeholders (two females, four males) were invited to participate, including a physician/program coordinator, two HF nurses (one female), a telemonitoring technology company owner and their application specialist (female), and a healthcare consultant. Invited stakeholders were purposely identified by the program coordinator as relevant actors within the HF telemonitoring care protocol and operations, and all accepted to participate.

**Fig. 3:**
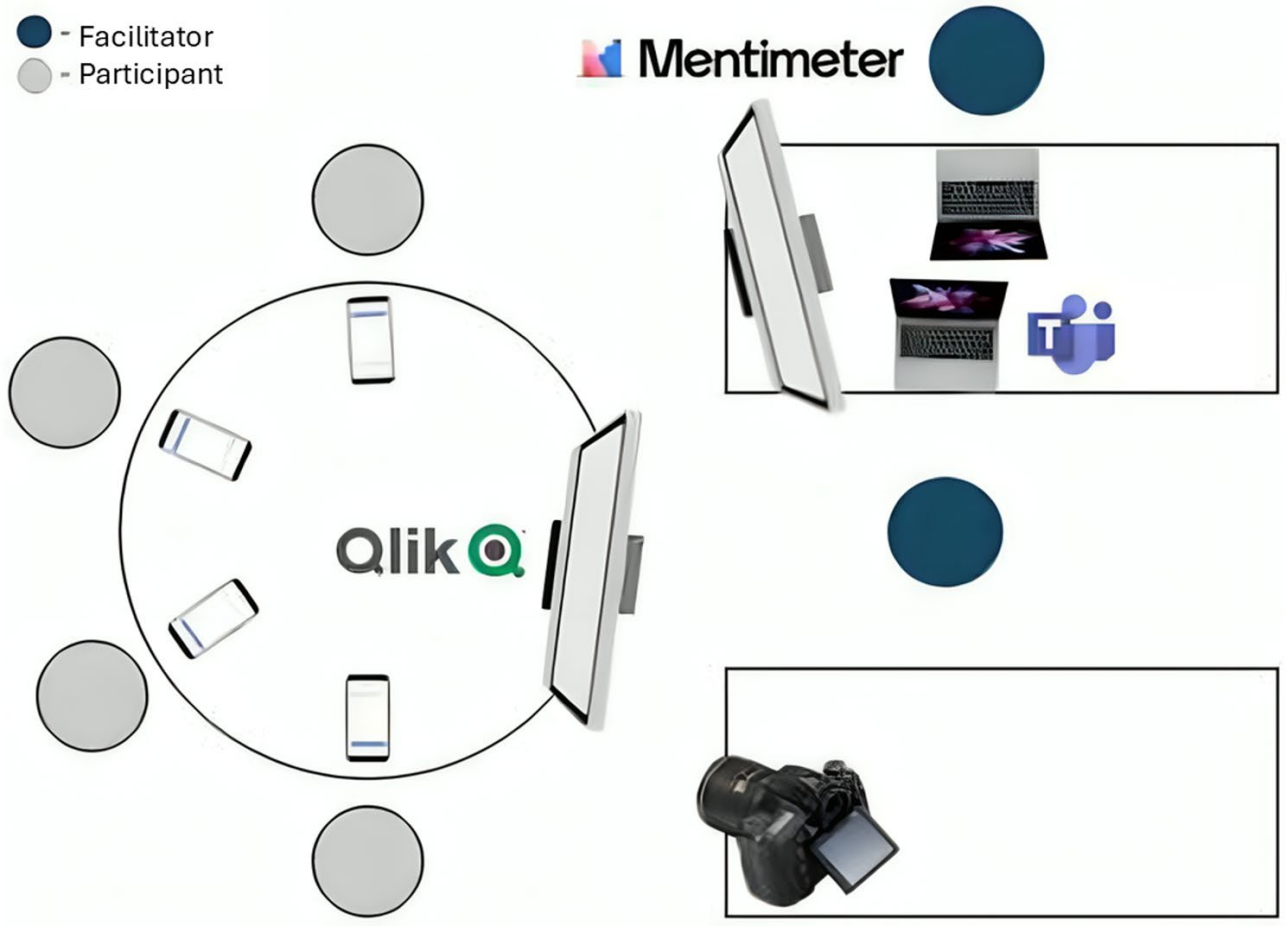
Layout schematic for the workshop room setup, showing seating arrangement, screen positioning and employed support systems

Initially planned as an in-person session, unforeseen constraints led to a hybrid format, with two participants attending via Microsoft Teams™. As depicted in Fig. 3, onsite participants sat at a round table facing the facilitators (RM, a PhD student in decision analysis with experience in HF telemonitoring projects, and DR, an MSc student in computer science) and two screens – one displaying voting results through Menti™ (https://www.menti.com/) and the other showing DataViz alternatives on Qlik Sense^®^. Participants and facilitators provided voice consent for video recording before workshop start.

**Fig. 4:**
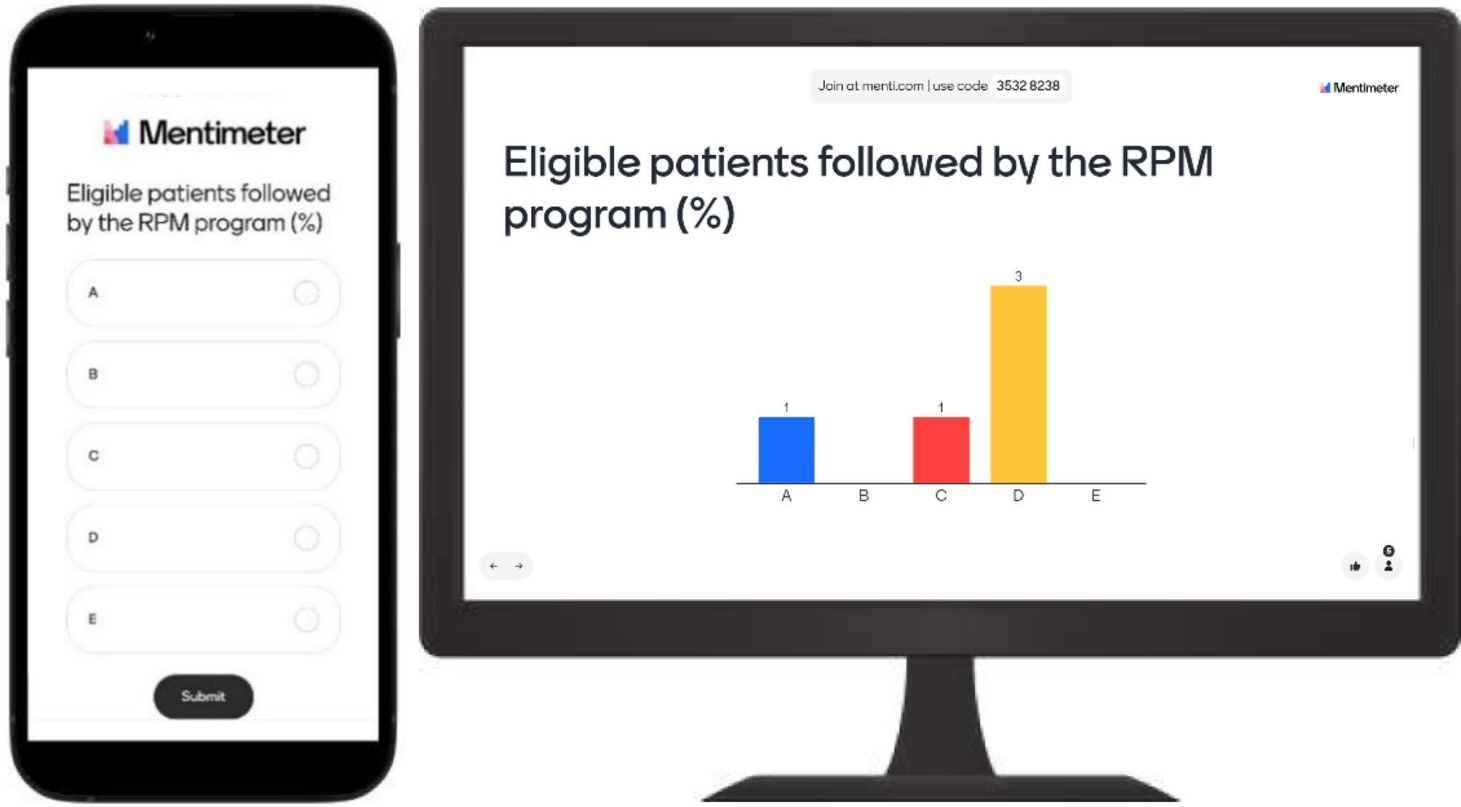
Menti™ mobile interface (left) and voting results (right), as presented to participants

At the workshop start, participants silently assessed the alternatives for the first KPI before anonymously voting on their preferred format through a Menti™ survey. Menti™ offered four voting options (A, B, C, D) corresponding to displayed DataViz alternatives and an additional option (E) to express disagreement with available alternatives. Figure 4 illustrates the Menti™ mobile interface and voting results display.

Voting was anonymous, with results revealed only after all votes were submitted. Participants then shared anonymous written comments before discussing as a group. Facilitators encouraged input on (dis)advantages, constraints, and alternative interpretations of each DataViz format. Those disagreeing with default formats could propose an alternative (option E in later voting). A second voting round followed the discussion to select the preferred DataViz format for each KPI. This process was repeated for all 17 KPIs. Table 4 summarizes the outcomes, showing voting shifts and the final selected formats with visual mock-ups.

**Table 4:** Complete list of voting results for every key performance indicator reviewed in the workshop

KPI	Round 1					Round 2					Most voted format	Data visualization format
	A	B	C	D	E	A	B	C	D	E		
Access												
% Patients	1	0	1	**3**	0						Bar Chart	
LoS	0	0	2	3	0	0	1	0	**5**	0	Stacked Bar Chart	
DAL											Not assessed (**Updated description**: Average days of absence or reduced activity due to HF-related needs [e.g., hospitalization, emergency room admissions, premature death].)	
Admissions	0	0	3	3	0	0	0	**5**	1	0	Grouped Bar Chart	
Cons.	1	1	2	0	2	0	1	1	0	**4**	Waterfall Chart + Time	
Waiting time	0	0	**4**	2	0						Grouped Bar Chart	
Time to MD											Not assessed (**Updated description**: Average time interval, expressed in hours and by alert severity, from a clinical alert signaling potential patient deterioration to medical doctor appropriate intervention.)	
**Clinical asp.**												
Avoidable											Not assessed (**Updated description**: Yearly count of preventable emergency department admissions [i.e., admitted patients with unresolved clinical alerts within the past 24 h].)	
Biosignals	1	0	0	0	5	0	0	0	0	**6**	Modify KPI (**Updated description**: Average monthly count of clinical alerts [i.e., when a patient exhibits two or more vital sign measurements outside their defined normal range]).	
LVEF											Not assessed (Participants suggested excluding this KPI, as LVEF is more relevant as a case-mix parameter i.e., *LVEF class*)	
% HF death	1	1	3	1	0	**3**	0	**3**	0	0	Area Chart	
Alerts	0	0	**6**	0	0						Grouped Bar Chart	
Physical	**4**	0	1	1	0						Pie Chart	
NT-ProBNP	**5**	1	0	0	0						Line Chart	
Mental health	4	1	0	1	0	2	**3**	0	1	0	Bar Chart	
Oedema	**5**	0	1	0	0						Pie Chart	
QoL	1	**5**	0	0	0						Bar Chart	

Notes: Cons. = consultations; DAL = days of activity lost; HF = heart failure; KPI = key performance indicator; LoS = length of stay; LVEF = left ventricular ejection fraction; MD = medical doctor; QoL = quality of life

In the first voting round, *% Patients* and *LoS* received only five votes due to a delayed online participant; all subsequent KPIs and rounds received six. Of the 17 KPIs discussed, *DAL*, *Time to MD*, *Avoidable*, and *LVEF* were excluded from voting due to description ambiguity, with participants providing input for clarification (see Table 4 for the updated KPI descriptions). Participants agreed to forego a second voting round for seven KPIs, as the first round had already established a majority and no compelling arguments for change emerged. In the remaining six cases, four exhibited a strengthened majority for the initially favored alternative, while two resulted in a shift in option preferences (including a tie for *% HF death*).

A two-thirds majority was reached for 10 out of 13 KPIs after the two voting rounds. *% Patients* and *Mental health* garnered a relative majority (three votes for the preferred alternative), and *% HF death* remained tied (three votes each for options A and C). An *Area Chart* was selected for *% HF death* as it received three votes in both rounds, with the option to revisit this choice during the prototype page appraisal. Modifications were proposed for *Biosignals*, as its DataViz alternatives were deemed unsuitable (option E was consensual).

**Fig. 5:**
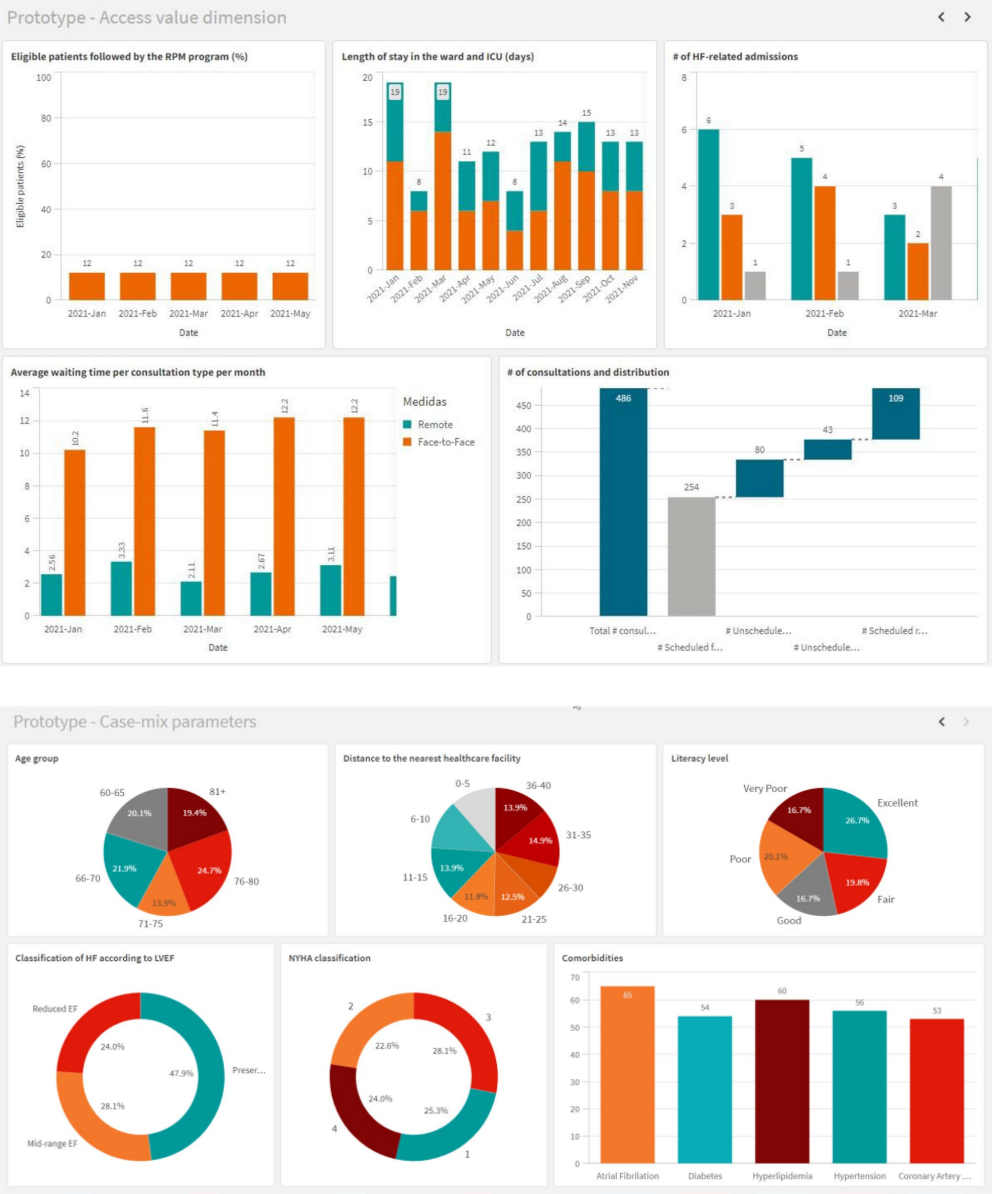
Prototype dashboard report pages for *Access* (top) and *Case-mix* (bottom)

During a timeout, the facilitators compiled prototype dashboard pages, which participants reviewed after the recess. Figure 5 displays the finalized prototypes.

For the *Access* page, participants retained all selected DataViz formats, making only minor color adjustments for consistency across KPIs. Since Qlik Sense^®^ does not support the requested *Waterfall Chart + Time* format for *Cons.*, it was suggested adding a month filter to enable similar analysis with a simple *Waterfall Chart*. For the *Case-mix* page, participants favored a combination of *Pie* and *Donut Charts* to enhance visual variety and cohesion. *Pie Charts* display demographic data (*Age*, *Distance*, *Literacy*), while *Donut Charts* represent HF-related information (*LVEF class*, *NYHA*). A *Bar Chart* was chosen over an *Area Size Chart* (initial proposal) for *Comorbidities*. To ensure clear theme separation, demographic data is placed in the top row and HF-related data in the bottom row. Due to time constraints, no prototype was created for *Clinical aspects*.

After the workshop, participants received an email acknowledging their active participation and a feedback survey. Five of six participants responded, finding the proposed approach user-friendly, being supportive of its use, and believing it could be beneficial for enhancing dashboard usefulness. Survey structure and results are detailed in Supplementary File 1.

### UC2 – Multidimensional dashboard for HF telemonitoring program management at HSM

In UC2, we tested whether UC1 results applied to a new hospital setting (HSM) while maintaining the same application domain and scope (i.e., HF telemonitoring with consistent dimensions and KPIs [34]). Five HSM cardiologists (two females, three males) were invited to validate UC1 choices for *Case-mix*, *Access*, and *Clinical aspects* and assess nine additional KPIs on program *Acceptability*, along with a prototype page for the *Costs* dimension. As in UC1, the program coordinator purposely selected stakeholders; however, only those involved in care processes were invited here, as HSM program’s technological infrastructure is fully outsourced. Only Stages 3 and 4 of the proposed approach are covered in the following subsections, as Stages 1 and 2 were largely maintained from UC1. RM and MO (PhD and full professor of decision analysis and engineering) designed the questionnaire (Stage 3) and RM facilitated the CDB workshop (Stage 4). As UC2 is part of a broader project – developing a multidimensional management dashboard with an embedded multicriteria value model –, KPI names and descriptions were adjusted in earlier project phases. These modifications are not detailed here.

#### Stage 3 implementation and findings

To facilitate sets’ appraisal, eleven Qlik Sense^®^ pages were created. Alternative pages were designed for *Case-mix* (2 pages), *Access* (2), and *Clinical aspects* (3), based on UC1-selected formats. Four additional pages covered *Acceptability*’s (composite) KPIs, each featuring four DataViz options: (A) *Bar Chart*, (B) *Pie Chart*, (C) *Grouped Bar Chart*, and (D) a multi-KPI format. An online questionnaire (available in Portuguese as Supplementary File 2) was launched on April 24, 2024, aiming to gather individual user preferences. Four complete responses and one partial submission were retrieved.

The questionnaire had two parts: Part 1 validated UC1 choices for *Case-mix*, *Access*, and *Clinical aspects*, and Part 2 focused on *Acceptability* KPIs. Hosted on Menti™, the questionnaire took 15–20 min to complete. Respondents analyzed dashboard pages directly on Menti™ through Qlik Sense^®^ screenshots. *Target* and *Minimally acceptable* performance levels guided KPI interpretation and assessment of the DataViz format’s effectiveness in conveying relevant information. If respondents found proposed DataViz alternatives unsuitable, they could vote “Other” and suggest improvements in the comments.

Voting results and comments are detailed in Supplementary File 2. Regarding Part 1, *Access* and *Clinical aspects* had one option gathering four votes, while *Case-mix* had a 3-to-2 split favoring option B. Regarding Part 2, respondents (*a*) preferred individual DataViz for KPIs under *Patient adherence to the program* (i.e., *Dropout rate*, *Compliance with biosignal transfer* and *Medication/therapy adherence*), (*b*) split between individual and combined DataViz for *Stakeholder satisfaction* KPIs (i.e., *Patient satisfaction*, *Caregiver overload* and *Health professional satisfaction*), and (*c*) preferred combined DataViz for *Disease management capacity* and *Level of self-care*. Lastly, for *Patient’s trust in the program* (no combined option), votes split between *Bar Chart* and *Histogram*.

#### Stage 4 implementation and findings

The CDB workshop was held at HSM in Lisbon, Portugal, on May 31, 2024, with three HSM cardiologists (the remaining two stakeholders reported overlapping clinical commitments), lasting 1h38. Room and screens arrangement for in-person modality followed the same setup from UC1. Participants provided signed informed consent for participation.

At the workshop start, participants reviewed aggregated questionnaire results and justification comments on the first dashboard pages (i.e., *Case-mix* alternatives). They then discussed the (dis)advantages, constraints, and interpretations of alternative pages, reaching a consensus decision instead of formally and anonymously voting. This process was repeated for each dashboard page or (composite) KPI.

Ten different formats were used to deploy 36 KPI visuals, with 38.8% being *Bar* or *Pie Charts*. A new *Access* page visualization was requested by participants to emphasize *DAL*, *LoS*, and *Time to MD*. *Clinical aspects* KPIs were adjusted based on participants feedback. New visuals were created for *Dropout rate* and *Medication/therapy adherence*. *Level of self-care* was removed from the *Acceptability* KPIs as redundant given *Disease management capacity*, which is measured by the self-efficacy component of the Kansas City Cardiomyopathy Questionnaire (KCCQ [Self-eff.]). Table 5 presents the final list of 8 *Case-mix* variables and 25 KPIs to be included in the dashboard page prototypes.

**Table 5:** Key performance indicators’ data vizualization, description, measure(s), and reference values

KPI [DataViz]	Description	Measure	Min. Acc.	Target
**Case-mix parameters**				
Age group [Pie Chart]	Categorization of individuals based on their age range for demographic analysis and care planning.	% Patients per Age group (5 classes)	-	-
Classification of HF according to LVEF [Donut Chart]	Categorization of heart failure (HF) based on LVEF. Three classes included: HF with reduced EF, HF with mildly reduced EF, and HF with preserved EF.	% Patients per Class (HFrEF, HFmrEF, HFpEF)	-	-
Comorbidities [Bar Chart]	Knowledge of additional ailments occurring alongside the program’s primary condition.	% Patients with Comorbidity	-	-
Distance to the nearest healthcare facility [Pie Chart]	Distance, in kilometres, from the patient’s residence to the program’s base institution.	% Patients per Distance group (3 classes)	-	-
Frailty index [Donut Chart]	Patient classification according to the proportion of presented deficits out of the total age-related health variables considered.	% Patients per Class (4 classes)	-	-
Literacy level [Pie Chart]	Individuals’ ability to read, write, comprehend basic information and use digital tools, according to the Digital Health Technology Literacy Assessment Questionnaire (DHTL-AQ).	% Patients per DHTL-7AQ level	-	-
Medication/therapy [Bar Chart]	Percentage of patients receiving a certain HF medication or therapy.	% Patients with Medication/therapy		
NYHA classification [Donut Chart]	The New York Heart Association (NYHA) categorization of HF severity based on functional limitations, symptoms and the physician’s objective assessment.	% Patients per Class (I, II, III, IV)	-	-
**Access**				
Eligible patients followed by the RPM program (%) [Bar Chart]	Percentage of eligible HF patients from the healthcare institution who are enrolled in the telemonitoring program.	Ratio between TM-enrolled and hospital HF patients	-	-
HF-related length of stay [Bar Chart]	Average length of stay (LoS), in days, due to HF-related causes before discharge or death.	Avg. stay days per hospitalization	14.5	5.6
Number of days of activity lost [Bar Chart]	Total days of absence or reduced activity due to health-related needs (e.g., emergency room admission, hospitalization, premature death).	Avg. sum of LoS, emergency visits and premature death per year	48.8	5.6
HF-related hospital activity [Grouped Bar Chart]	Total count of healthcare events (ER, hospitalization, inpatient admissions) due to HF-related causes.	# Emergency visits per year	49	33
		# Hospitalizations per year	45 (36%)	15 (12%)
		# (Re)admissions per year	45 (36%)	20 (16%)
Number of consultations [Waterfall Chart]	Total count of appointments (in-person or virtual) within the program duration.	# Consultations per year	1000 (8 p.p.)	500 (4 p.p.)
Waiting time [Grouped Bar Chart]	Time from request (alert or appointment) to initiation of contact (nurse phone call, medical action, or face-to-face consultation. Telephone and MD action times in hours; face-to-face in days.	Avg. time to telephone contact	6 h	1 h
		Avg. time to medical action	8 h	3 h
		Avg. time to consultation	30 days	7 days
Time to medical action [Bar Chart]	Time, in hours and by alert severity, from an alert of patient decompensation to appropriate medical intervention and monitoring.	Avg. time for green alert	TBD	4 h
		Avg. time for yellow alert	TBD	2 h
		Avg. time for red alert	TBD	10 min.
**Clinical aspects**				
Avoidable hospital admissions due to HF [Area Chart]	Percentage of preventable (i.e., clinical alert is not responded to) hospital admissions related to HF within the program duration.	% Admissions w/ unresponded clinical alerts within 24 h	33%	0%
Biosignals [Bar Chart]	Average monthly count of clinical alerts i.e., when a patient exhibits two or more vital sign measurements outside their defined normal range.	# Clinical alerts per month	170	113
HF-related/All-cause mortality ratio [Area Chart]	Ratio between the number of deaths directly linked to HF and all occurring deaths within the program duration.	Ratio between HF and all-cause mortality	33%	10%
Number of alerts generated and severity of alerts [Grouped Bar Chart]	Count and severity of alerts related to patient deterioration or decompensation for timely intervention and monitoring.	# Alerts per alert severity (green, yellow, red) per year	-	-
Level of physical activity [Histogram]	Measurement of patients’ physical activity levels through the Six Minute Walk Test (6MWT).	6MWT score	316 (50%)	417 (75%)
Patients with ΔNT-ProBNP < + 30% (%) [Linear Gauge]	Percentage of HF patients exhibiting a decrease or less than 30% increase in N-terminal pro-B-type natriuretic peptide (NT-proBNP) levels from the baseline.	% Patients w/ NT-ProBNP decrease or increase by less than 30%	50%	75%
Mental health self-perception [Histogram]	Measurement of patients’ perception of their mental health status through the Hospital Anxiety and Depression Scale (HADS).	HADS score	11 to 21 (50%)	0 to 7 (75%)
HF symptoms self-perception [Histogram]	Measurement of patients’ perception of HF symptoms e.g., oedema, through the KCCQ (Symp.) scale.	KCCQ (Symp.) score	67 (50%)	89 (75%)
Quality of life self-perception [Histogram]	Measurement of patients’ perception of overall well-being and ability to participate in or enjoy everyday life moments through the Kansas City Cardiomyopathy Questionnaire (KCCQ).	KCCQ score	60 (50%)	77 (75%)
**Acceptability**				
Compliance with biosignal transfer [Pie Chart]	Measurement of patient adherence to transmit at least 75% of scheduled biosignal measurements, as recommended by the care team.	% Patients w/ a ratio between performed and scheduled transfers > 75%	67%	88%
Stakeholder satisfaction [Combined Line Chart]	Combined measurement of patient and health professional (HPs) satisfaction and caregiver (CGs) overload within the telemonitoring program. Patients: Home Monitoring Acceptance and Satisfaction Questionnaire (HoMASQ); HPs: Job Satisfaction Survey (JSS); CGs: 4-item Zarit Burden Interview (ZBI-4).	% Patients w/ HoMASQ score > 40.8 (Range: 0–48)	50% (24.0)	75% (40.8)
		% HPs w/ JSS class = “Satisfied” (i.e., Score > 144)	50% (108)	75% (144)
		% Caregivers w/ > ZBI-4 class = “Little or no” (i.e., Score < 21)	50% (41)	75% (21)
Disease management capacity [Bar Chart]	Measurement of patients’ ability to understand how to prevent and manage HF exacerbations through the KCCQ (Self-eff.) scale.	% Patients w/ KCCQ (Self-eff.) score > 90 (Range: 0-100)	50% (75.0)	75% (90.0)
Patient’s trust in the program [Bar Chart]	Measurement of patients’ confidence and belief in the effectiveness and reliability of the program through Dugan’s trust scale.	% Patients w/ DTS score > 20 (Range: 5–25)	50% (14.97)	75% (20.0)
Medication/therapy adherence [Grouped Bar Chart]	Measurement of patient adherence to medication intake and therapy through the Portuguese version of the Morisky Medication Adherence Scale (MMAS).	% Patients w/ MMAS class = “High” (i.e., Score = 8)	50% (6)	75% (8)
Dropout rate [Pie Chart]	Percentage of patients who abandoned the program before completion. Patients are considered dropouts if they transmit biosignals fewer than one day per week on average.	% Patients w/ a ratio between performed and scheduled transfers < 14.3% (considering everyday transfers)	8%	0%
**Costs**				
Program cost [Waterfall Chart AND Stacked Area Chart]	Total program cost, including telemonitoring elements (i.e., setup, devices) and activities, external consultations, emergency service admissions and hospitalizations.	Sum of costs of TM elements and activities, consultations, emergency visits and hospitalizations	TBD	TBD
Program cost per patient [Waterfall Chart AND Stacked Area Chart]	Total cost (on average) associated with a patient’s clinical pathway within one year of program duration. Cost components: telemonitoring (TM) elements; telemonitoring activities (Ongoing); external consultations (Cons.); emergency service admissions (ER); hospitalizations (Hosp.).	Avg. sum of costs of TM elements and activities, consultations, emergency visits and hospitalizations per year	TBD	TBD
Costs for the patient [Waterfall Chart AND Stacked Area Chart]	Total expenses (on average) directly incurred by the patient within one year of program duration. Cost components: visit; transportation; lost income.	Avg. sum of visit, transportation and lost income costs per year	288€	0€

**Fig. 6:**
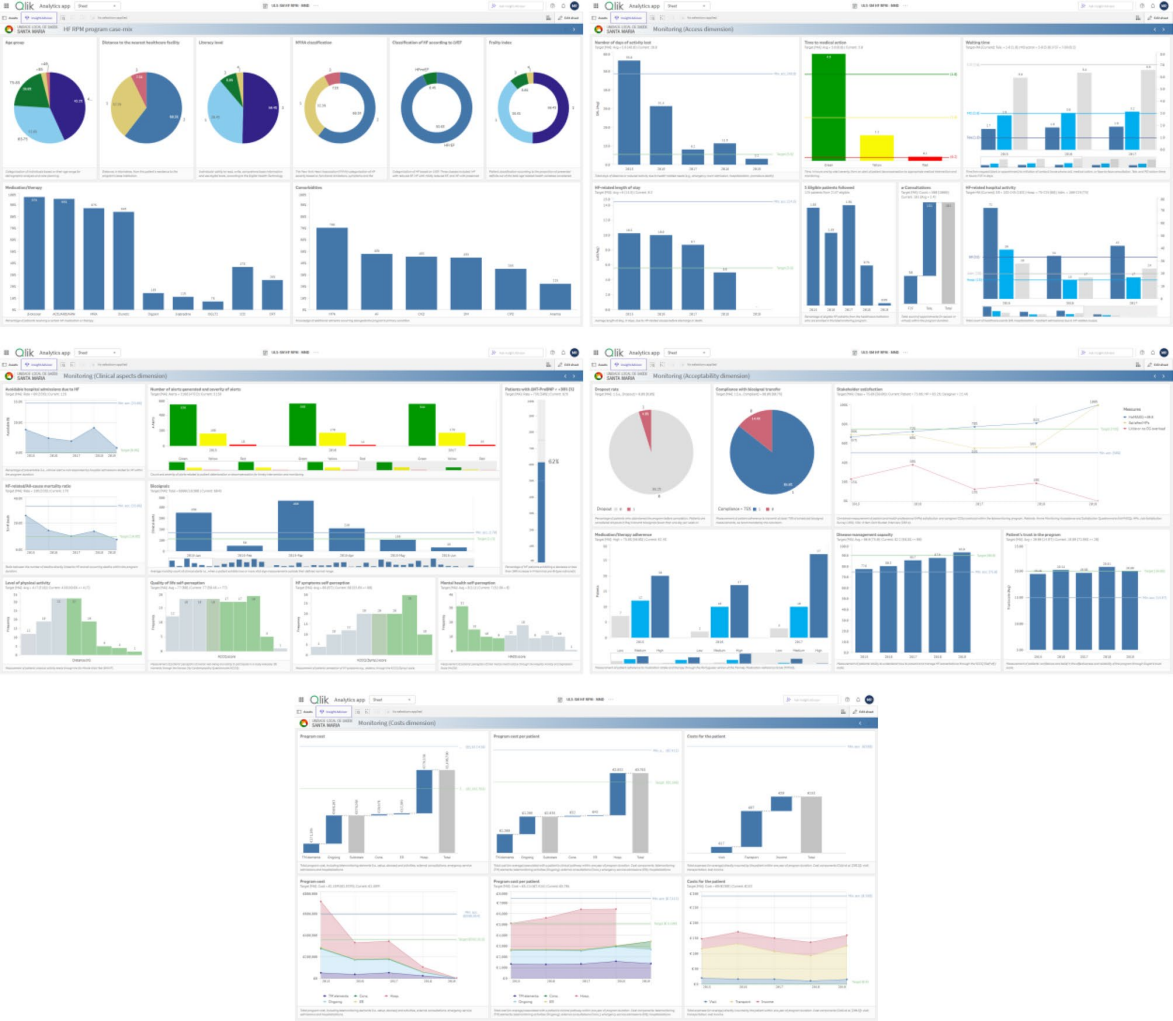
Prototype dashboard report pages for *Case-mix* (top-left) and *Access* (top-right), *Clinical aspects* (middle-left), *Acceptability* (middle-right) and *Costs* (bottom)

Regarding aesthetic and analytical features, participants requested (*a*) a usual care benchmark for *Costs*, (*b*) maintaining a blue color scheme over HSM’s palette for improved readability and neutrality, (*c*) date filters for time-dependent analysis and (*d*) a drill-down feature for *Patient’s trust in the program*, enabling a switch between yearly averages and histogram. Figure 6 displays the finalized prototypes for *Case-mix*, *Access*, *Clinical aspects*, *Acceptability* and *Costs*.

## Discussion

### General considerations

Unlike traditional technocentric design, our CDB approach actively engages users in a constructive, collaborative and human-centric process led by experienced facilitators, enhancing software acceptance and adoption and driving resilient innovation in healthcare management dashboards [64]. By making users co-developers, systems better meet stakeholders’ diverse needs and users gain a deeper understanding of the technology, effectively opening the “black box.” This perspective aligns with recommendations by Sommer et al. [3], emphasizing the role of early stakeholder involvement in technology implementation as an enhancer of usability, confidence, and technology integration within healthcare workflows. Moreover, we consider that our study answers the authors’ call for more human-computer interaction research in assistive health technology.

Despite the potential for lengthy CDB workshops due to iterative analysis, voting, and group discussions for each KPI, engagement remained high. In UC1, focusing on prototyping dashboard pages for *Access*, *Clinical aspects*, and *Case-mix*, participants completed the workshop in 2.5 h, with the group quickly adapting to the voting platform protocol and to the employed DataViz formats. In UC2, as scheduling a 2.5-hour session for all five HSM cardiologists was impractical due to medical duties and scheduling conflicts, an online questionnaire enabled individual KPI assessments at their convenience. Conducting the first voting round online shortened the CDB workshop by an hour (38%) while covering more dimensions and KPIs than UC1 (5 dimensions, 36 visuals vs. 3 dimensions, 19 visuals). However, the questionnaire was open for a month, requiring repeated reminders and deadline extensions, a common issue in similar studies [41, 65, 66].

Reported application times compare well with similar health-related and/or dashboard-building projects. Maguire et al. [51] note that “the total time of the NGT was 4 h including scheduled breaks;” while Lau et al. [43] report that “dashboard development time is highly variable, ranging between 3 and 6 months.” The CDB workshop, however, enables rapid and structured onsite dashboard prototyping during short timeouts. Unlike technocentric methods requiring weeks or months of iteration [26], our approach allows stakeholders to collaborate in a session, producing dashboard pages integrating selected DataViz formats and delivering direct feedback to developers in real-time. Although our cases report shorter durations, direct efficiency comparisons are limited by variations in engagement, group size, scheduling, facilitation, context and nature of tasks.

Furthermore, while UC1 and UC2 focus on HF telemonitoring within Portugal, the proposed approach is adaptable to other telemonitoring contexts (e.g., oncology, mental health, post-operative care), broader healthcare applications (e.g., emergency services, public health), and even non-healthcare fields (e.g., vehicle dashboard design). Such flexibility stems from the methods’ emphasis on collaboration and idea exchange during DataViz design, across synchronous or asynchronous, online or in-person settings. Thus, broader applicability is more a matter of engaging relevant stakeholders and end-users in meaningful discussions, starting from validated and operational KPIs and guided by experienced facilitators (who employ the appropriate resources to guide the CDB process), whether or not they are domain experts – as stakeholders own the problem and the solution [60].

### Implications for healthcare management and decision-making

Our study contributes to healthcare management and decision-making literature in two primary aspects. First, it addresses the lack of insightful and actionable information within data-rich healthcare environments – we detail the collaborative building process of tailored and user-friendly dashboard pages to support HF telemonitoring program management. While both UC1 and UC2 began with an exhaustive set of KPIs collaboratively developed by about 30 Portuguese HF telemonitoring experts [34], the CDB process still allowed for refinement (e.g., removing irrelevant KPIs, adding context-specific ones, or changing KPI names and descriptions), illustrating the dynamic nature of healthcare data and performance management [67]. The CDB approach also translates these abstract performance concepts into visual, intuitive and interactive information. Figure 6 outlines five pages for program monitoring and evaluation, aligned with program goals i.e., to ensure timely patient access while reducing hospital stays, promote clinical excellence, and guarantee high satisfaction and adherence, while aiming for the most value for money – core aspects of a value-based healthcare approach [68]. Each page integrates agreed-upon DataViz formats for KPIs within its focus area, totalling 36 visuals (average 7.2 per page), with the *Clinical aspects* page comprising a maximum nine visuals and *Acceptability* and *Costs* a minimum six – to minimize cognitive overload, visual elements were limited per page [6], guided by the “magical number seven, plus or minus two” principle [69]. Contextual elements like titles, footnotes and legends support KPI interpretation. Interactive filters allow year-specific analysis across KPIs. Reference lines (for *Target* and *Minimally acceptable* performances) help users assess the current performance against the agreed-upon program objectives.

Second, the study addresses the limited stakeholder engagement in healthcare dashboard design by promoting collaboration. All KPI measures, visuals, reference levels, and interactive features were collaboratively defined by users, ensuring the dashboards are relevant, intuitive, and fully adopted. In practice, the proposed approach helped tailor a predefined KPI list [34] to the unique protocols and management needs of two HF telemonitoring programs, where stakeholder input proved fundamental [23, 25]. For instance, both UC1 and UC2 include visualizations for *LoS* and *Number of consultations* KPIs; but their implementations differ significantly. In UC1, *LoS* was broke down into ward and ICU stays, reported monthly, while, in UC2, an aggregated annual figure is reported. In UC1, also a distinction is made between scheduled/unscheduled and face-to-face/teleconsultations; UC2 only differentiates face-to-face and teleconsultations. These differences reflect each program’s needs and protocols. Regarding *LoS*, UC1, launched during the pandemic and still in its early phase, required more detailed metrics to support consolidation and understand the patient journey; UC2, more established (in its sixth year), benefited from more aggregated data. Regarding consultations, in UC1, both scheduled visits and emergency consultations are considered in the protocol, while UC2 only accounts for unscheduled consultations – making the scheduled/unscheduled breakdown irrelevant. These examples illustrate that while it is beneficial for similar health services in different organizations to monitor a common KPI set (e.g., aiding benchmarking), implementation must be tailored to each program. Stakeholder engagement is therefore essential to build dashboards that truly support local needs.

### Insights on collaboration and group dynamics

In UC1, the CDB workshop, originally planned for exclusive onsite participation, shifted to a hybrid modality as two attendees could only participate remotely. This adjustment introduced technical challenges, particularly with Microsoft Teams™ screen-sharing and engaging online participants. Facilitators initially struggled to ensure equal and consistent access to information (e.g., Qlik Sense^®^ or Menti™ screens) and balance participation in fast-paced discussions. The experience highlights the impact of workshop modality on session dynamics and, potentially, approach effectiveness. Predefining the workshop format and adequate and comprehensive preparation are essential, as last-minute adjustments can lead to unforeseen constraints.

Iterative voting, akin to other modified NGT applications [48, 54], was integral to our proposed approach. For UC1 and UC2, the protocol involved an initial vote (via an online questionnaire in UC2), results discussion, and a final vote (anonymous in UC1, decided by consensus in UC2). However, if the discussion confirmed agreement on the top choice, the final vote was exempted to save time and reduce the cognitive burden of another voting process. This was particularly evident in UC1 when analyzing *Clinical aspects* KPIs, where similar KPI communication properties led to consistent format choices, such as selecting a *Bar Chart* for *Mental health* and *QoL* after a single vote. If a final vote had been truly necessary, skipping it would have had little impact since format decisions could still be adjusted during prototype reviews. However, when discussion did not confirm consensus, a second vote remained crucial. Strong arguments and new perspectives can shift outcomes, as seen in UC1’s *Mental health* discussion, where support moved from option A (66.7% in the first vote) to option B after persuasive reasoning. Therefore, we advocate for maintaining a minimum two sequential voting rounds as the standard protocol, with facilitators assessing each case and consulting participants before continuing a second vote.

The proposed approach mitigated group biases by using Menti™ for anonymous voting and comment sharing, ensuring all participants could express freely and truly. While anonymity was maintained, a summary enabled collective review and discussion, fostering shared knowledge. Participants responded positively to anonymous voting, yet a tendency towards open dialogue verified. Recognizing this tendency and to enhance engagement, the facilitators allowed verbal argument exchange after the first vote in both UC1 and UC2. In UC2, online questionnaire comments created a productive hybrid of anonymous and open dialogue, allowing participants to analyze feedback aloud without revealing identities. In UC1, however, open discussion introduced some initial challenges due to the presence of a participant with leadership status and peer-recognized HF expertise. To ensure balanced participation and mitigate undesirable sway, facilitators encouraged all voices to participate.

Finally, regarding the CDB workshop environment, a spacious room with a minimum two screens – one for analyzing DataViz options and another for displaying voting results – is deemed essential to support an extensive and informed group discussion. In a hybrid format, a third monitor may be needed for onsite-online interaction. Workshop preparation also includes selecting a dashboard-building software (for preparing visualization alternatives and for onsite report development) and an online voting platform. Menti™ enabled free browser access through any internet-connected device with a session code, eliminating the need for a mobile application download.

### Limitations of the study

Although positive feedback in UC1’s post-workshop survey and verbal praise from UC2 stakeholders suggest our approach was successful, this study is not without limitations. First, while conducting two cases helped test the transferability of methods and findings across settings, the relatively small number of involved stakeholders (six in UC1 and five in UC2) may limit generalizability. In both cases, the HF telemonitoring program coordinator invited all stakeholders and potential users considered relevant based on the local and department-specific nature of the developed dashboards. Thus, a limited number of participants was expected – and even preferred by coordinators. Notwithstanding, UCs engaged, collectively, a diverse range of individuals involved in healthcare and technology provision and management, capturing both clinical- and operations-focused views. UC groups also varied in composition – UC1 with a heterogeneous group, while UC2 engaged only cardiology physicians.

Second, we used synthetic data in both UC1 and UC2 to produce KPI visualizations, which may not fully capture real-world characteristics. Synthetic data was developed due to data unavailability, posing challenges in generating credible datasets for all KPIs. Despite extensive exploration of healthcare databases to mimic real-world data, there remains a lack of guidance on effectively generating synthetic data and accurately representing each KPI. To support future integration with real-world data, extensive validation is needed e.g. verifying fact expressions (i.e., everyday language statements describing information to be retrieved), testing dashboard reporting across different time frames, patient groups, and combined visualizations, and ensuring reference levels remain appropriate. Moreover, assumptions were made to align KPI properties with end-user requirements. To address this issue, future work should incorporate a stage between identifying KPIs and determining DataViz formats wherein users collaboratively define specific measures for each KPI, ensuring KPI variables are clearly represented.

Third, there were challenges in developing the *Costs* dashboard page (from UC2), as HSM project stakeholders suggested comparing telemonitoring costs with usual care, and this required further research and was beyond the scope of the work under development. Marsh et al. [70] stress the need for comparability in cost and benefit estimation; yet UC1 and UC2 participants expressed concerns that focusing solely on program or department costs would overlook system reimbursement mechanisms or other opportunity costs. These challenges justify the exclusion of *Costs* KPIs from a detailed DataViz appraisal in both UC1 and UC2.

Fourth, several facilitation challenges emerged both in UC1 and UC2. In UC1, the main issues were managing dominant voices and allocating time efficiently, ensuring balanced participation. This was addressed through user-friendly participation DSSs and workshop co-moderation. In UC2, challenges included achieving full questionnaire responses, requiring multiple email reminders for some participants, and keeping the CDB workshop brief to accommodate participants’ clinical commitments, preventing rushed discussions.

Finally, participants’ digital literacy is key in dictating the effectiveness of workshop techniques. Combining open access tools like Microsoft Teams™, Menti™, and Qlik Sense^®^ was successful in our UCs, as participants understood the tasks involving this tools and exhibited contentment. For less digitally literate groups, however, alternative approaches may be required.

## Conclusions

This study presents a novel collaborative process that actively engages dashboard end-users in selecting the most suitable DataViz formats for a predefined KPI list, ensuring the final dashboard meets their needs. Grounded in literature, the approach combines participatory approaches with BI techniques to enhance dashboard development. To our knowledge, this is one of the first studies to involve end-users in onsite (structured) dashboard co-development, aiming to improve adoption, acceptance, and informed decision-making.

Our findings highlight the role of user participation and feedback in technology design, particularly within the context of dashboard development. Yet, future research is needed to assess the real-world effectiveness and usability of the resulting tools. Such validation should involve ongoing and iterative feedback to support design improvements while maintaining the collaborative relationship between users and developers established during the CDB process.

This collaboration effort between health professionals, scholars, industry and technology developers to build and test new management tools leveraging clinical, operational, societal, and financial data aligns with the principles of Society 5.0 – a data-driven society where technocentric and human-centric perspectives converge to ensure prosperity by balancing economic growth, technological development, and social welfare [64].

## Supplementary Information

Below is the link to the electronic supplementary material.

Supplementary Material 1

Supplementary Material 2

## Data Availability

All data analyzed during this study is included in this published article. Synthetic datasets generated for dashboard visualizations during the current study are available from the corresponding author upon request.
