# Supplementary material for: From predefined indicators to a management dashboard for heart failure telemonitoring: a modified nominal group technique approach in Portuguese hospitals
Source: BMC Health Serv Res. 2026 Jan 23;26:256. doi: 10.1186/s12913-026-14031-1 (PMC12911346; doi:10.1186/s12913-026-14031-1)
Supplement: Supplementary file 1 — Supplementary Material 1 [file 12913_2026_14031_MOESM1_ESM.docx]

### UC1’s Post-Workshop Feedback Survey

Following the CDB workshop, participants received an email acknowledging their active participation along with a post-session feedback survey to assess participant acceptance of the approach and gather feedback for potential improvements. Google Forms was selected as the delivery platform due to its formatting features, accessibility, and user-friendly interface. The survey comprised seven statements related to the workshop, inviting participants to express their agreement on a five-point Likert scale, ranging from Strongly Disagree (SD) to Strongly Agree (SA). Additionally, a final comment section was included for more detailed, open-ended feedback. The statements comprised in the survey are as follows (translated to English from Portuguese):

1. I found the approach to be user-friendly in practice.
2. The workshop was effective in promoting consensus.
3. This approach helps bridge the gap between end-user preferences and dashboard developer decisions.
4. This approach is adaptable to various healthcare contexts.
5. This approach should be a standard practice when developing dashboards.
6. This approach helps ensure that developed dashboards are more effective in facilitating decision-making.
7. Overall, I am satisfied with this approach.
8. Please, share any feedback, ideas, or recommendations for enhancing the approach or its implementation.

Five out of six participants completed the questionnaire. Survey data is summarized in Figure S1. Respondents expressed satisfaction with the proposed approach, endorsing its application within dashboard development. Workshop participants found the methodology to be user-friendly and believe it can enhance dashboard effectiveness, with one participant noting that “bringing together the different users allows the development of more accurate and user-friendly solutions.”


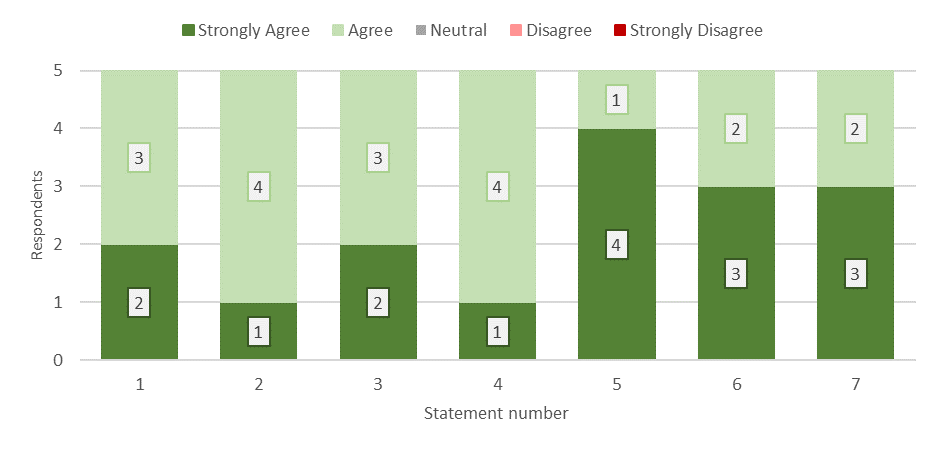


**Figure S1. Post-workshop feedback survey results.**
