# Supplementary material for: From predefined indicators to a management dashboard for heart failure telemonitoring: a modified nominal group technique approach in Portuguese hospitals
Source: BMC Health Serv Res. 2026 Jan 23;26:256. doi: 10.1186/s12913-026-14031-1 (PMC12911346; doi:10.1186/s12913-026-14031-1)
Supplement: Supplementary file 2 — Supplementary Material 2 [file 12913_2026_14031_MOESM2_ESM.pdf]

Collaborative Dashboard Building (CDB)

Acede a

www.menti.com

Introduz o código

18 27 28

Ou usa o código QR

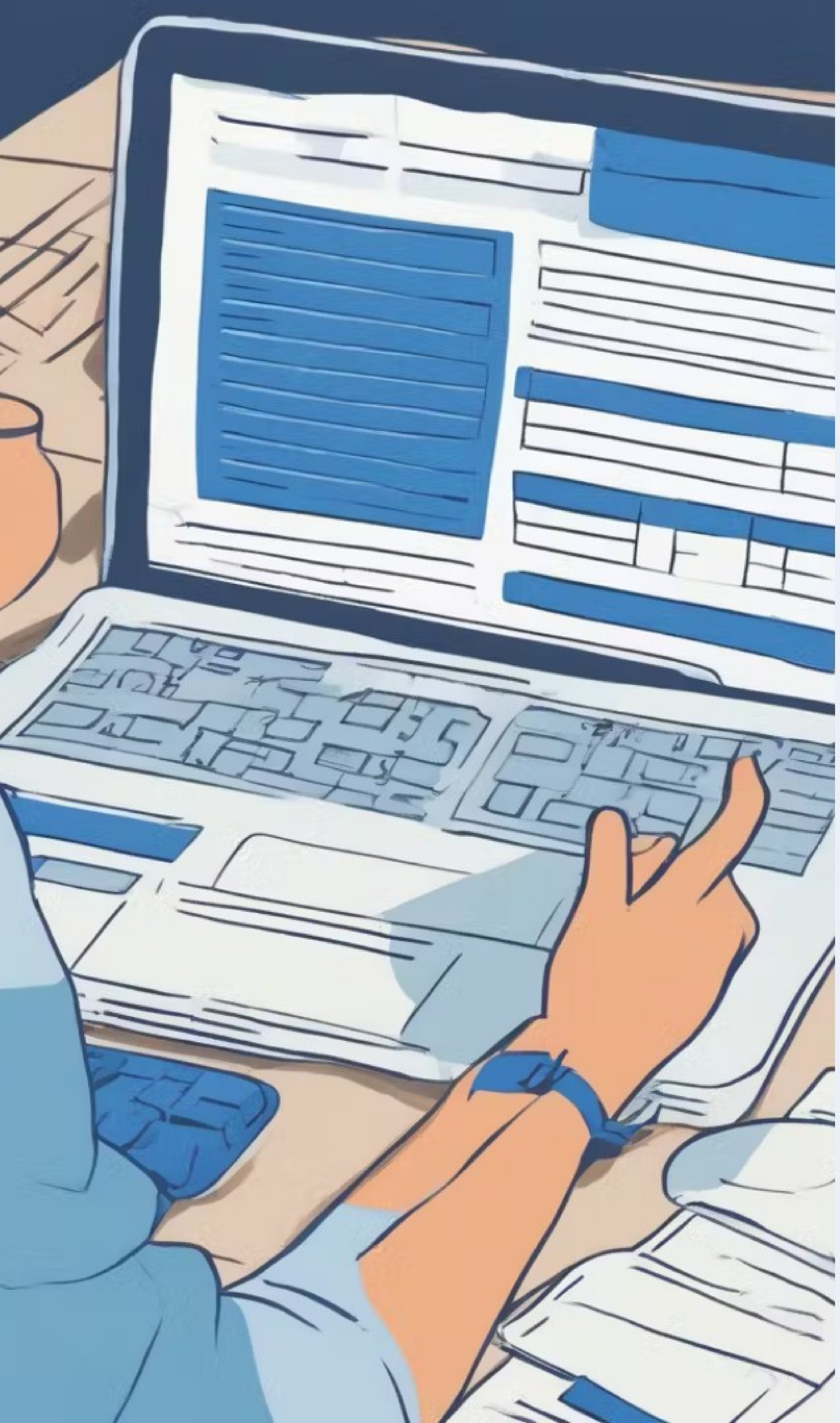

Contexto e objetivo

Mesmo com descrições claras e medidas inequívocas, cada KPI pode ser visualizado de diferentes formas e as preferências de visualização variam entre indivíduos. E, num contexto de grupo, como podemos identificar que formato recolhe a maior concordância? Esse é o objetivo deste questionário!

Para uma dimensão ou KPI de cada vez, pede-se que EXPLORE opções alternativas de visualização, VOTE na sua opção preferido e COMENTE razões que justifiquem e favoreçam a sua escolha.

No workshop CDB (não se esqueça de preencher o Doodle!), será apresentado um resumo dos resultados da votação e comentários, promovendo a discussão e, talvez, influenciando a votação subsequente. A opção de visualização com maior número de votos para cada KPI será incluída no dashboard final.

Antes de começar...

- Os dados apresentados são, predominantemente, sintéticos e não devem ser o foco da análise.
- Para uma análise detalhada, clique nas figuras e depois selecione (com o botão direito do rato) "Abrir imagem num novo separador."
- Pode votar uma opção e utilizar os comentários para sugerir alterações à proposta.
- Caso opte por votar "Outra", utilize os comentários para sugerir uma visualização alternativa.
- TARGET e MIN. ACC. são valores de referência que contextualizam a análise.
- TARGET: um "bom desempenho" realista a atingir durante o programa.
- MÍN. ACC.: o menor nível de desempenho considerado satisfatório.

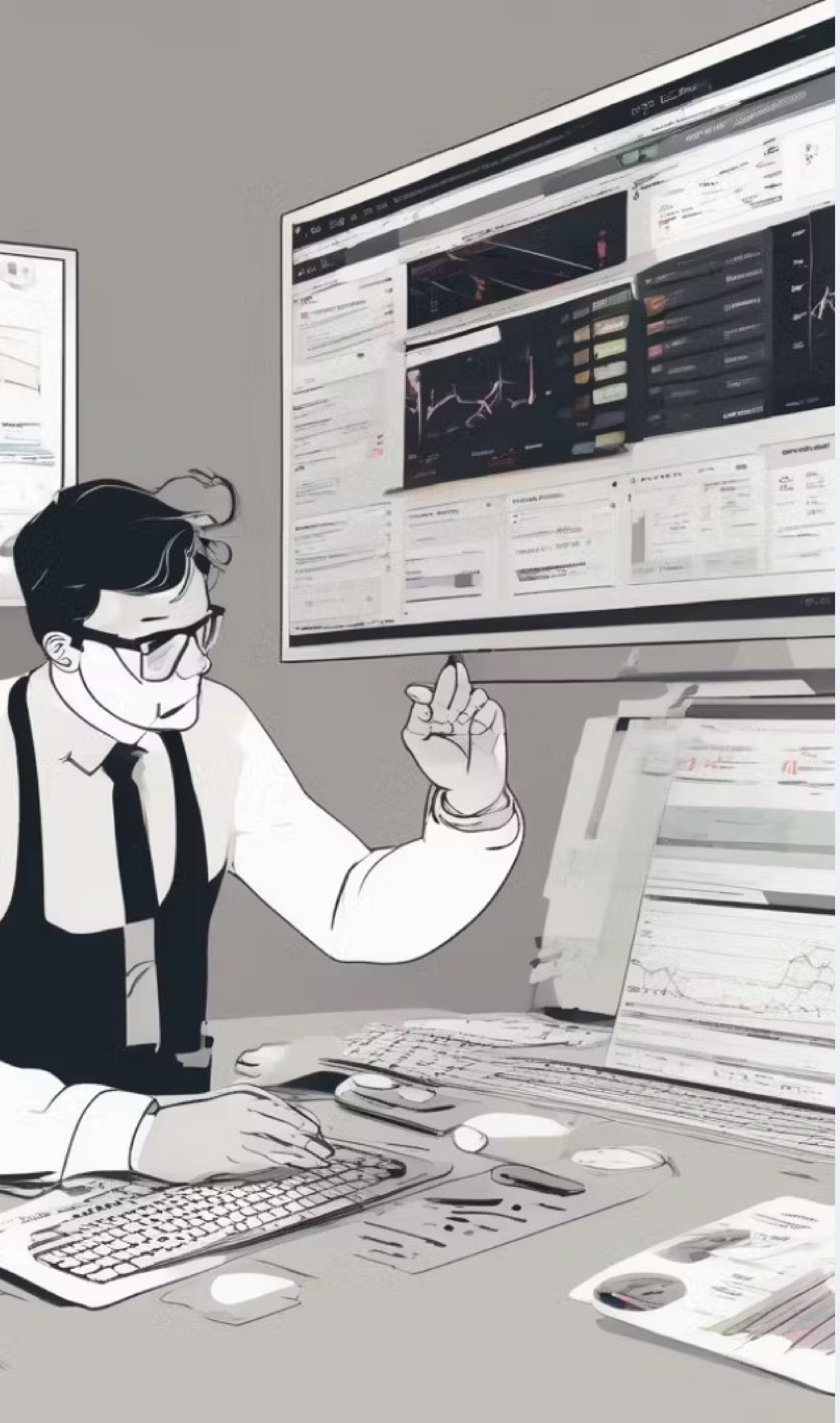

Parte 1: Protótipos já desenvolvidos

Num estudo anterior, foram desenvolvidos dashboards para três dimensões de avaliação do programa de telemonitorização: Case-mix, Acesso e Aspetos clínicos. No entanto, opções de visualização diferentes podem ser mais adequadas agora. Explore as opções de dashboard propostas e vote na sua preferida para cada dimensão.

Case-mix

Descreve a casuística e complexidade dos doentes seguidos pelo programa de telemonitorização não-invasiva de IC. Inclui variáveis relativas a idade, distância ao hospital, literacia, classificação NYHA e segundo LVEF, fragilidade, medicação/terapêutica e comorbidades.

Para a dimensão "Case-mix", escolha uma das seguintes opções...

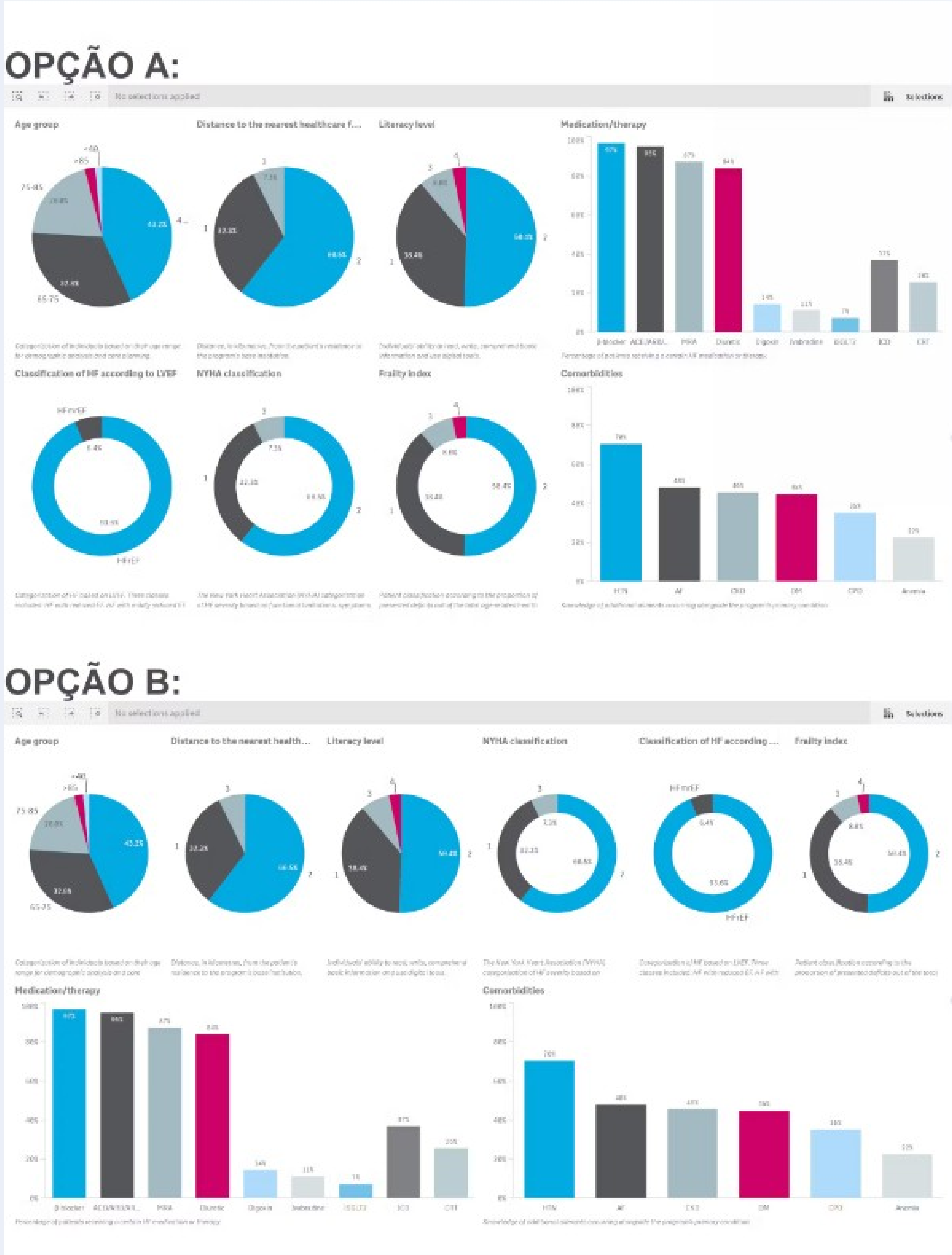

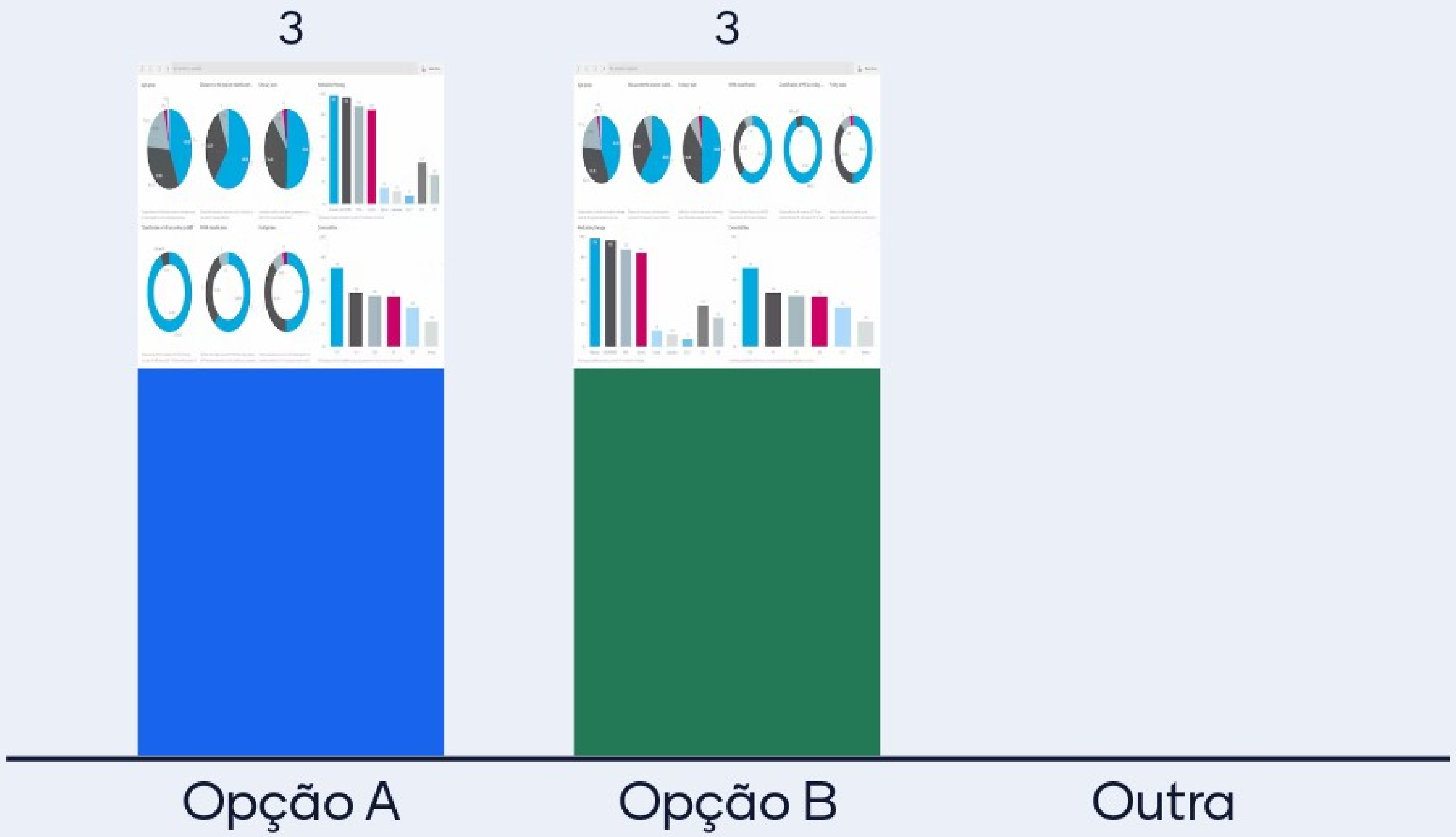

6

Comentários:

Apresentação gráfica mais simples de ler, colocando os gráficos de barras em baixo.

Mais apelativa

Visualização mais rápida aparentemente e mais chamativa

Visualização mais rápida e facil

Penso que seja mais legível visualizar a informação em quadrantes, começando pelo superior esquerdo, inferior esquerdo, superior direito e inferior direito. Daí a opção escolhida.

4

Acesso

Informa sobre a capacidade dos doentes obterem cuidados de forma equitativa, oportuna e atempada. Inclui KPIs para captação de doentes elegíveis, demora média de internamento, absentismo, atividade hospitalar e tempos de espera.

Para a dimensão "Acesso", escolha uma das seguintes opções...

OPÇÃO A:

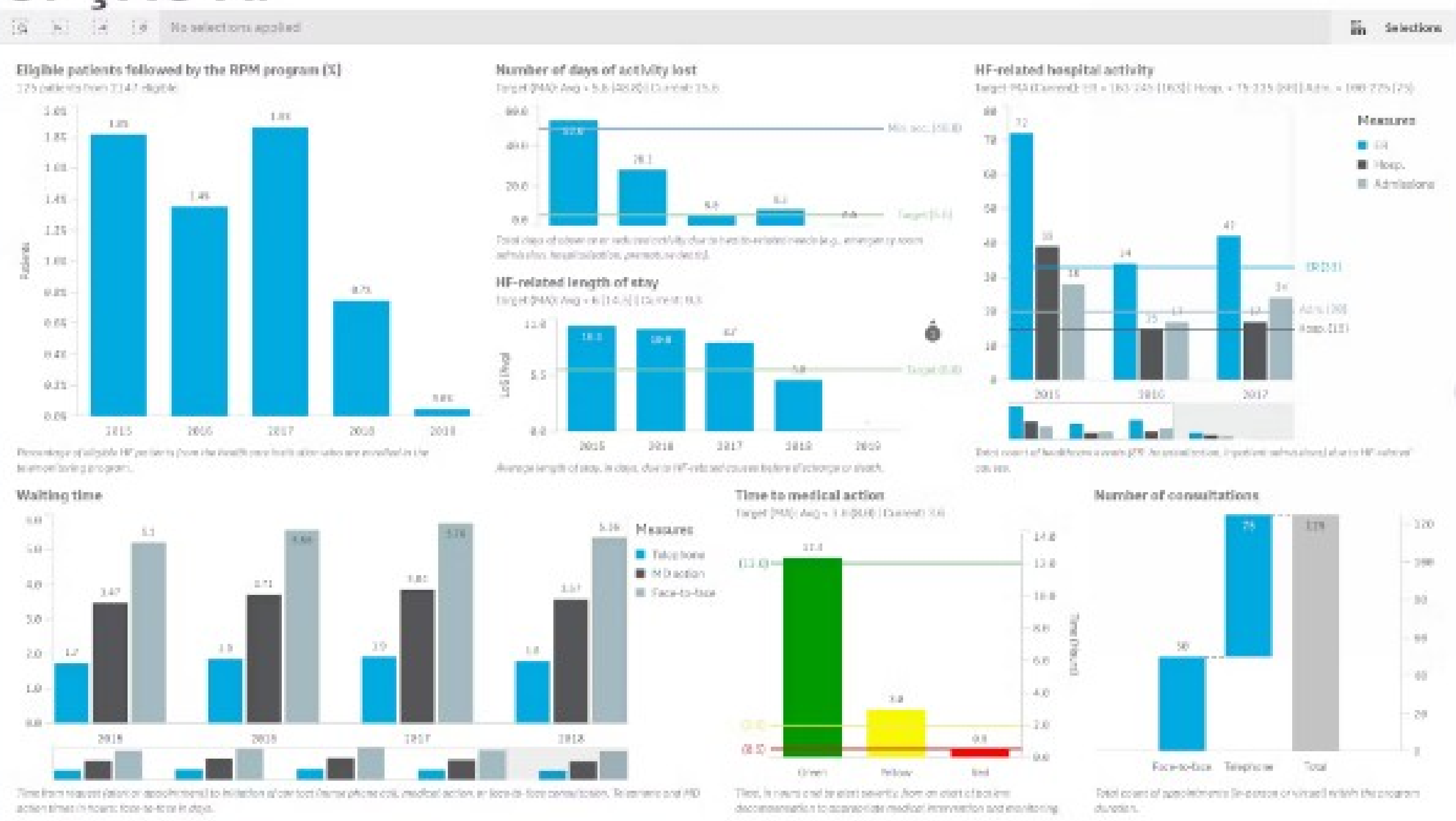

OPÇÃO B:

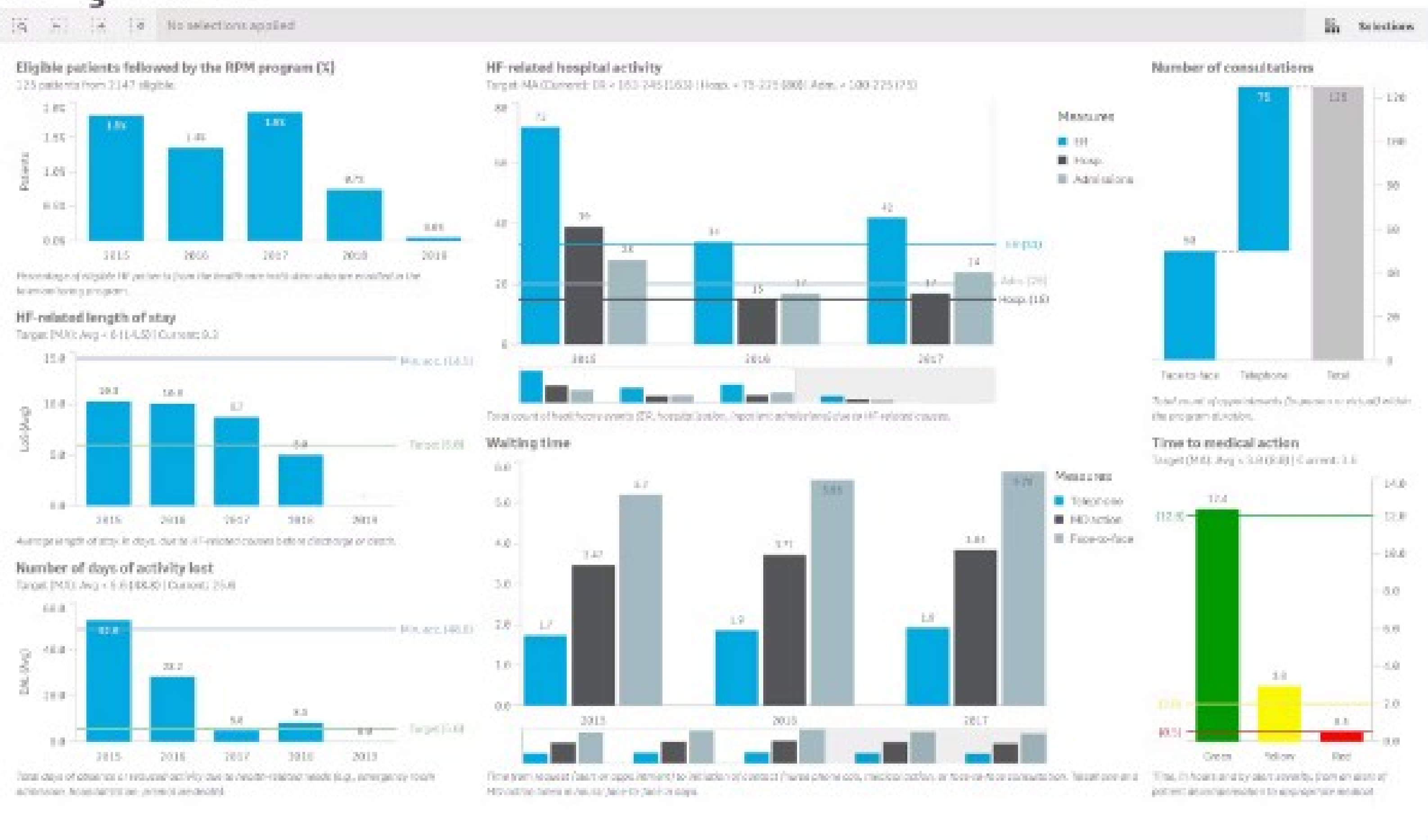

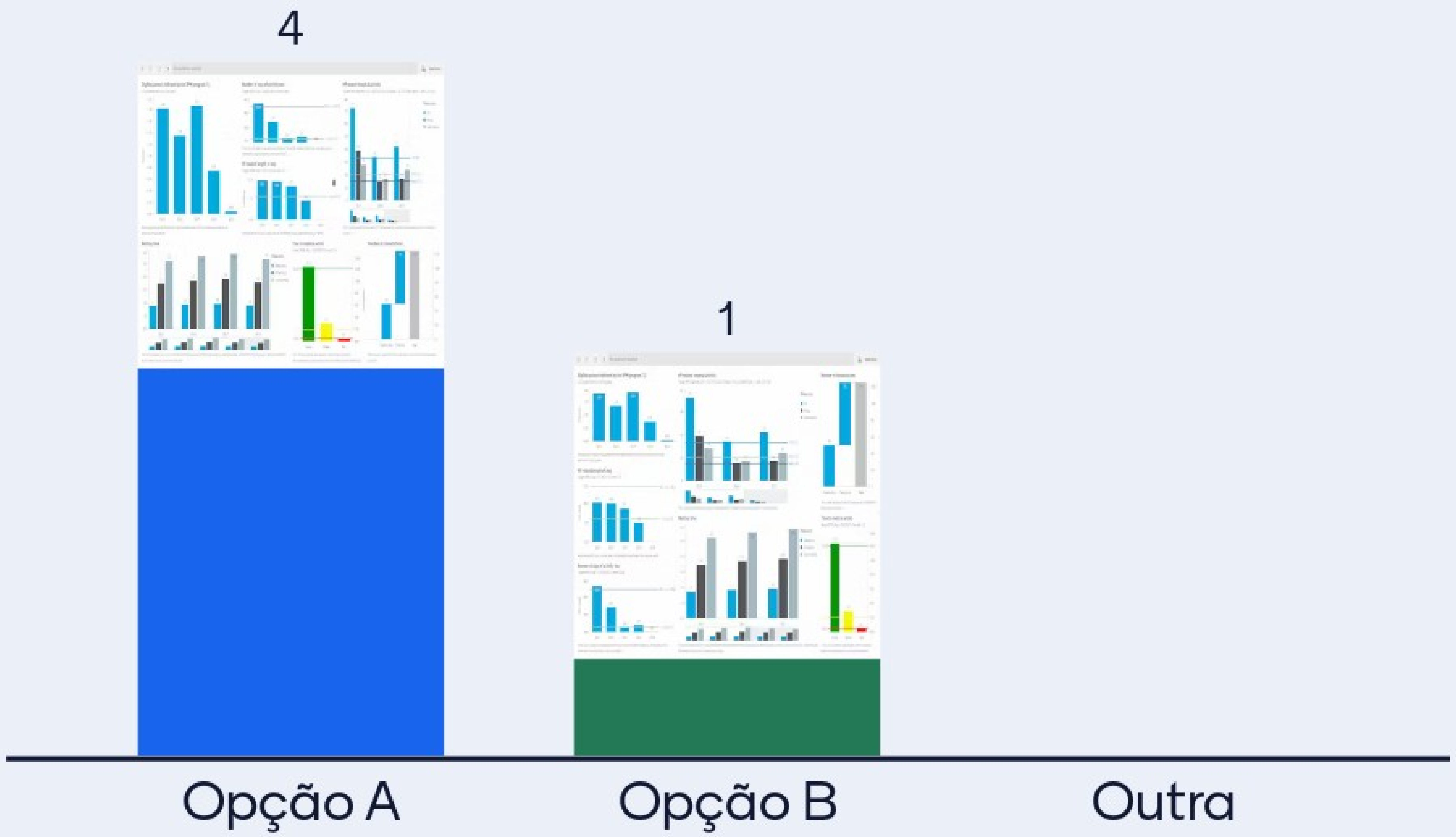

5

Comentários:

Mais fácil perceber o resultado das métricas e coloca nr of days e HF related length of stay (variáveis correlacionáveis) no mesmo "setor"

Acho que são bastante semelhantes. A B está apenas mais alinhada

Mais fácil de apreender visualmente

Os KPI mais importantes são o tempo perdido e o tempo de internamento pelo que me faz mais sentido a opção A em que estes KPIs se encontram mais legíveis.

4

Aspetos clínicos

Informa sobre os efeitos da intervenção e cuidados afetos ao programa. Inclui KPIs para admissões evitáveis, mortalidade, alertas gerados, biomarcadores da doença e PROMs.

OPÇÃO A:

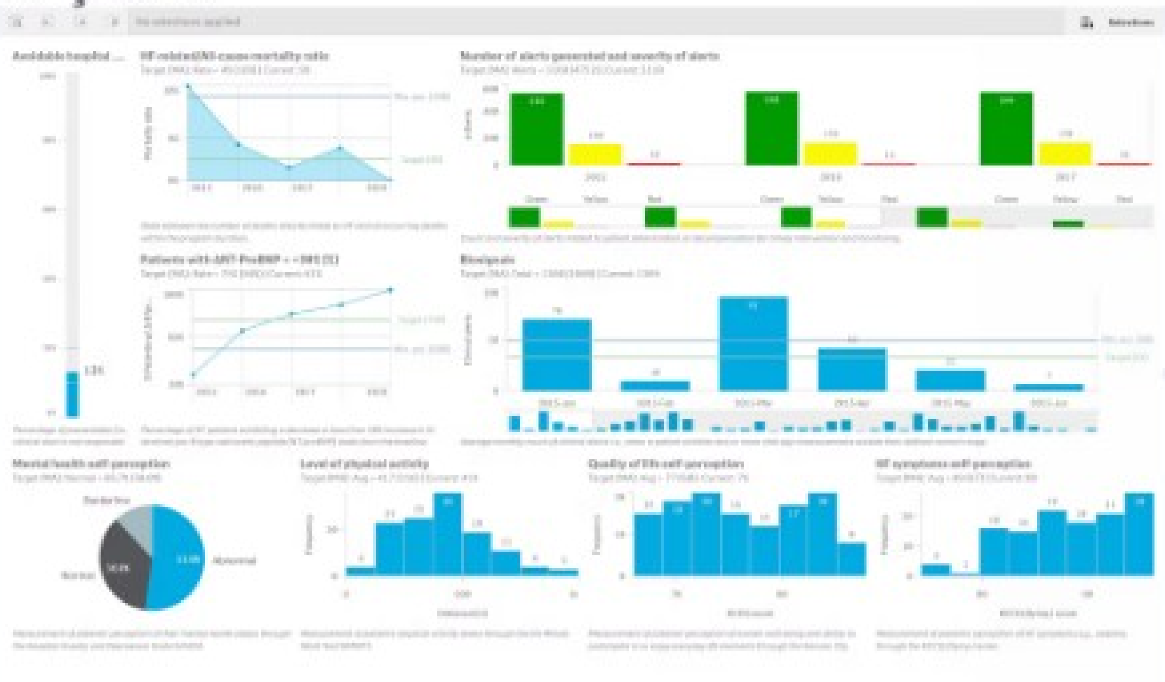

OPÇÃO B:

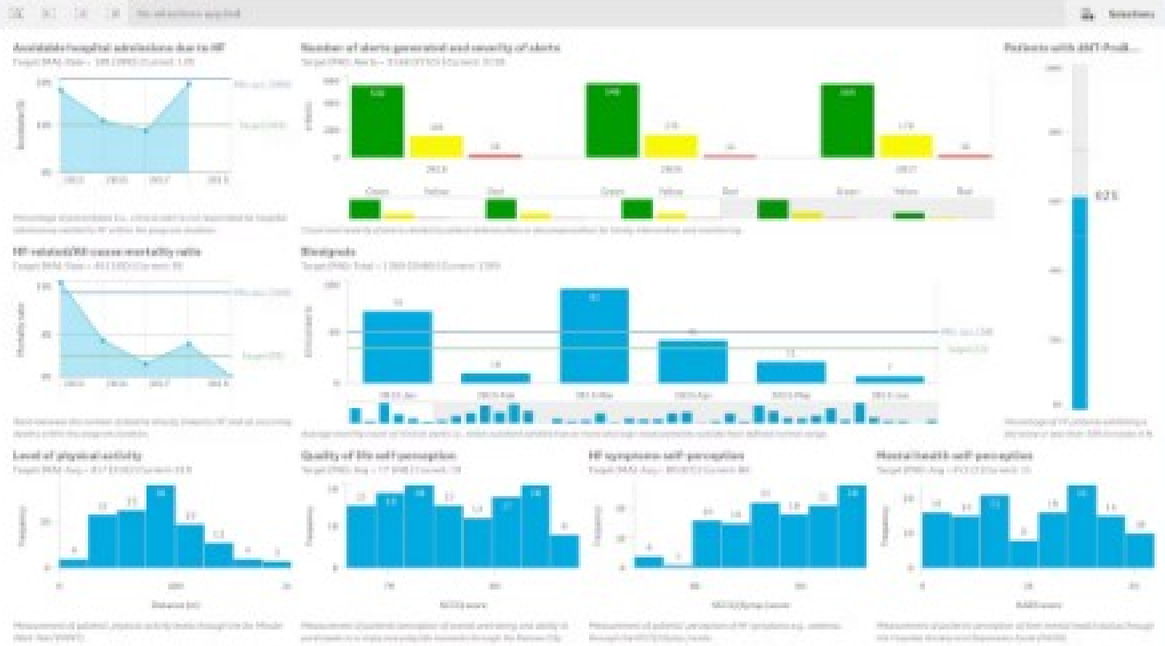

OPÇÃO C:

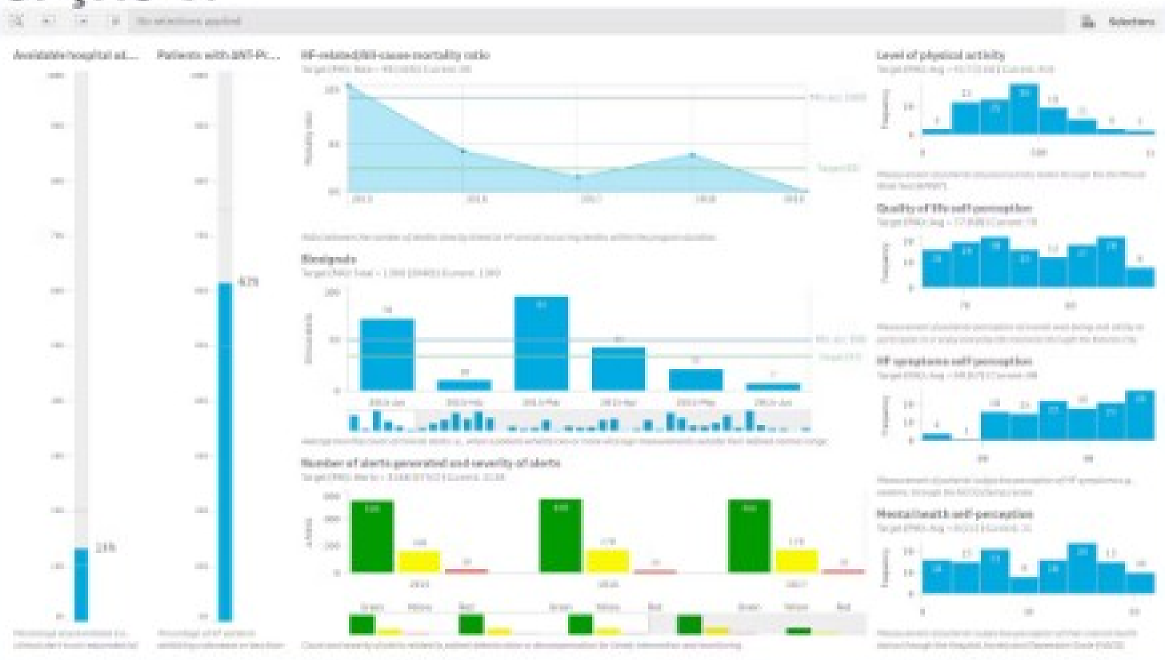

Para a dimensão "Aspetos clínicos", escolha uma das seguintes opções...

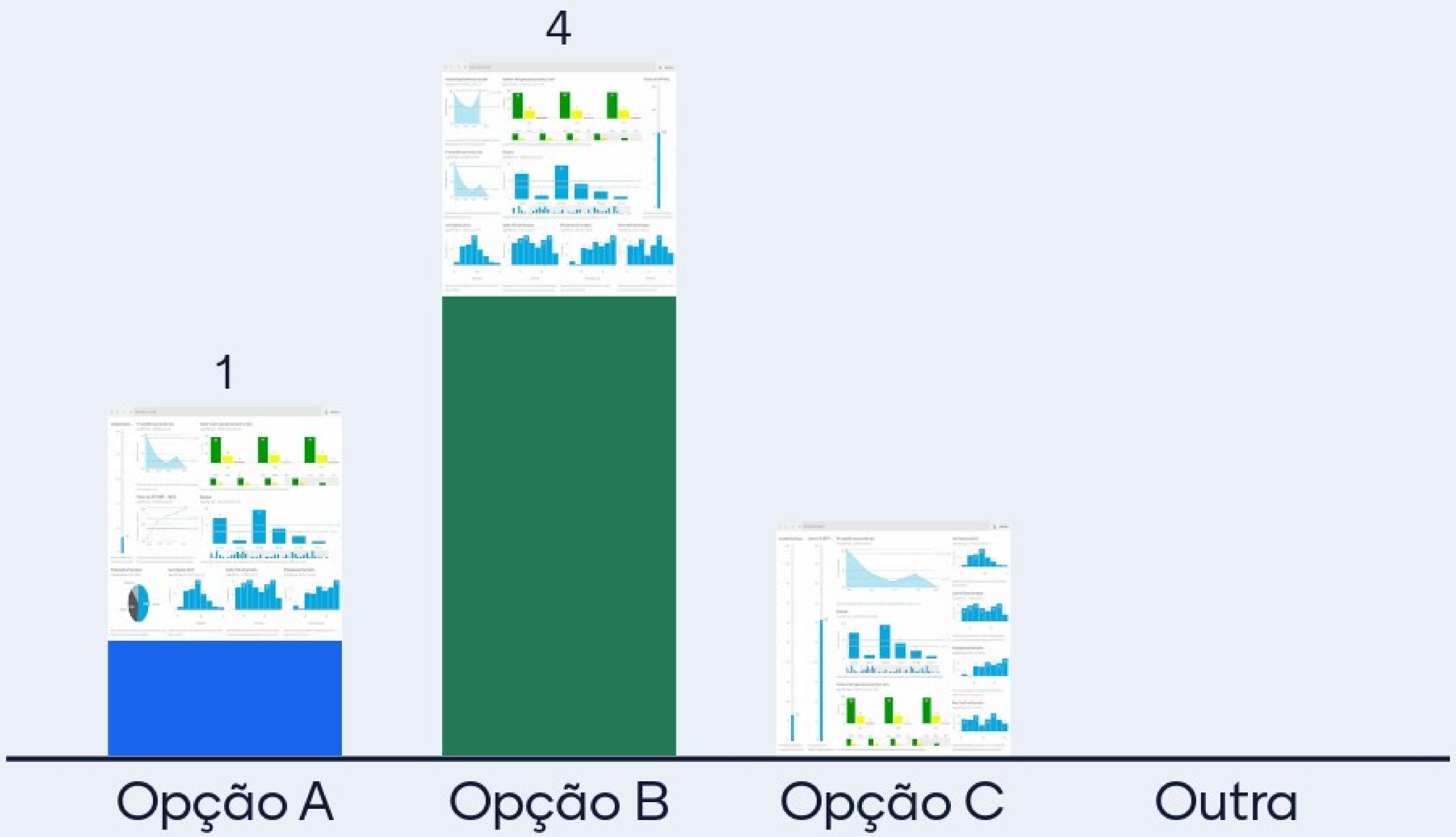

5

Comentários:

Destaca mais os internamentos evitados

Proporia que nos gráficos os extremos tivessem cores diferentes para se tornar visualmente mais perceptível (p.e. Doentes com excelente vs péssima QV)

Mais organizado e menos confuso

Na opção B torna-se mais visível as hospitalizações evitáveis e a mortalidade.

4

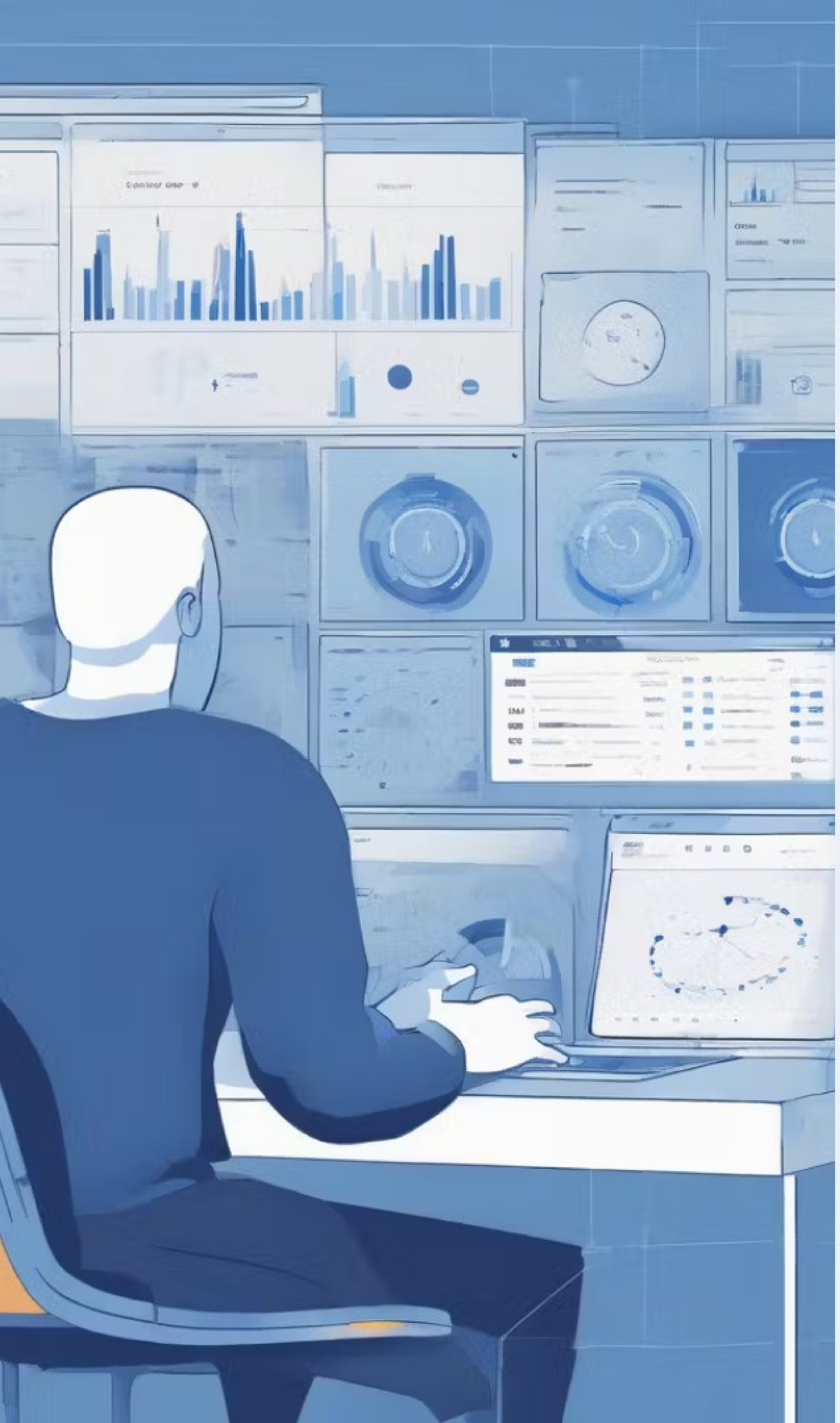

Parte 2: KPIs da dimensão "Aceitação"

Serão apresentados diferentes formatos de visualização para KPIs da dimensão "Aceitação", incluindo opções que combinam múltiplos KPIs. Explore cada grupo de KPIs, indique se prefere uma opção combinada ou individuais e, caso prefira visualizações individuais, vote no seu formato preferido para cada KPI.

Antes de começar...

- Qualquer KPI individual pode ser representado por um gráfico de barras (Opção A), em pizza (Opção B) ou em barras agrupadas (Opção C).
- As escalas de avaliação podem ser alteradas, sendo encorajadas sugestões via comentários.
- Múltiplos KPIs podem ser definidos pela (1) pontuação derivada da escala aplicada OU (2) percentagem de doentes numa dada classe de pontuação.
- Para o caso (2), os valores de referência padrão são TARGET = 66,7% e MIN. ACC. = 50% - indique alternativas em caso de discordância!

Adesão do doente ao programa

Inclui KPIs para conformidade de transferência de biossinais, adesão à terapêutica e taxa de abandono. Medidas/escalas propostas: Abandono: % de doentes que transmitem <1x por semana; Conformidade: rácio de transmissões concluídas vs. planeadas; Terapêutica: Morisky Medication Adherence Scale (MMAS).

Para os KPIs associados a "Adesão do doente ao programa", prefere visualizações individuais ou combinada?

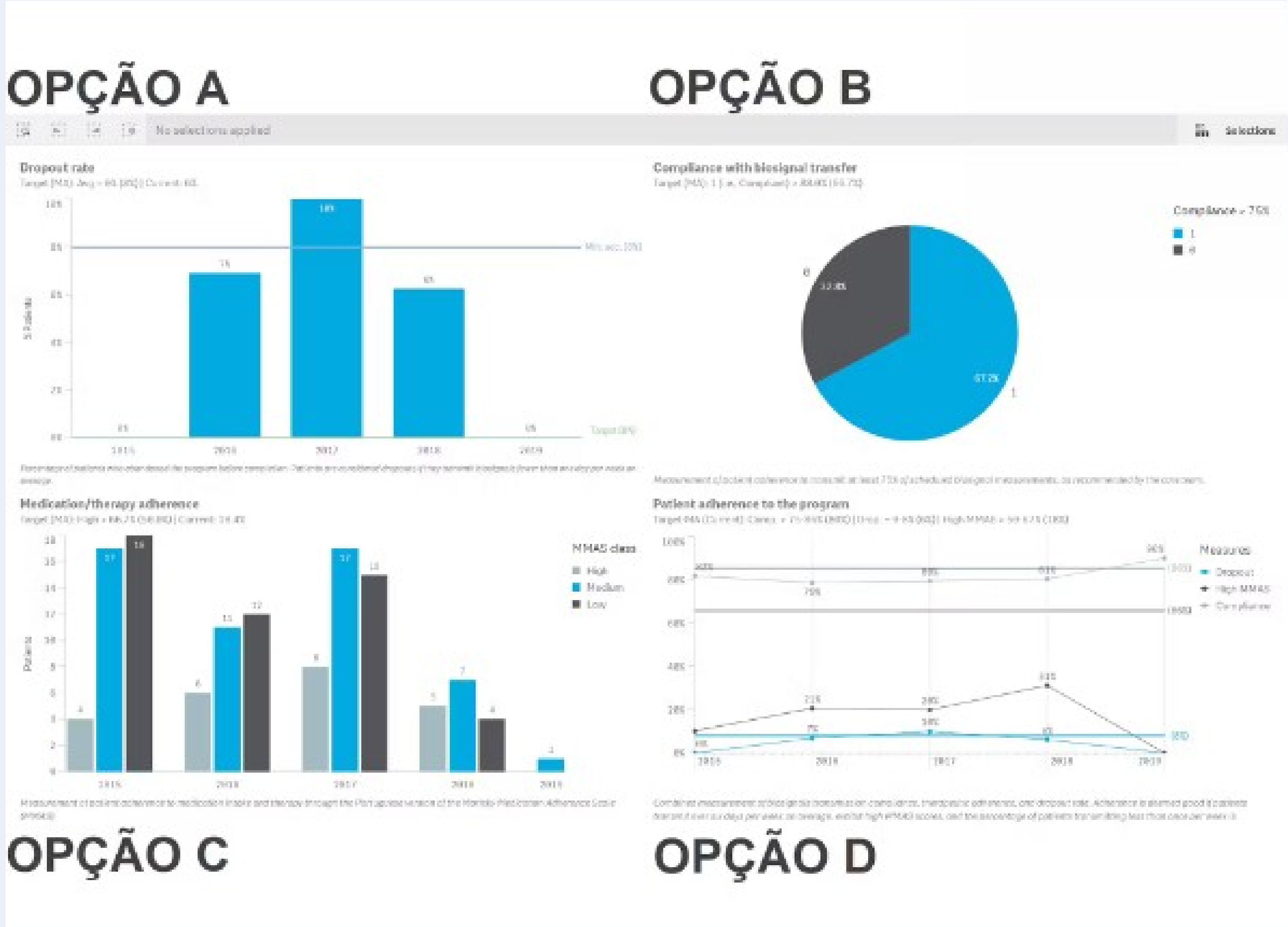

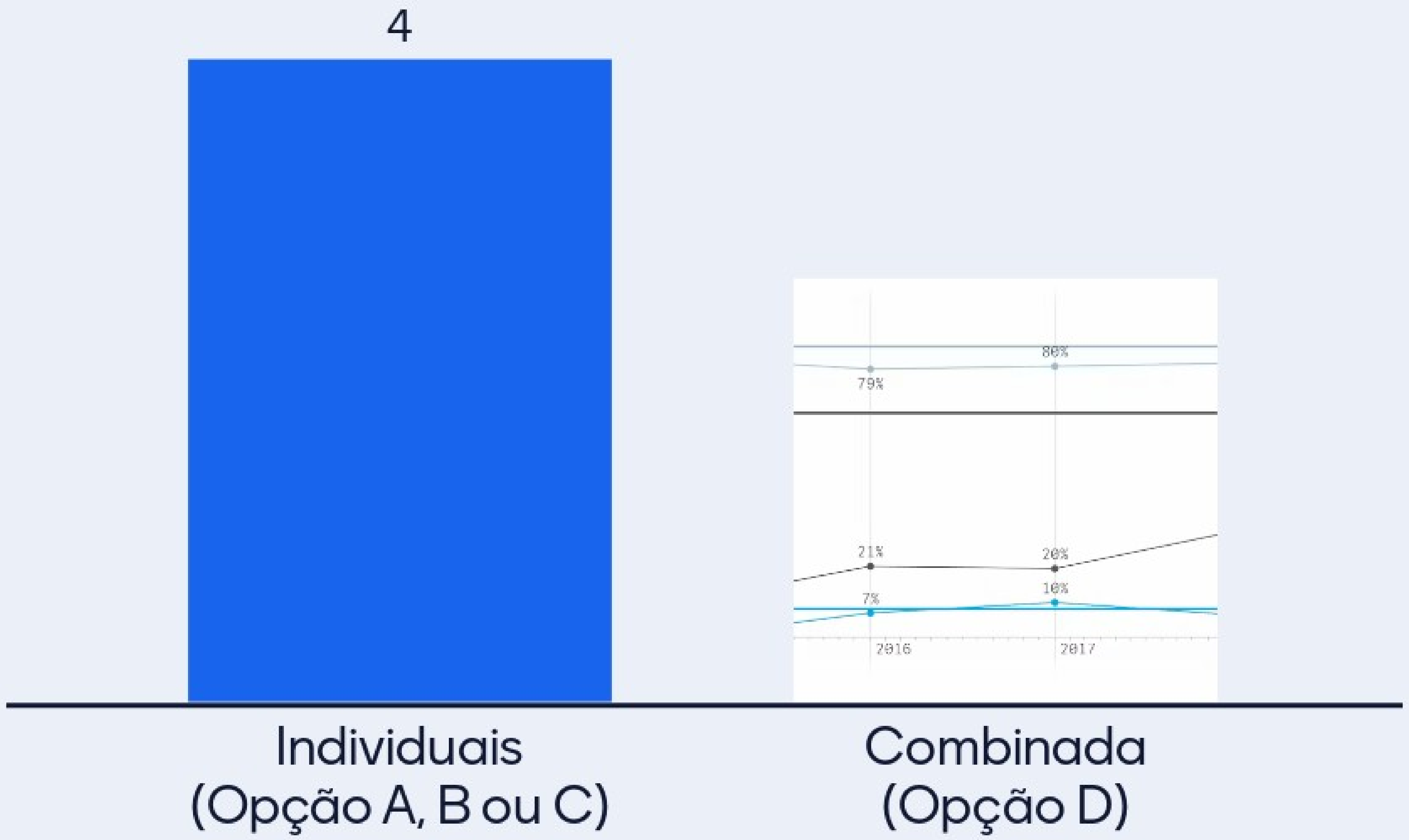

4

Para o KPI "Taxa de abandono" (Dropout rate), escolha uma das seguintes opções...

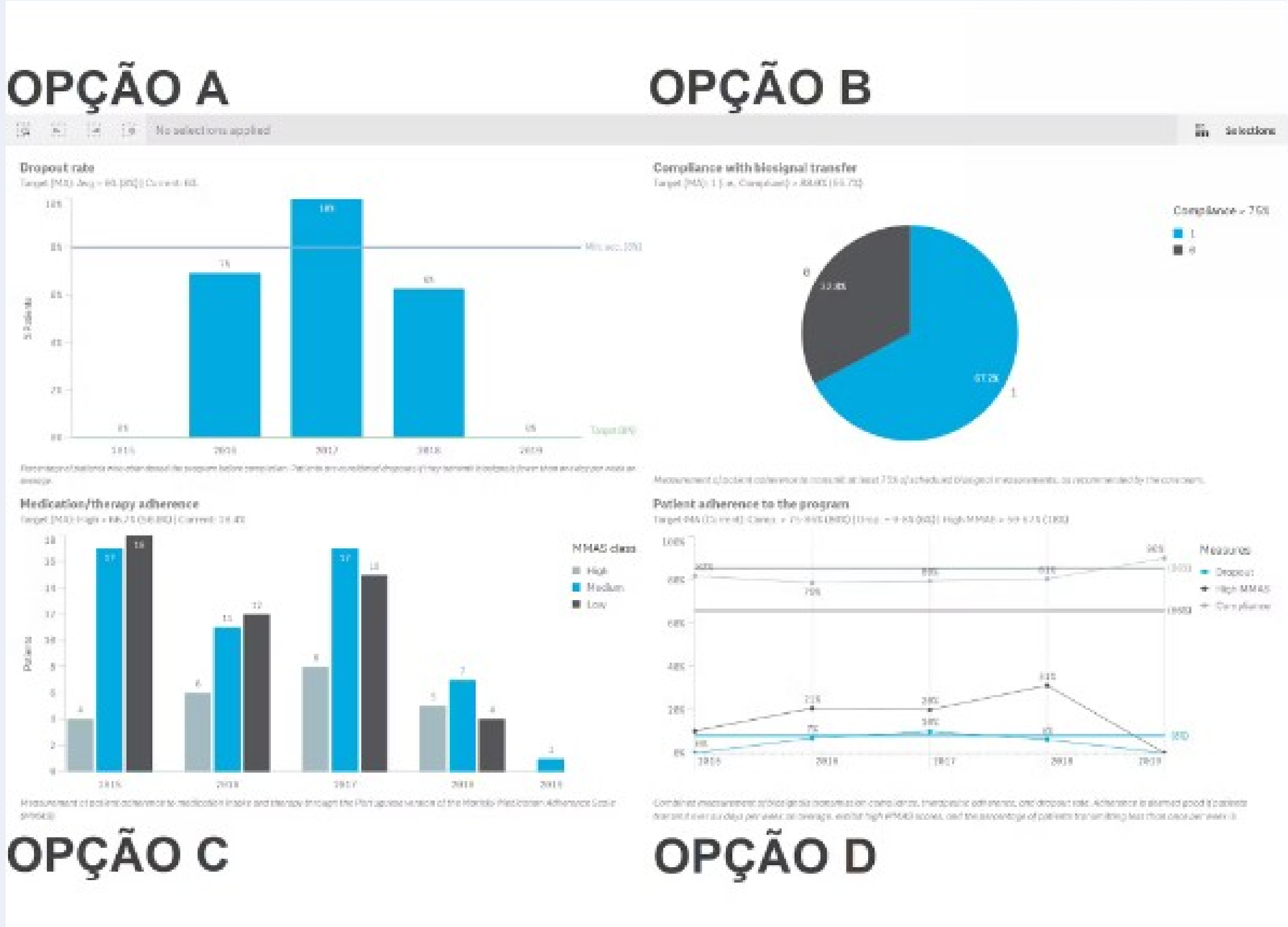

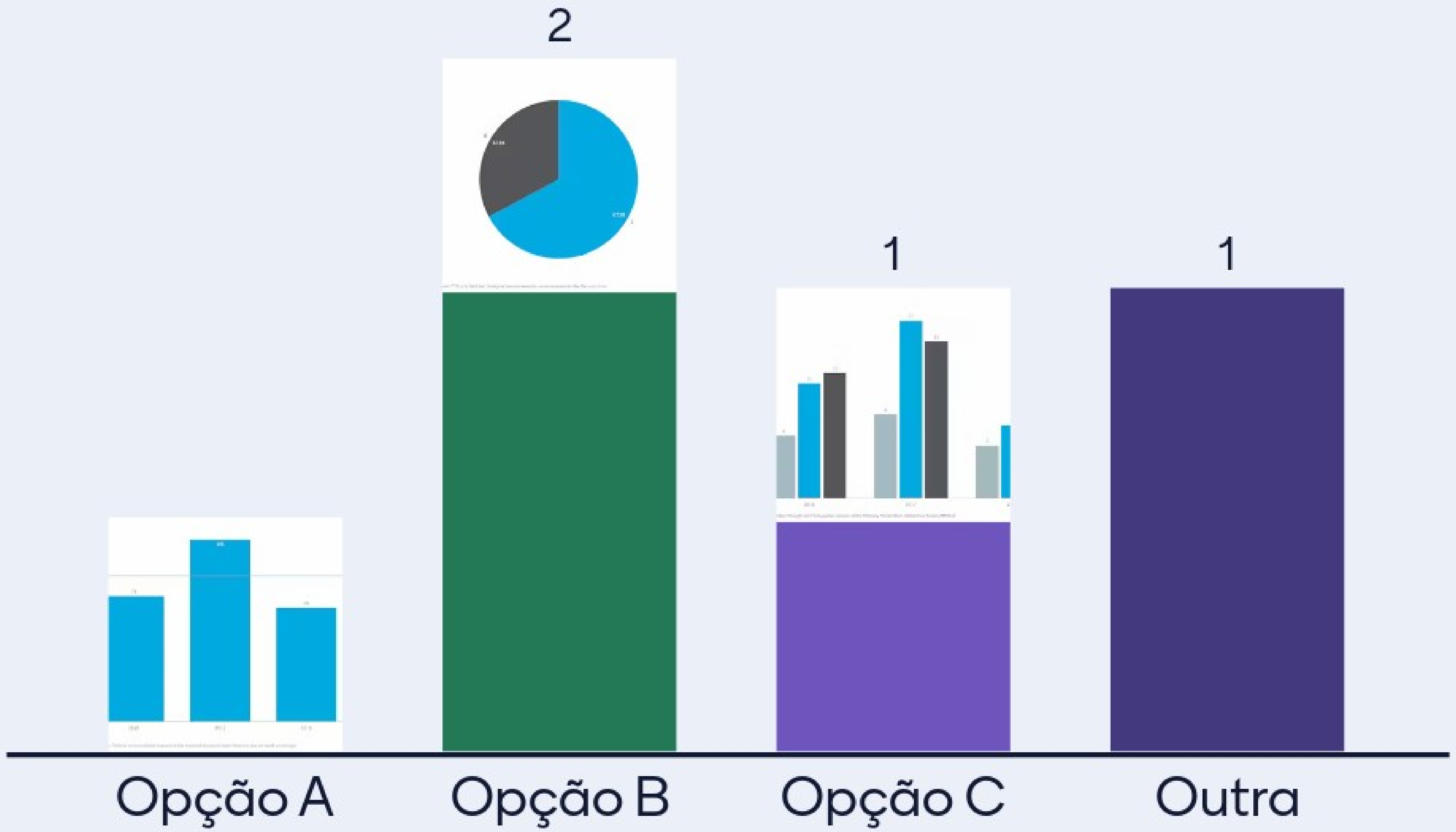

4

Para o KPI "Conformidade com a transmissão de biossinais" (Compliance), escolha uma das seguintes opções...

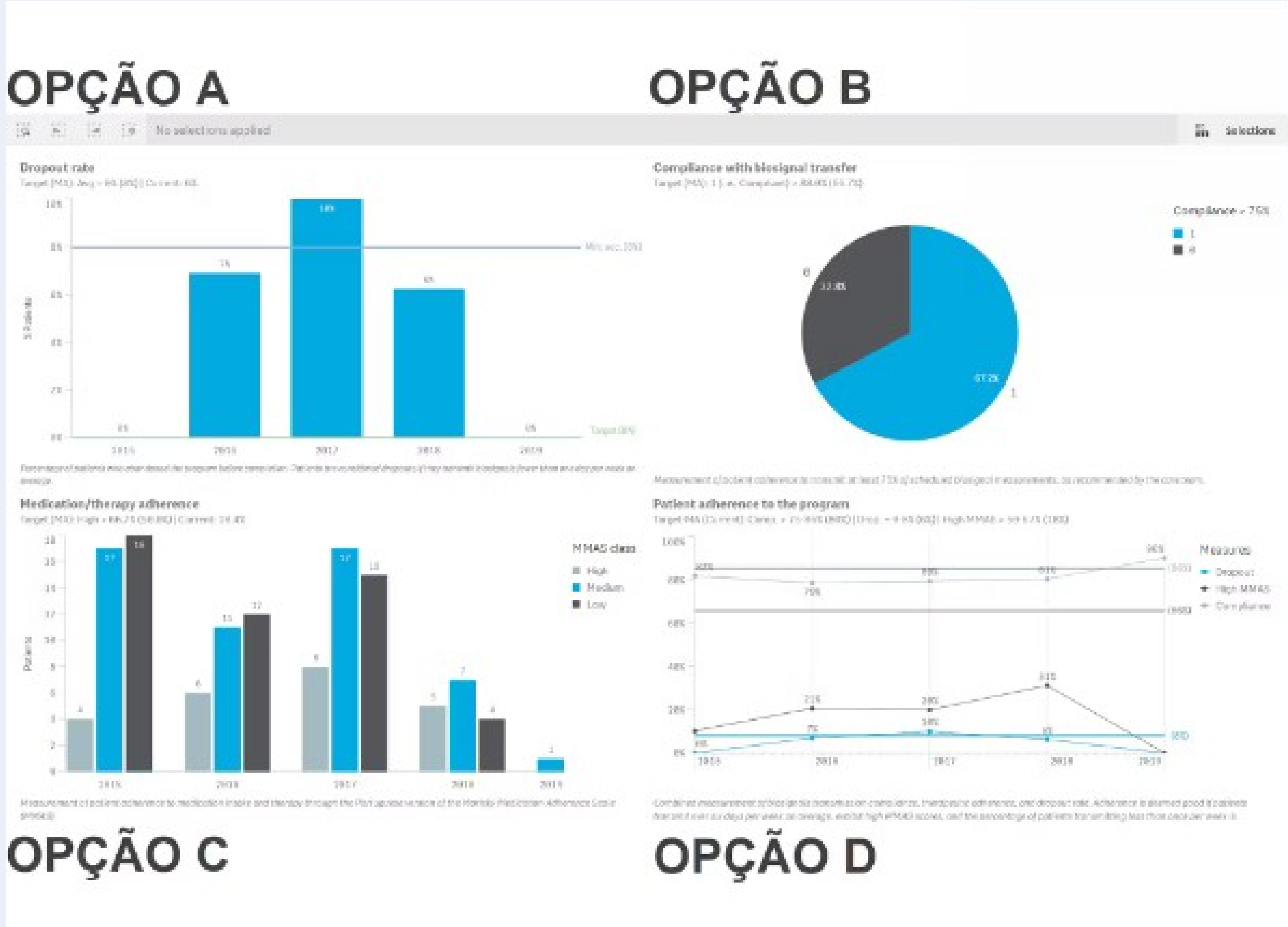

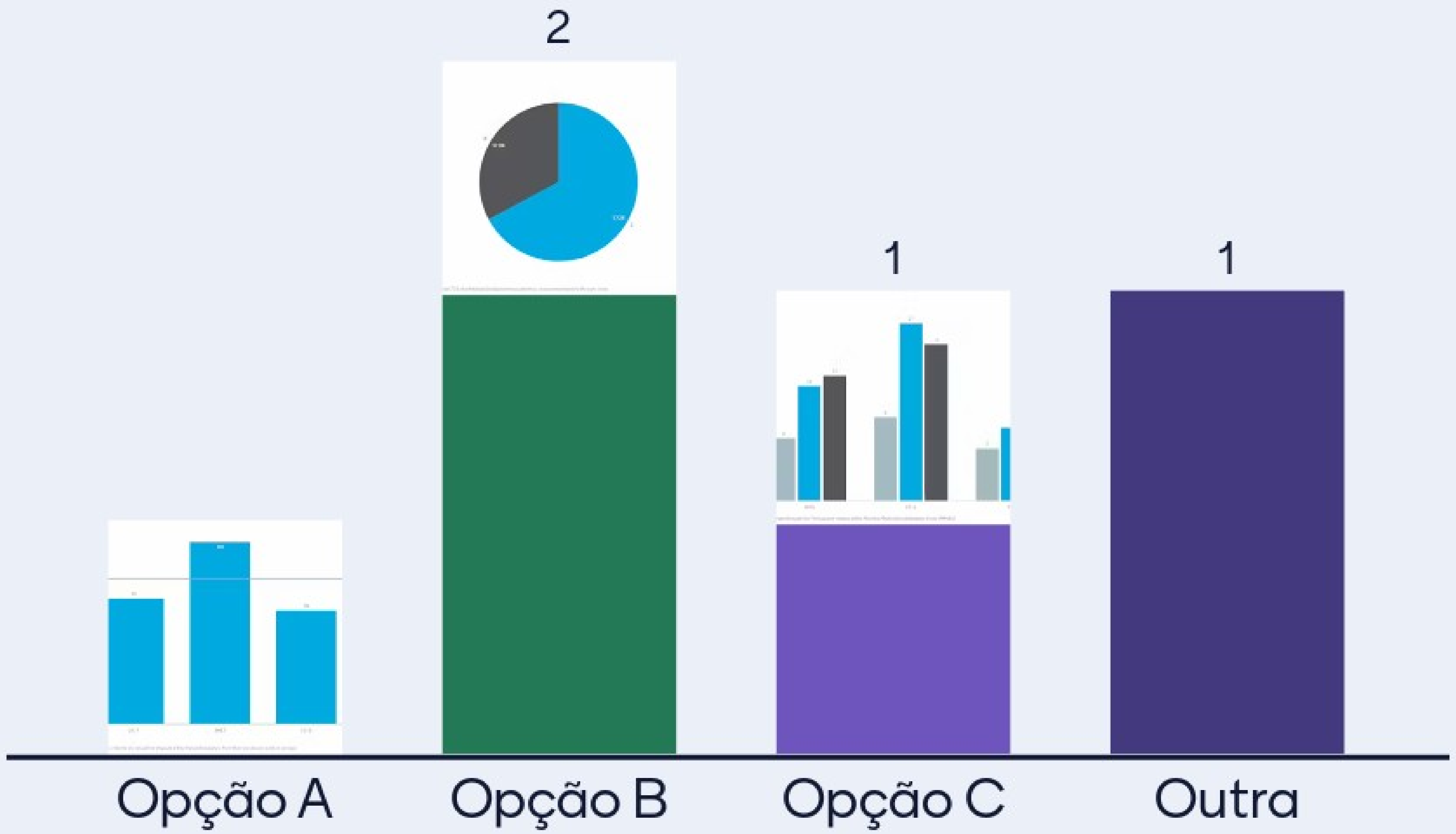

Para o KPI "Adesão à terapêutica" (Medication/therapy adherence), escolha uma das seguintes opções...

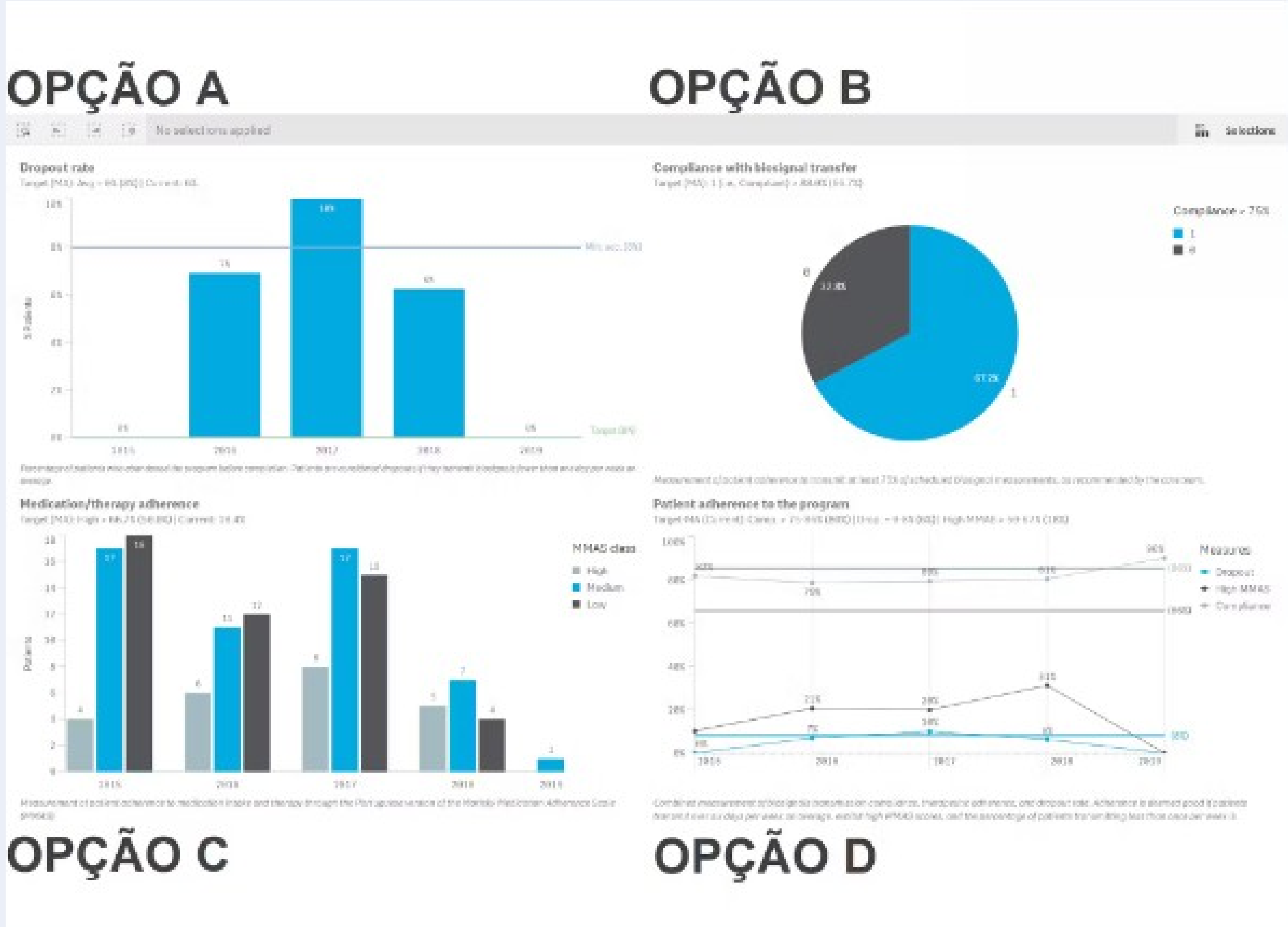

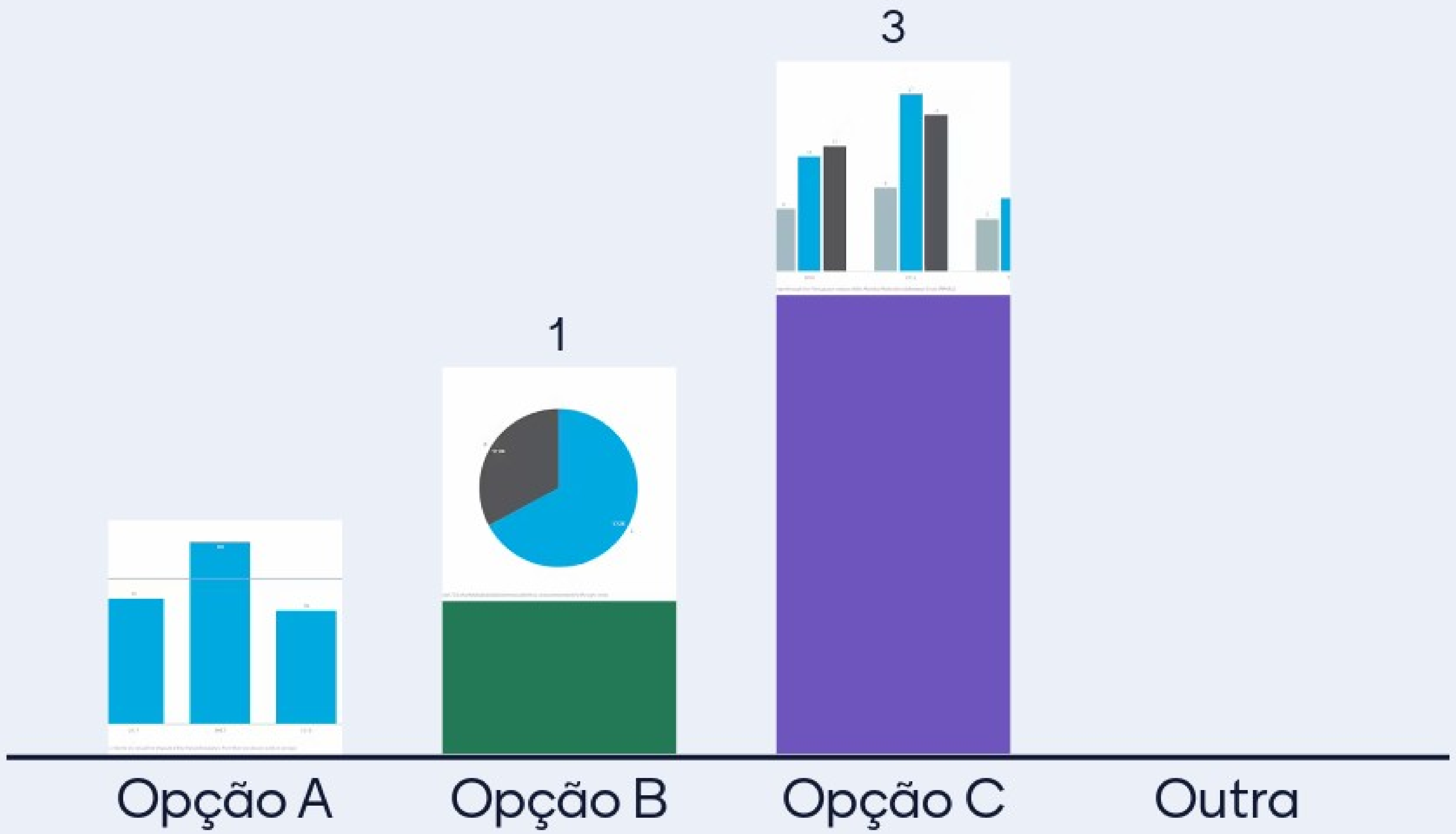

4

Comentários:

faculdade na leitura dos dados

Acho que tanto a A como a B são indicadas para os biossinais e adesão ao programa

Penso que gráfico de barras se torna mais legível e a distribuição por categorias pode ajudar na leitura.

3

Satisfação dos stakeholders

Inclui KPIs para satisfação do doente, do profissional de saúde e sobrecarga do cuidador. Escalas propostas: Doente: Home Monitoring Acceptance and Satisfaction Questionnaire (HoMASQ); Profissional de saúde: Job Satisfaction Survey (JSS); Cuidador: 4-item Zarit Burden Interview (ZBI-4).

Para os KPIs associados a "Satisfação dos stakeholders", prefere visualizações individuais ou combinada?

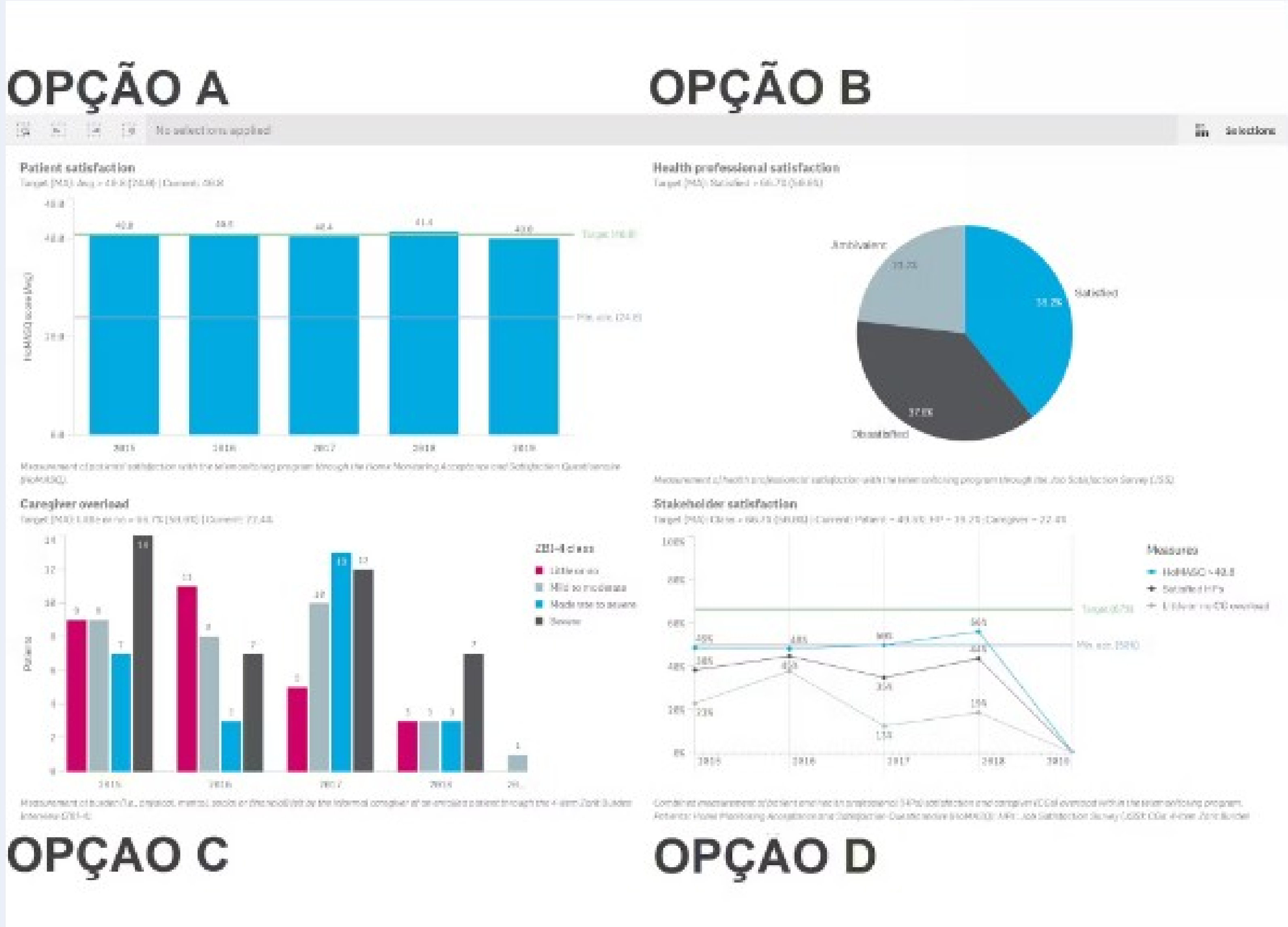

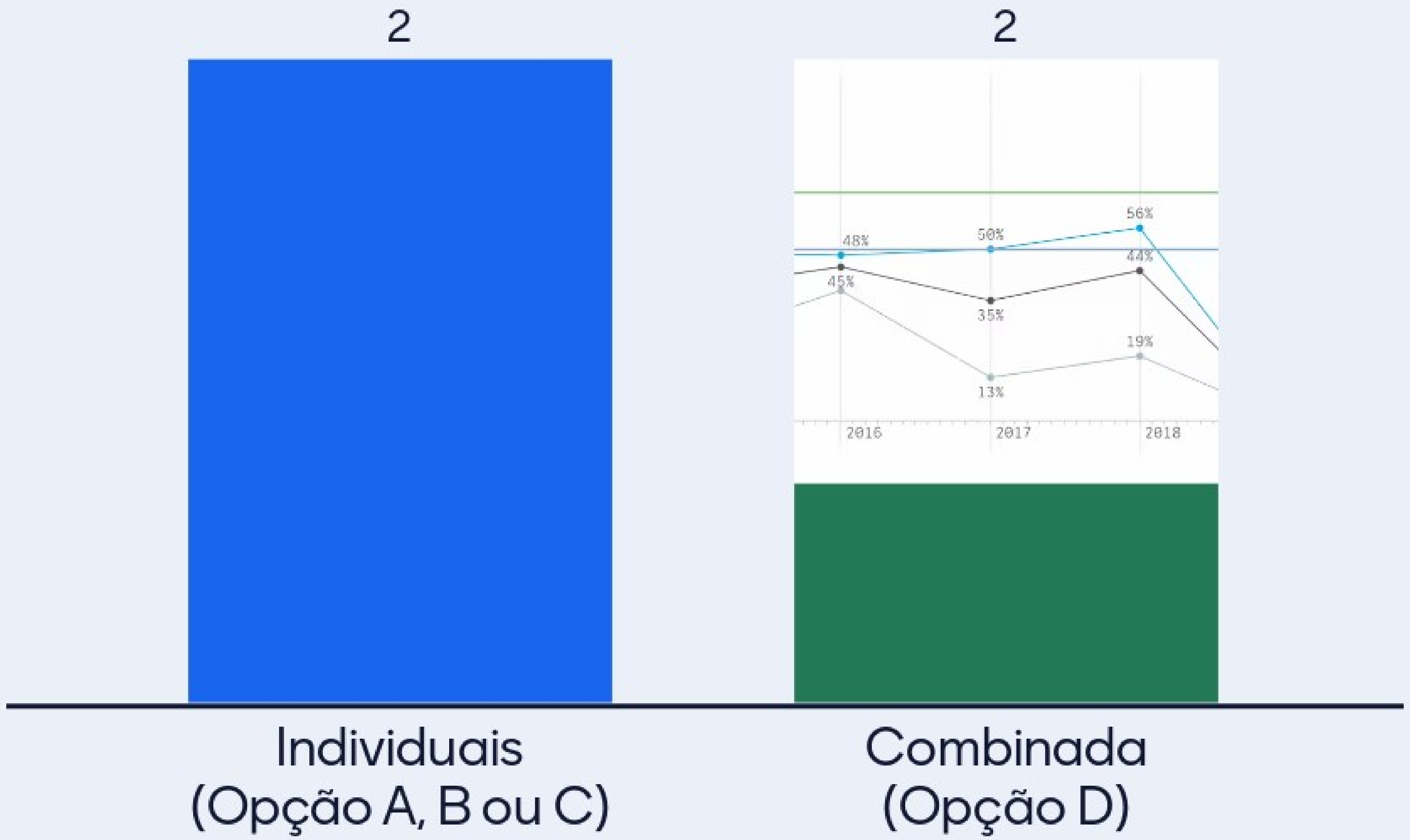

Para o KPI "Satisfação do doente" (Patient satisfaction), escolha uma das seguintes opções...

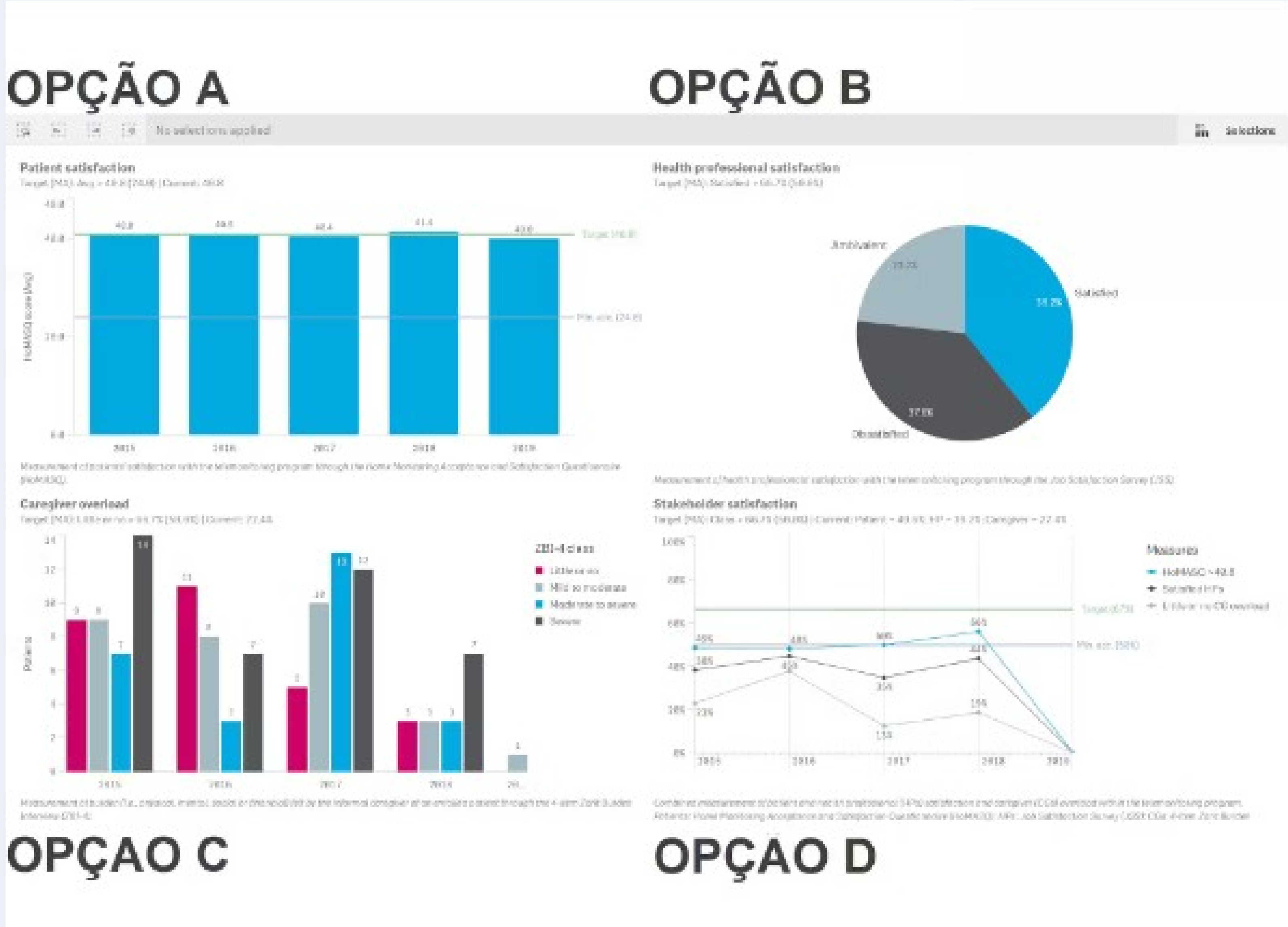

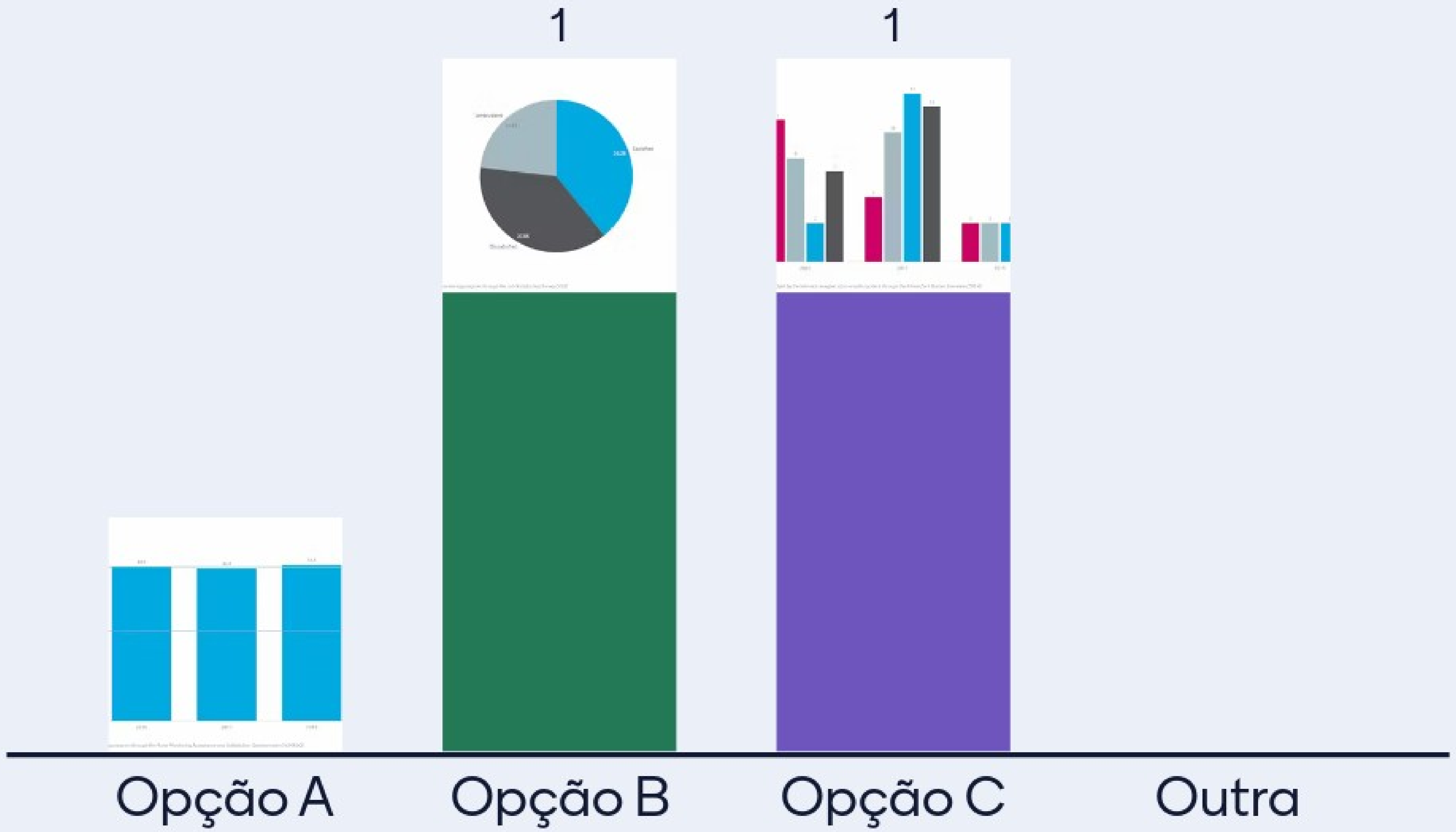

Para o KPI "Satisfação do profissional de saúde" (Health professional satisfaction), escolha uma das seguintes opções...

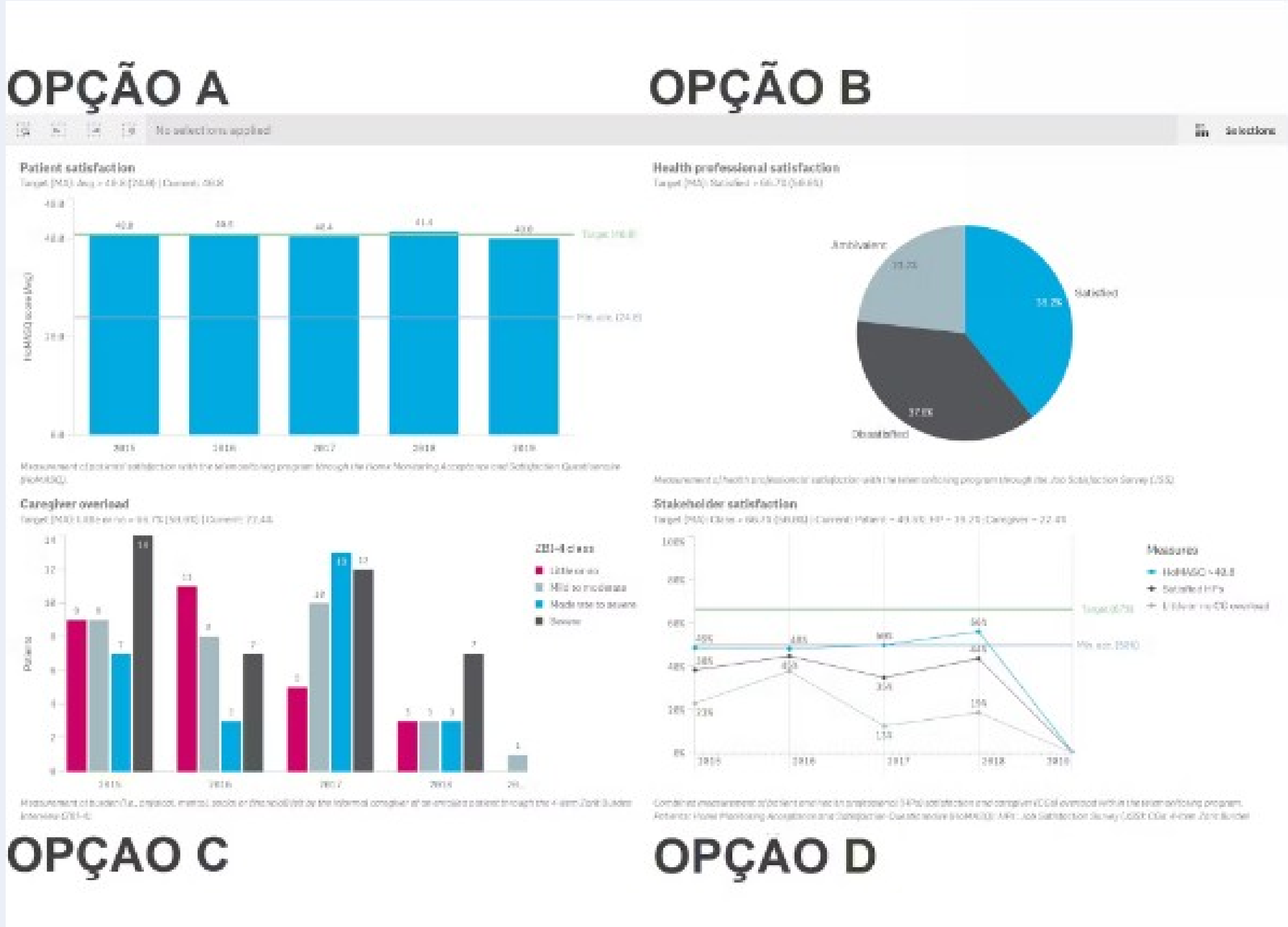

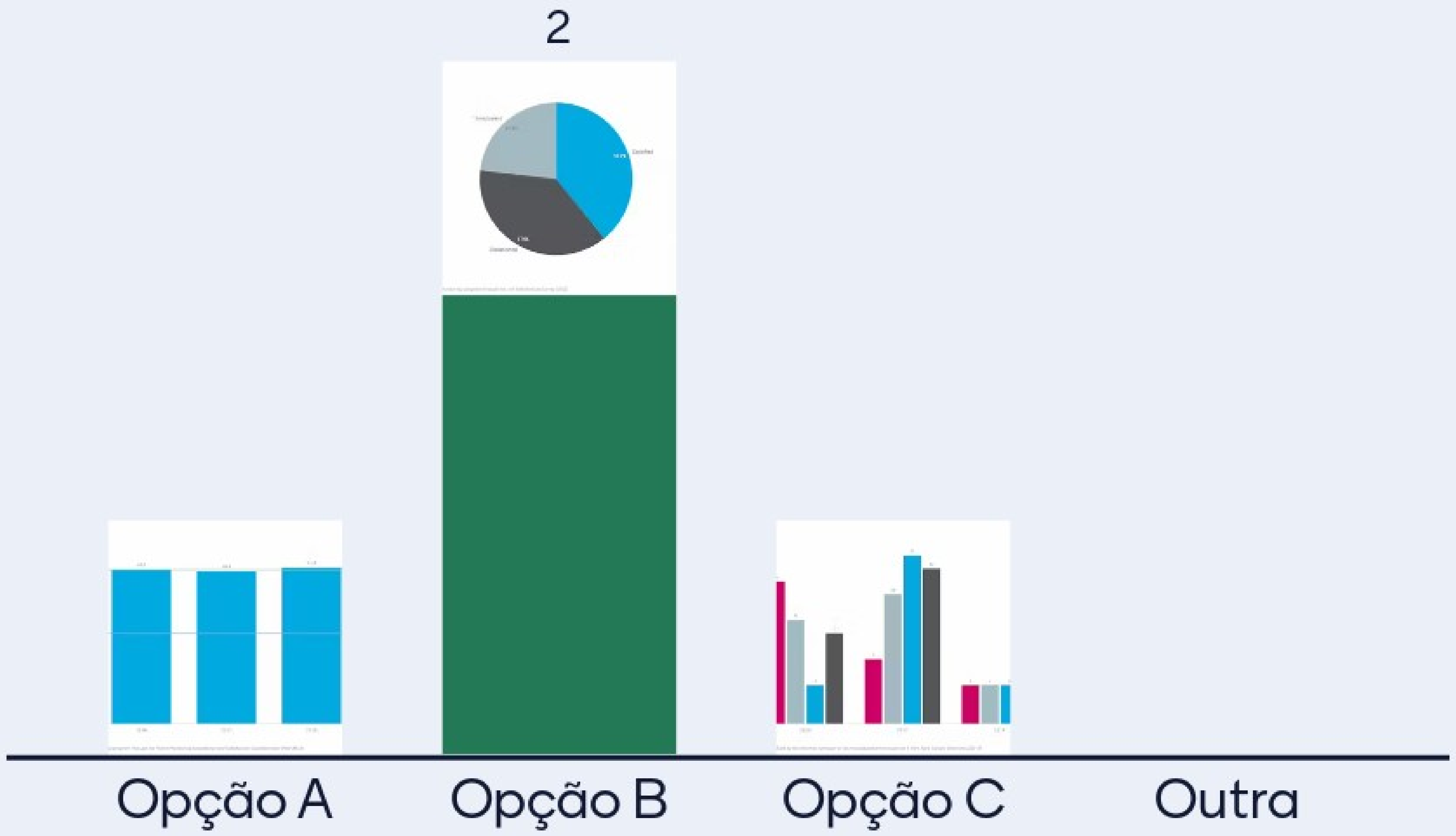

Para o KPI "Sobrecarga do cuidador" (Caregiver overload), escolha uma das seguintes opções...

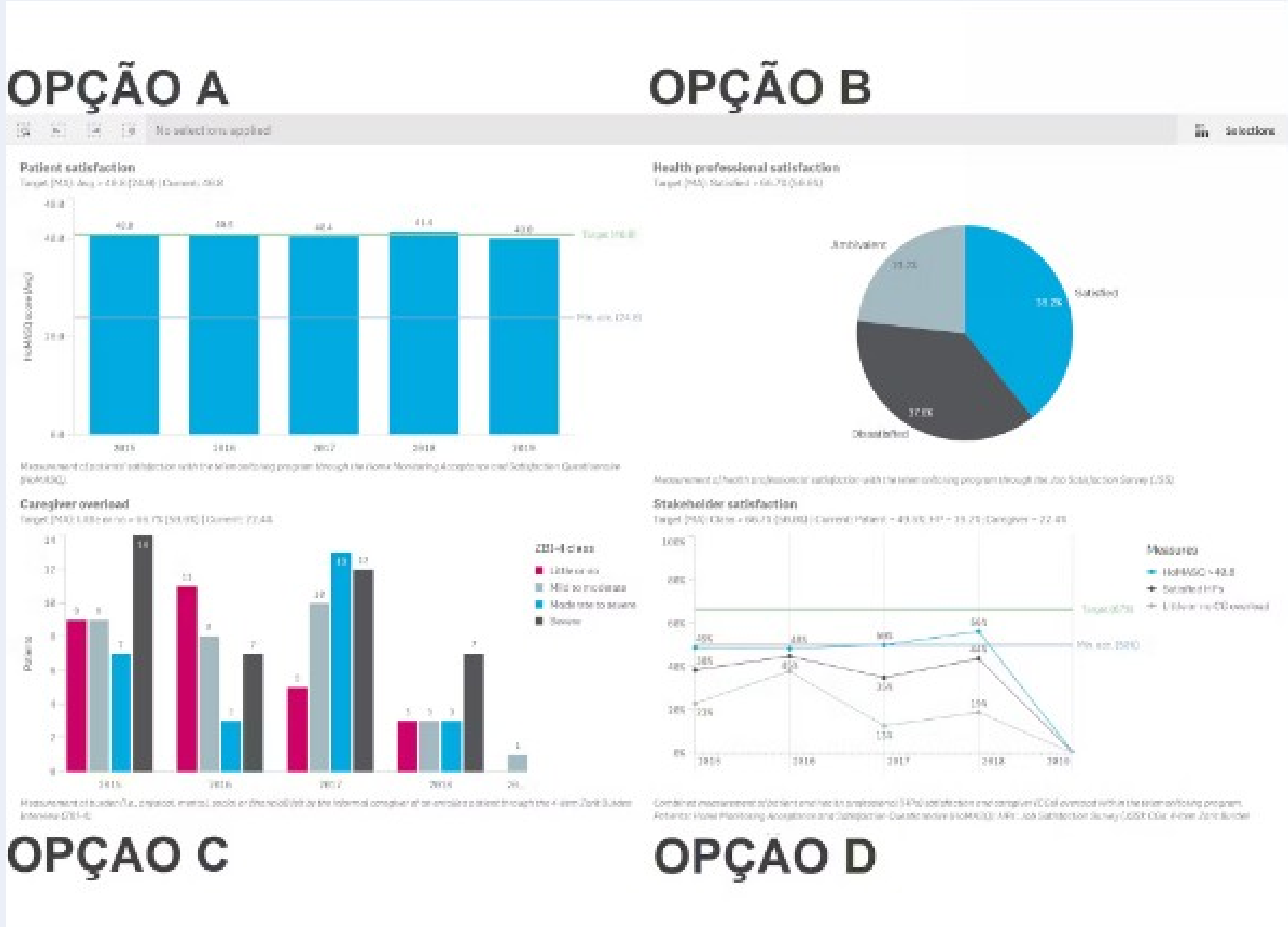

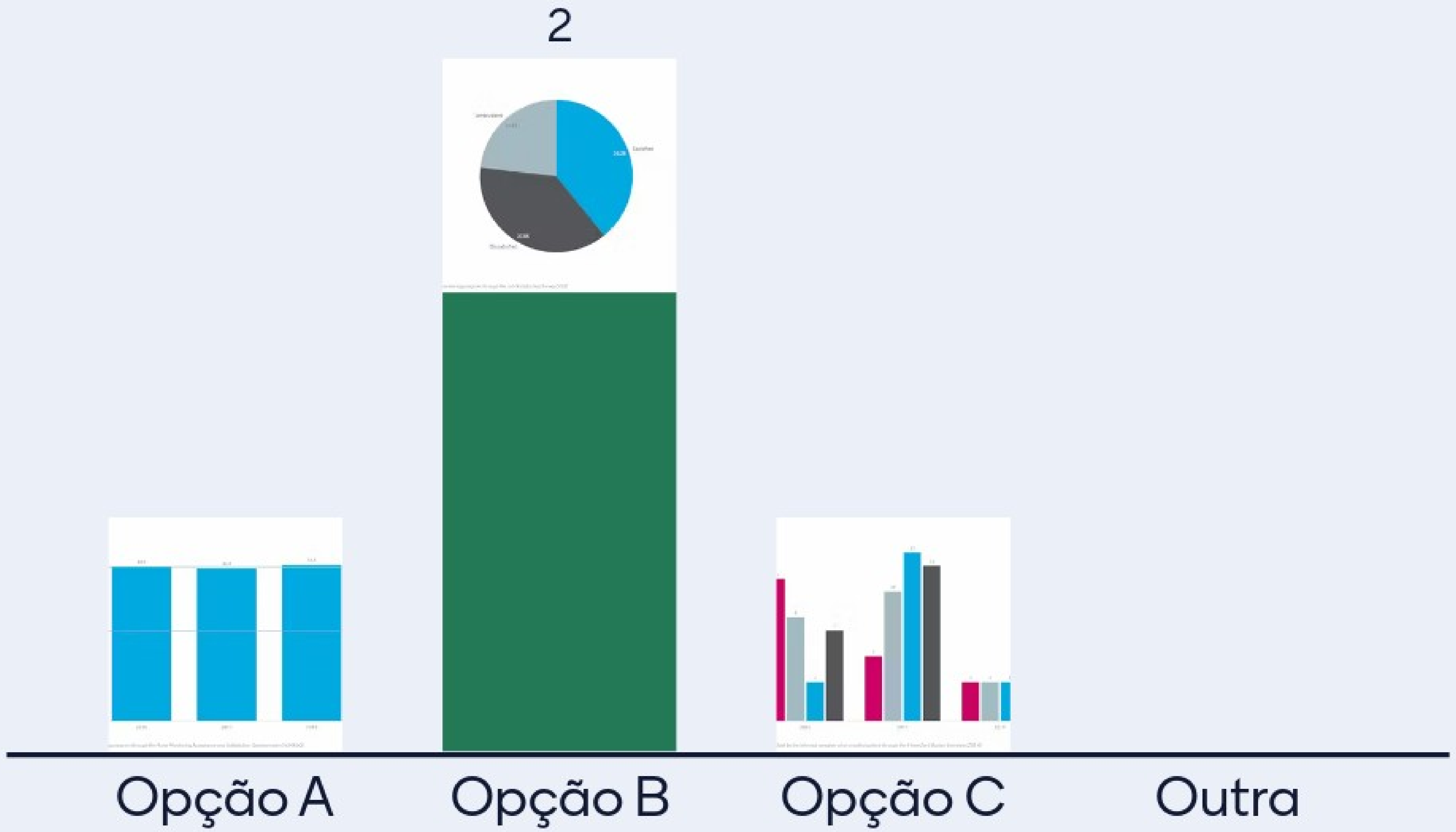

2

Comentários:

Maior facilidade em correlacionar variáveis

Acho que aqui a forma combinada é mais fácil de visualizar

Neste caso, penso que a interpretação em função do total, seja mais legível neste tipo de gráfico

3

Capacidade de autocuidado

Inclui KPIs para capacidade de gestão da doença e nível de autocuidado. Escalas propostas: Gestão da doença: Kansas City Cardiomyopathy Questionnaire (Self-efficacy) (KCCQ [Self-eff.]); Autocuidado: 13-item Patient Activation Measure (PAM13).

Para os KPIs associados a "Capacidade de autocuidado", prefere visualizações individuais ou combinada?

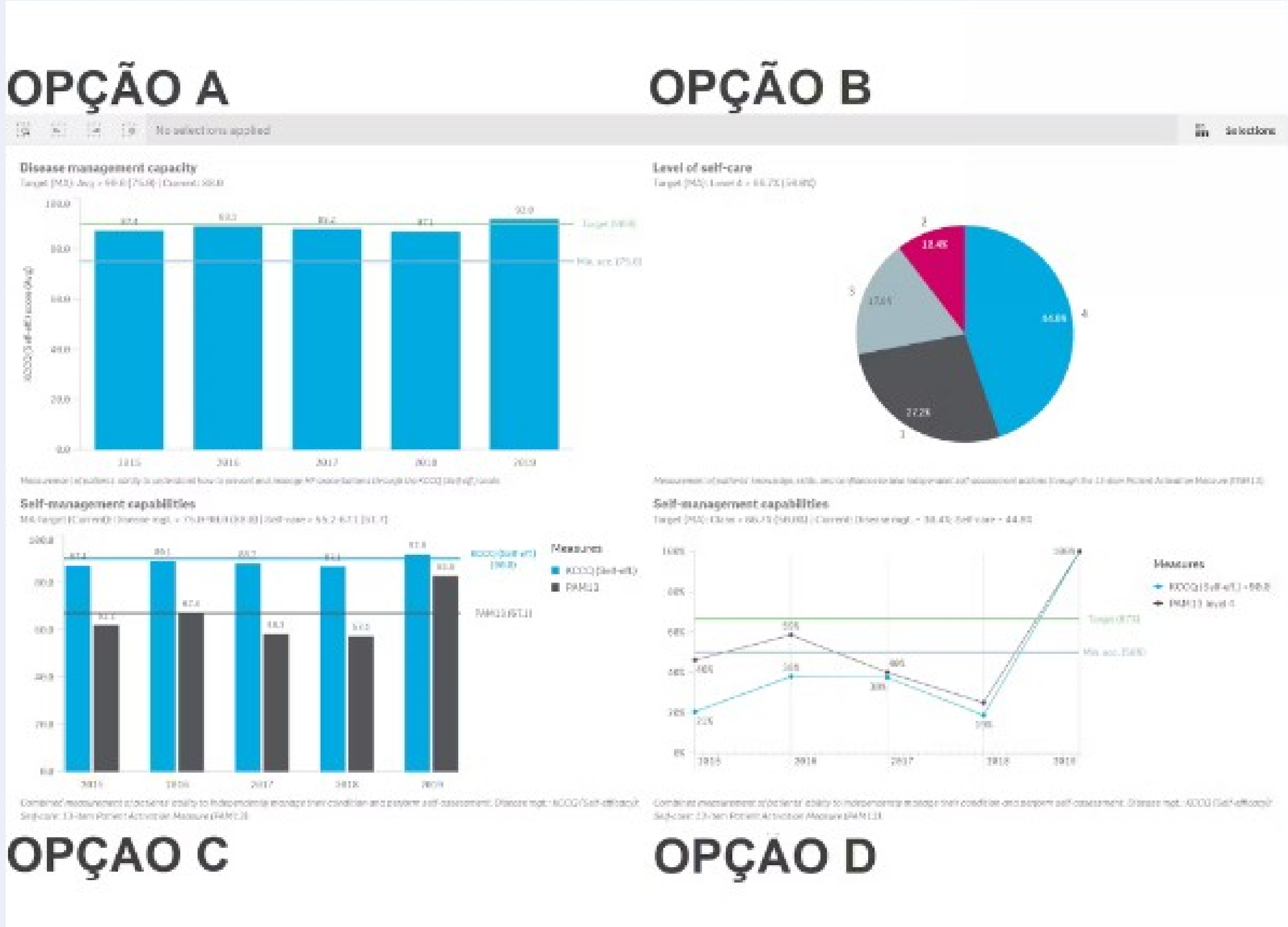

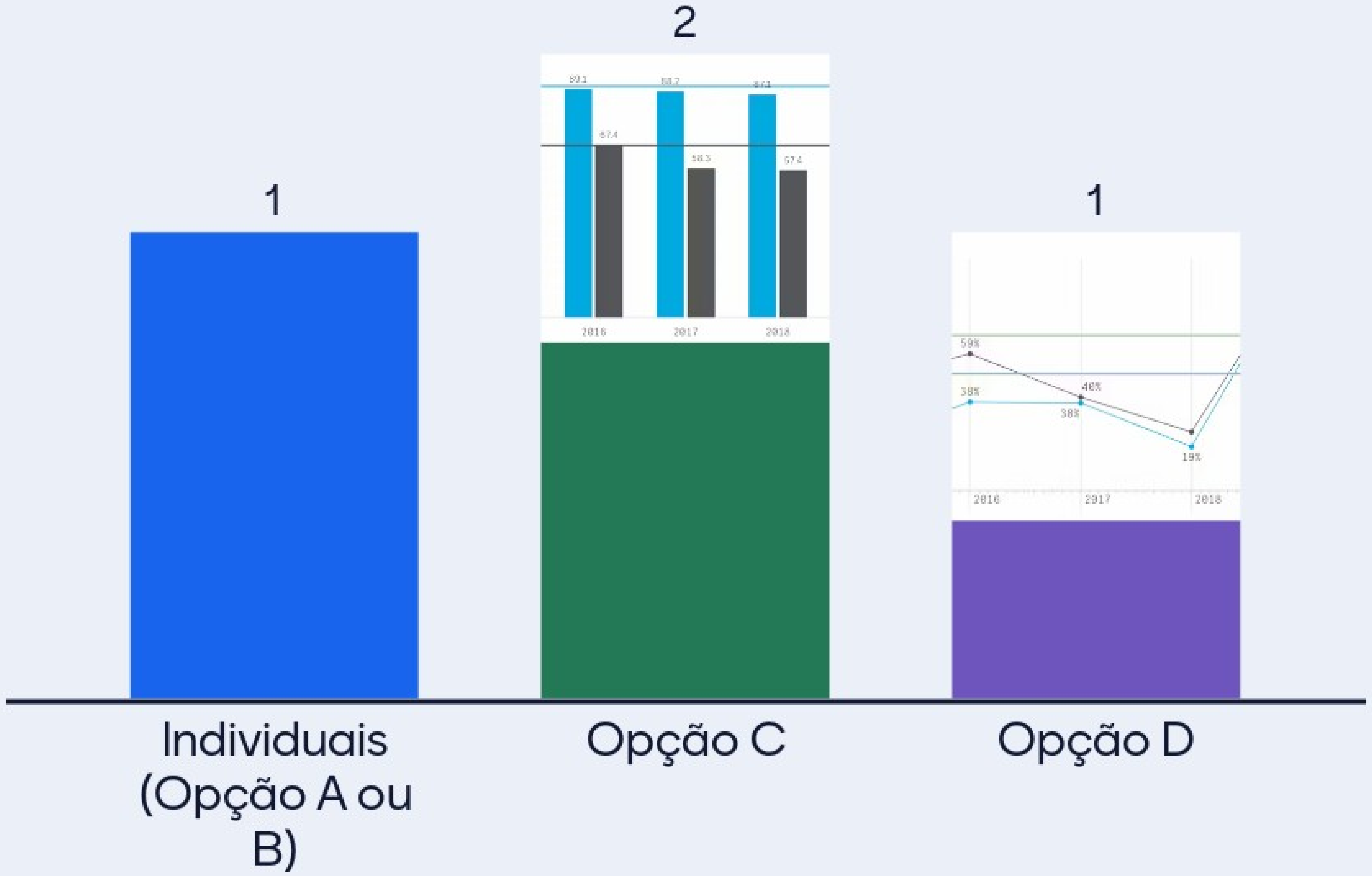

Para o KPI "Capacidade de gestão da doença" (Disease management capacity), escolha uma das seguintes opções...

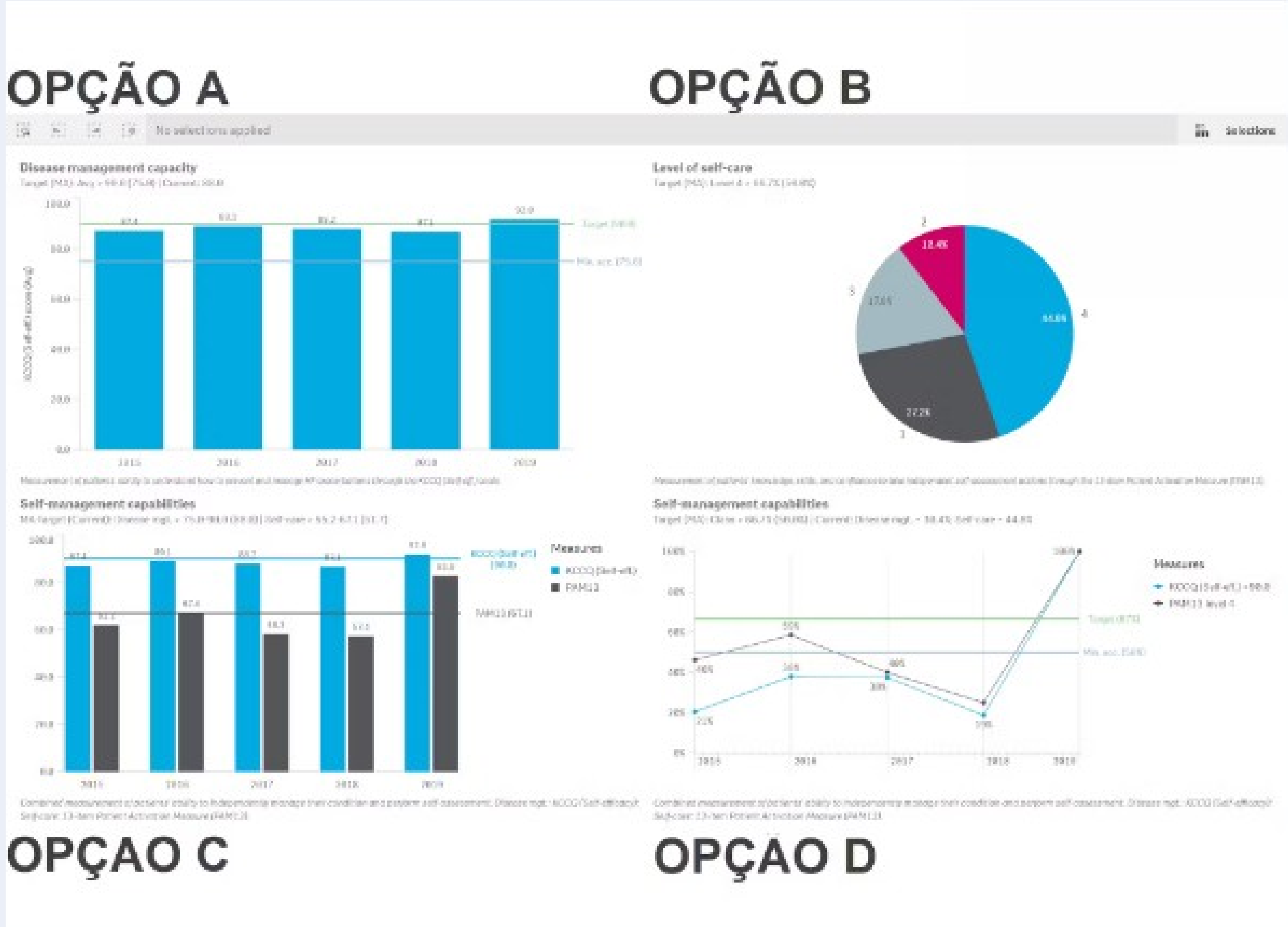

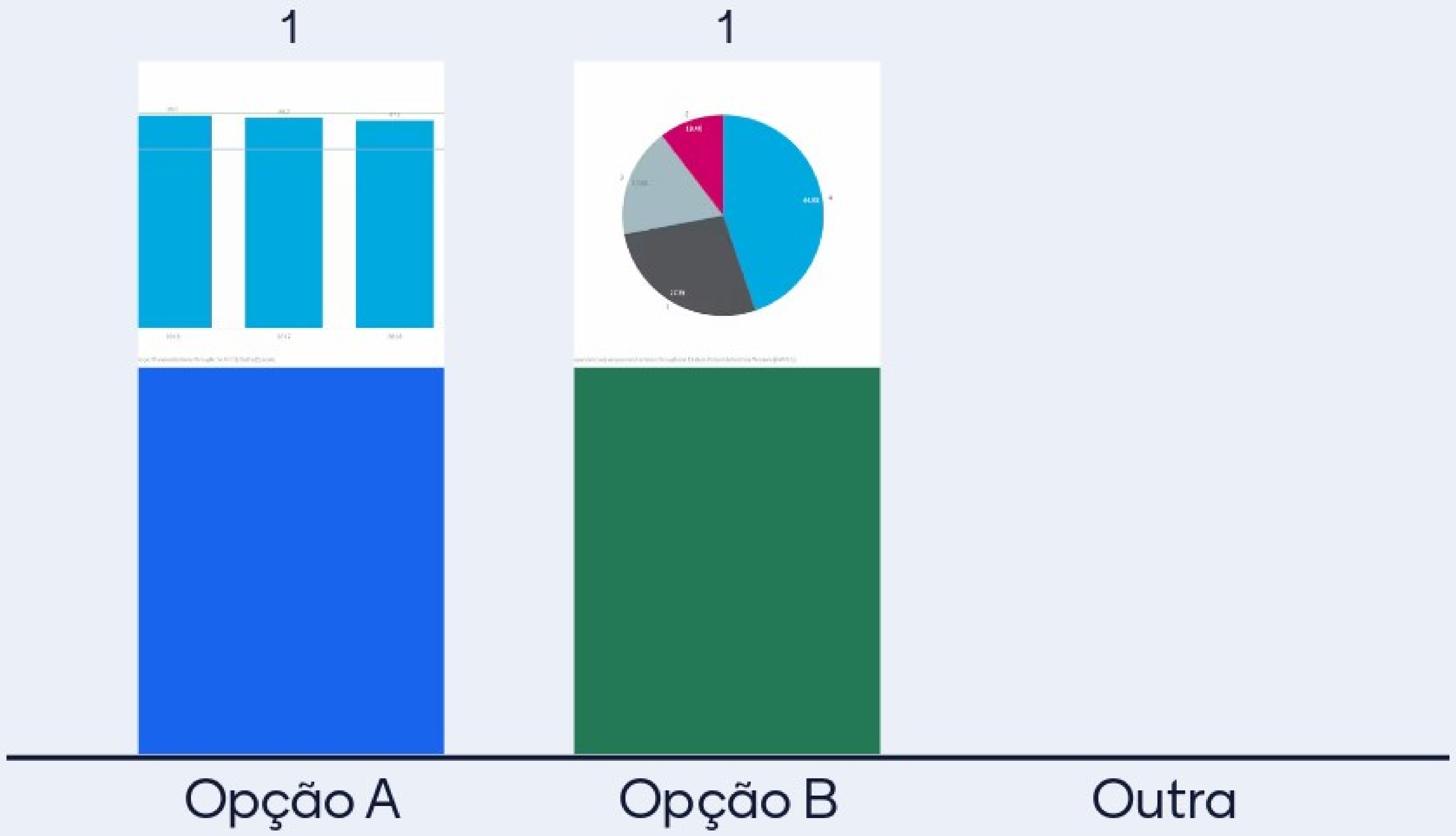

Para o KPI "Nível de autocuidado" (Level of self-care), escolha uma das seguintes opções...

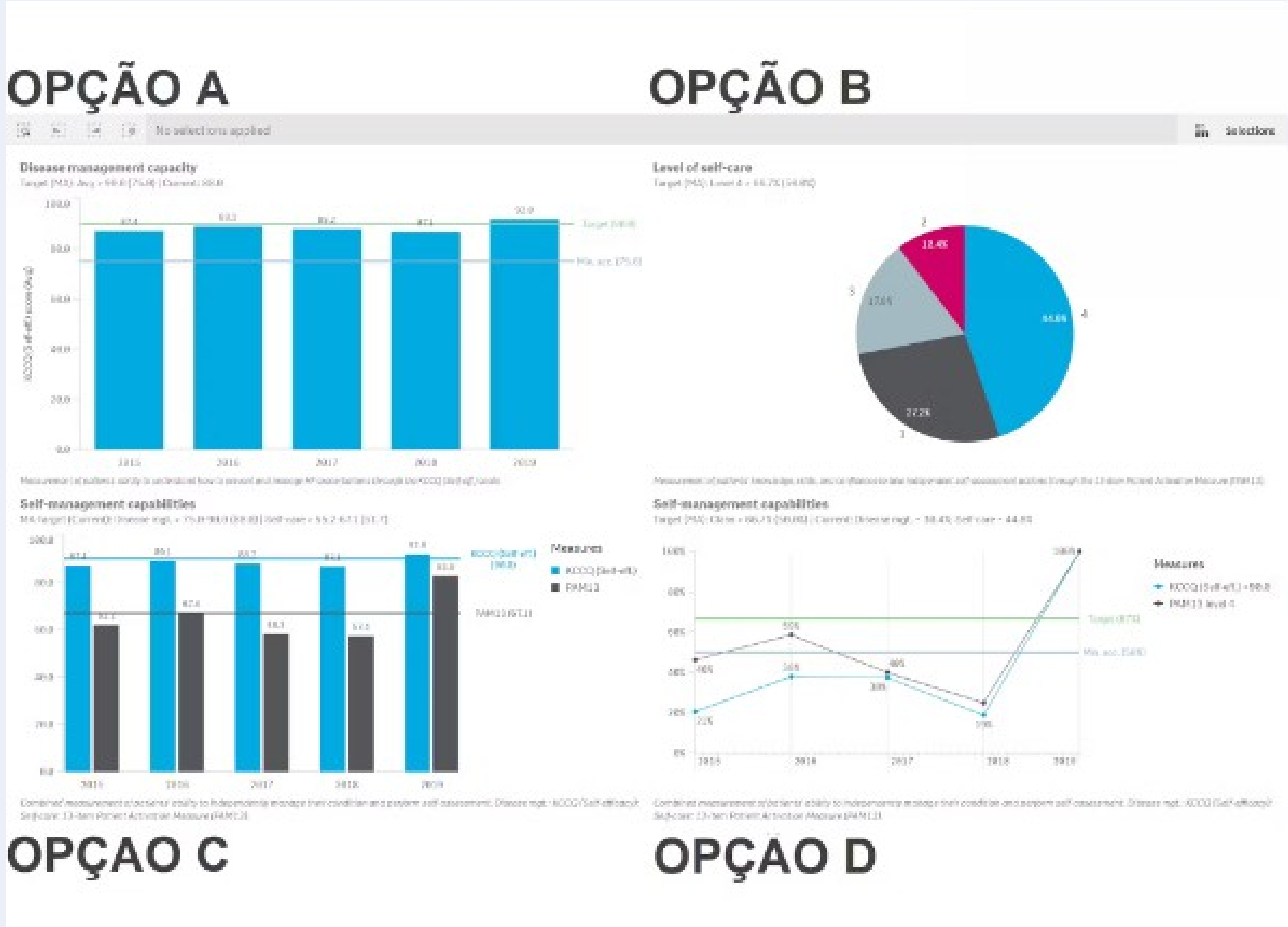

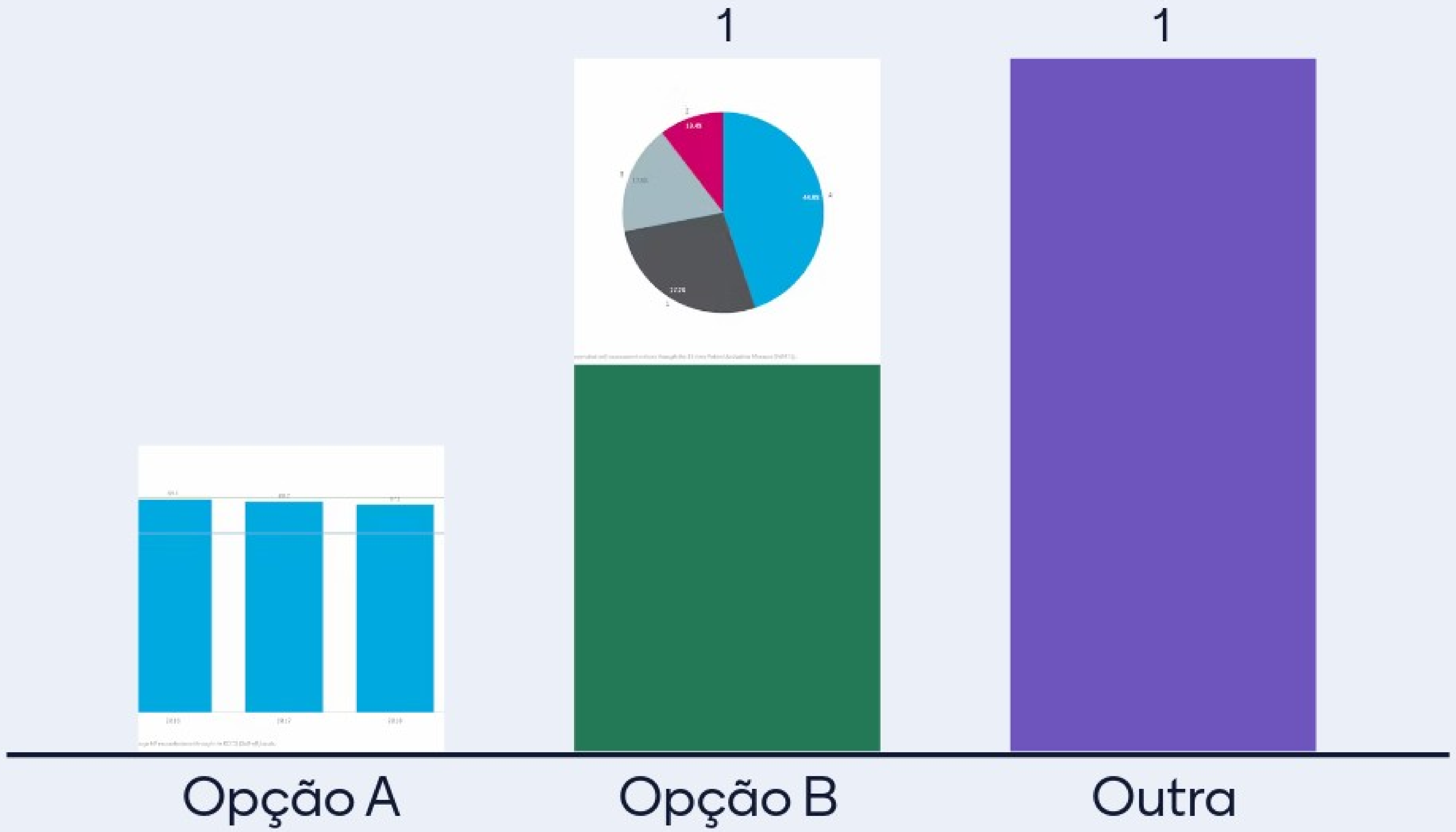

Comentários:

Fiquei com ideia na última reunião que para este indicador íamos usar apenas a parte de KCCQ que diz respeito a gestão da doença e autocuidado... acho que fica melhor representado no gráfico da opção D

Mesmo que seja só o Kansas (continuação da resposta anterior)

Acho que neste ponto, ficaria melhor agrupar. Enganei-me a responder inicialmente e já não consegui voltar atrás

2

Confiança do doente no programa

Medida da confiança do doente na eficácia e adequação do programa.. Escala proposta: Escala de confiança de Dugan.

Para o KPI "Confiança do doente no programa" (Patient's trust in the program), escolha uma das seguintes opções...

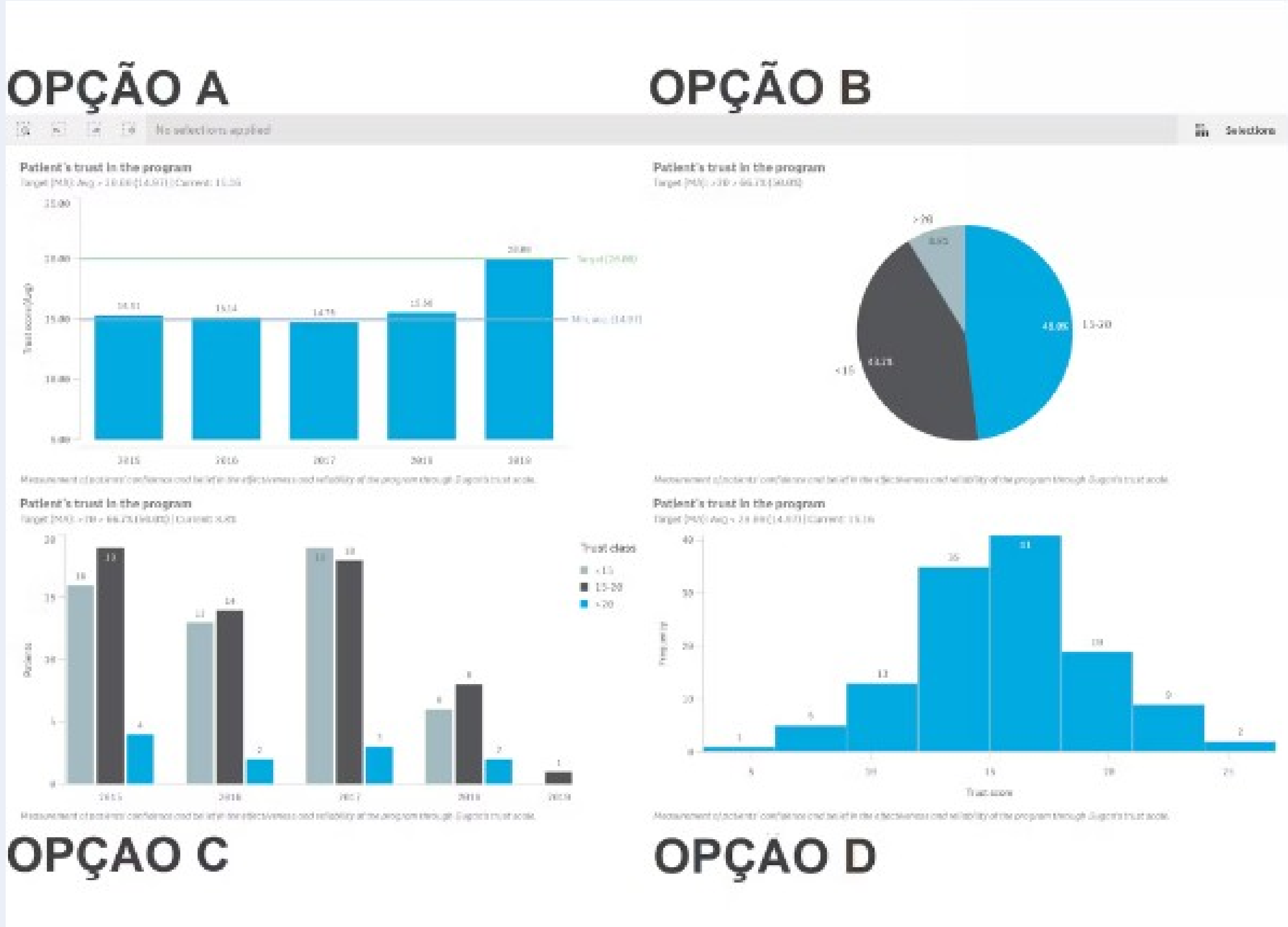

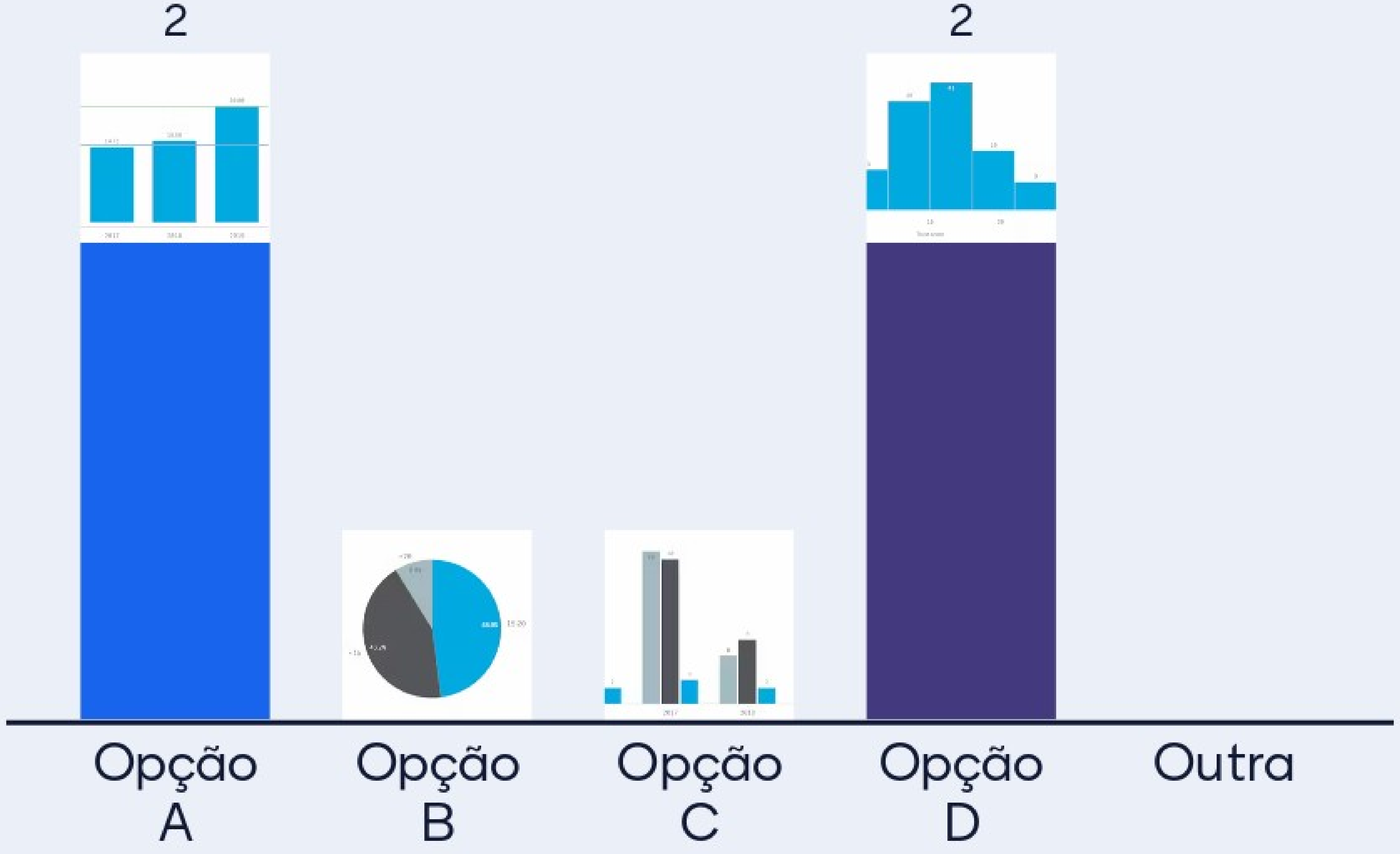

4

Comentários:

Melhor ideia de contínuo temporal vs opção A

Acho bem ter a linha do "Cuf-off" ideal

A opção A permite obter uma perspectiva de evolução ao longo dos anos.

3

Obrigado por participar!
Encontramos-nos no CDB workshop!
(Não se esqueça de responder ao Doodle)
